# Supplementary material for: ERFVIIs as transducers of oxygen-sensing in the evolution of land plant response to hypoxia
Source: Mol Plant. 2025 Jun 2;18(6):1072–87. doi: 10.1016/j.molp.2025.05.015 (PMC13448761; doi:10.1016/j.molp.2025.05.015)
Supplement: Supplementary file 1 [file mmc1.pdf]

Molecular Plant, Volume *18*

Supplemental information

ERFVIIs as transducers of oxygen-sensing in the evolution of land plant response to hypoxia

Laura Dalle Carbonare, Hans van Veen, Vinay Shukla, Monica Perri, Liem Bui, Michael J. Holdsworth, and Francesco Licausi

Supplemental Information

ERFVIIs as transducers of oxygen-sensing in the evolution of land plant response to hypoxia

Laura Dalle Carbonare 1,2, Hans van Veen 3, Vinay Shukla 1, Monica Perri 1, Liem Bui 4, Michael J. Holdsworth* 1 and Francesco Licausi* 2

Affiliations

1 School of Biosciences, University of Nottingham, Loughborough, LE12 5RD, UK

2 Department of Biology, University of Oxford, Oxford, OX1 3RB, UK

3 Groningen Institute of Evolutionary Life Sciences, University of Groningen, Groningen, NL

4 Biotechnology Research and Development Institute, Can Tho University, Can Tho, Vietnam

Contact information: Michael J. Holdsworth (michael.holdsworth@nottingham.ac.uk) and Francesco Licausi (francesco.licausi@biology.ox.ac.uk)

Additional footnotes: *Corresponding authors, equal contribution

Supplemental Figures

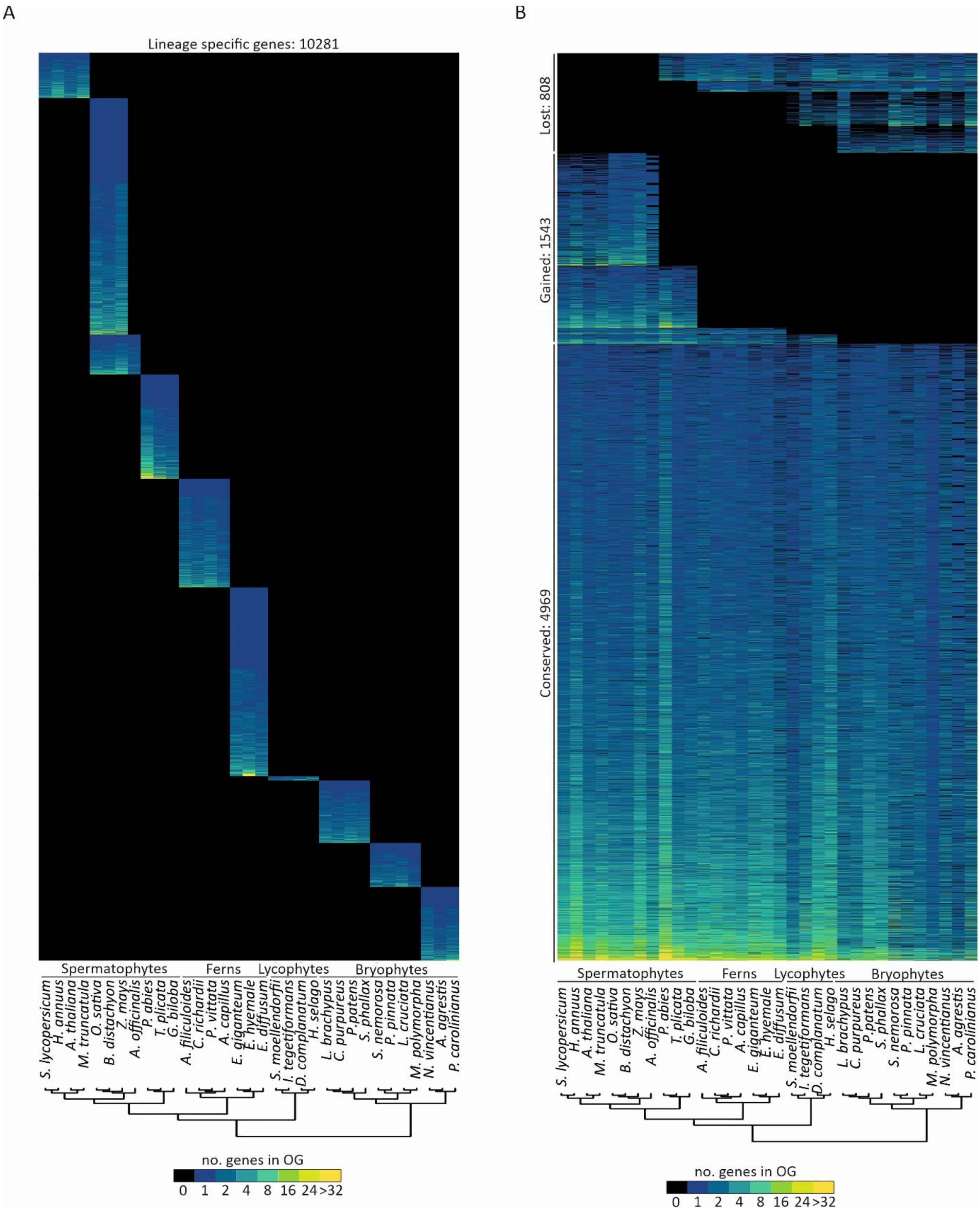

Supplemental Figure S1. **Distribution of genes across land plant evolution**. **A**) Hierarchical clustering of lineage specific genes (10281) from 33 land plant species, including those studied in this work: *M. polymorpha*, *P. patens*, *S. moellendorfii*, *E. hyemale*, *P. vittata*, *O. sativa* and *A. thaliana*. **B**) Conserved (4969), gained (1543) and lost (808) orthogroups from 33 land plant species.

A

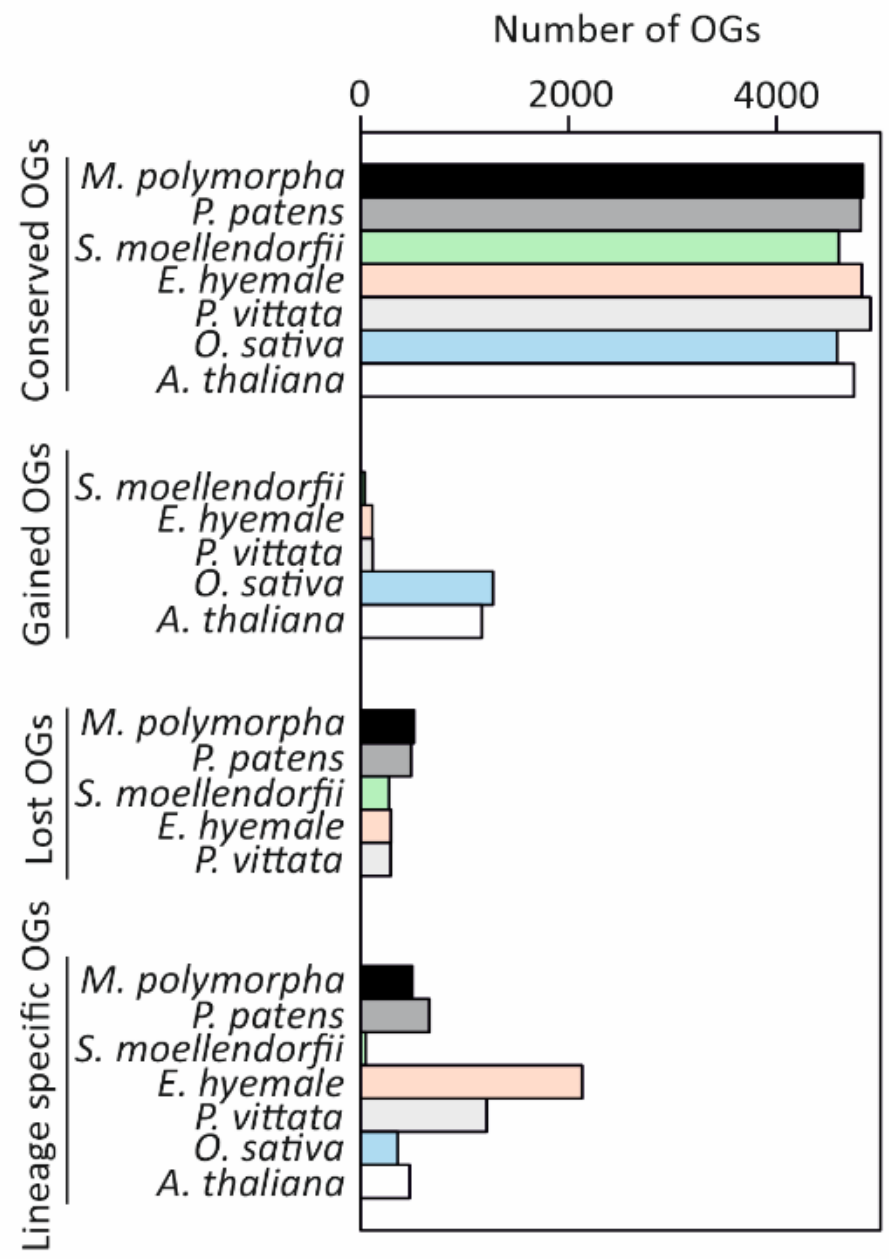

B

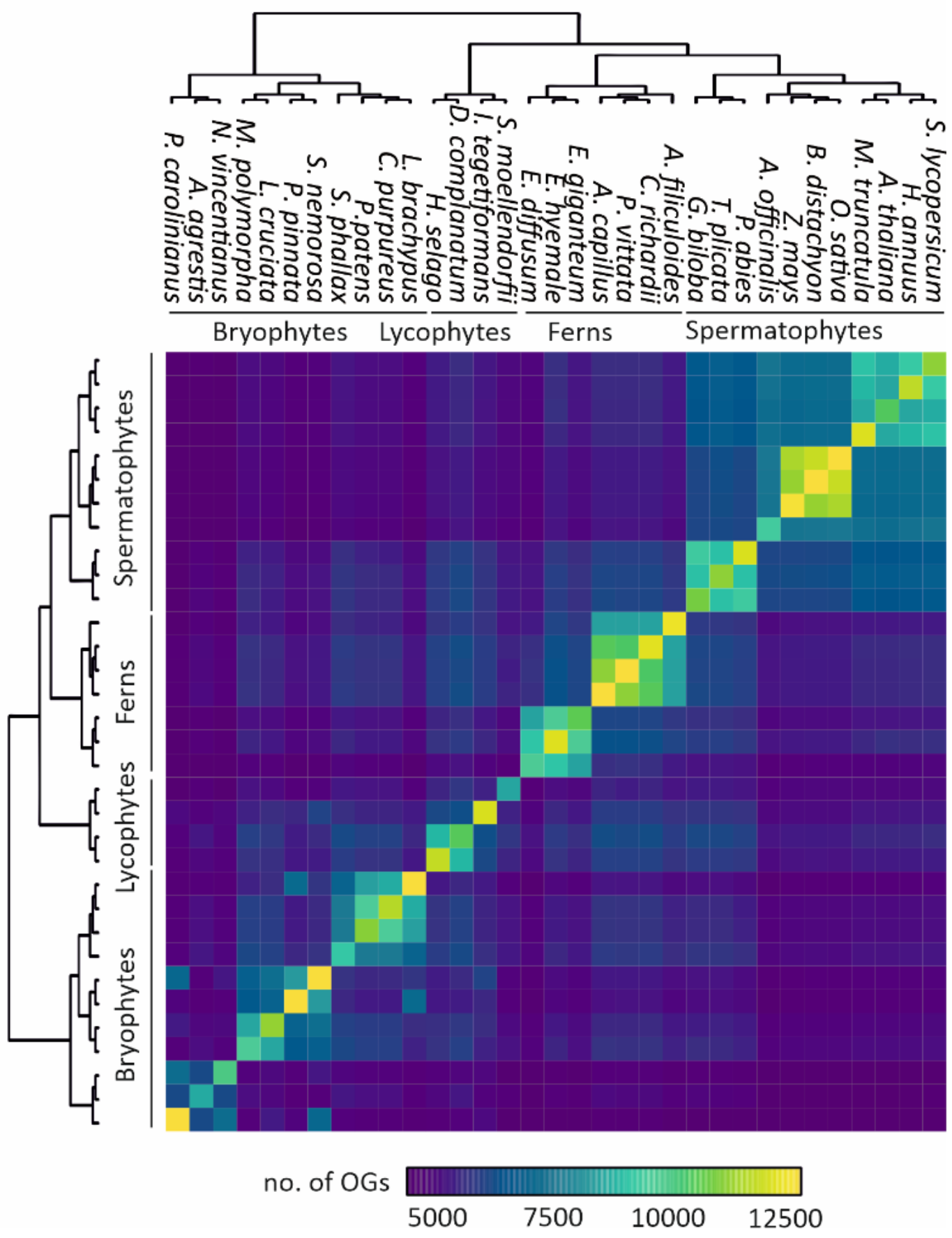

Supplemental Figure S2. **Classification of orthogroups across land plant evolution**. **A**) Bar plots depicting the number of conserved, gained, lost and lineage specific orthogroups in *M. polymorpha*, *P. patens*, *S. moellendorfii*, *E. hyemale*, *P. vittata*, *O. sativa* and *A. thaliana*. **B**) The amount of orthogroups shared between pairs of species.

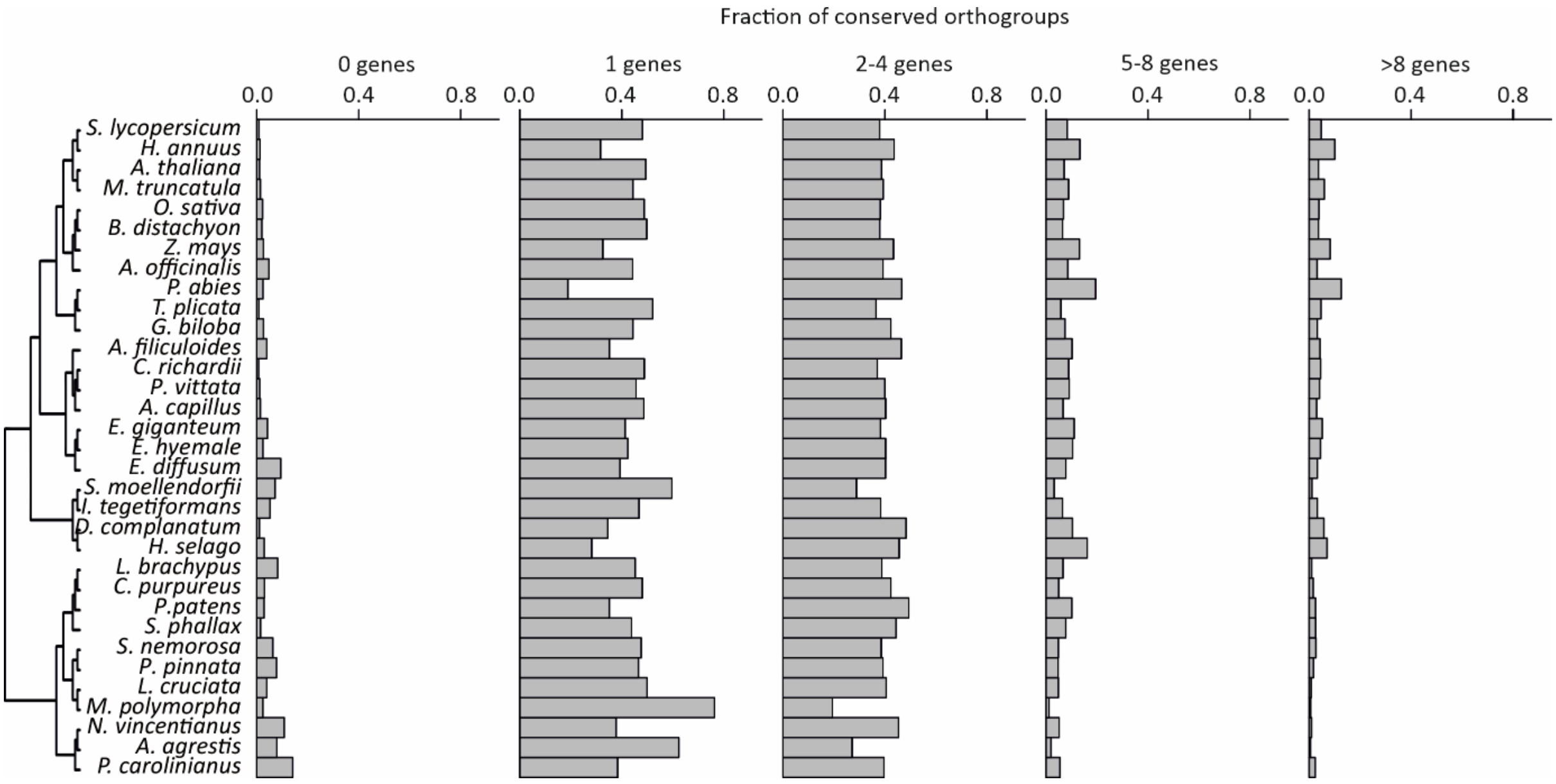

Supplemental Figure S3. Distribution of number of genes in conserved orthogroups. Percentage of number of genes, over the total number of genes, included in each of the fraction of conserved orthogroups identified for the 33 land plant species analysed in this study. Fractions of conserved orthogroups are classified according to the number of genes they contain (0, 1, 2-4, 5-8 and more than 8 genes).

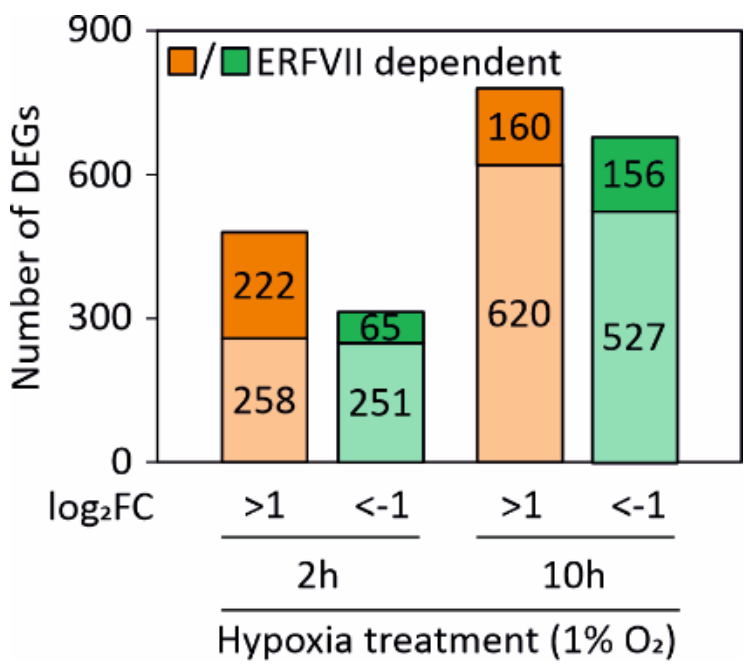

Supplemental Figure S4. **Contribution of the ERFVIIs to the hypoxia response in *A. thaliana***. Bar plots representing the number of differentially expressed genes (DEGs) in *A. thaliana* following two or ten hours of dark hypoxia treatment (1% v/v O_2) in 7-day-old seedlings of WT (Col-0) plants compared to the *erfVII* mutant. Light orange and light green represent upregulated ($log_2FC>1$, FDR<0.05) or downregulated ($log_2FC<-1$, FDR<0.05) genes, respectively. Dark orange and dark green represent ERFVII-dependent upregulated ($log_2FC>1$, FDR<0.05) or downregulated ($log_2FC<-1$, FDR<0.05) genes, respectively. The exact number of genes for each category is highlighted inside each bar.

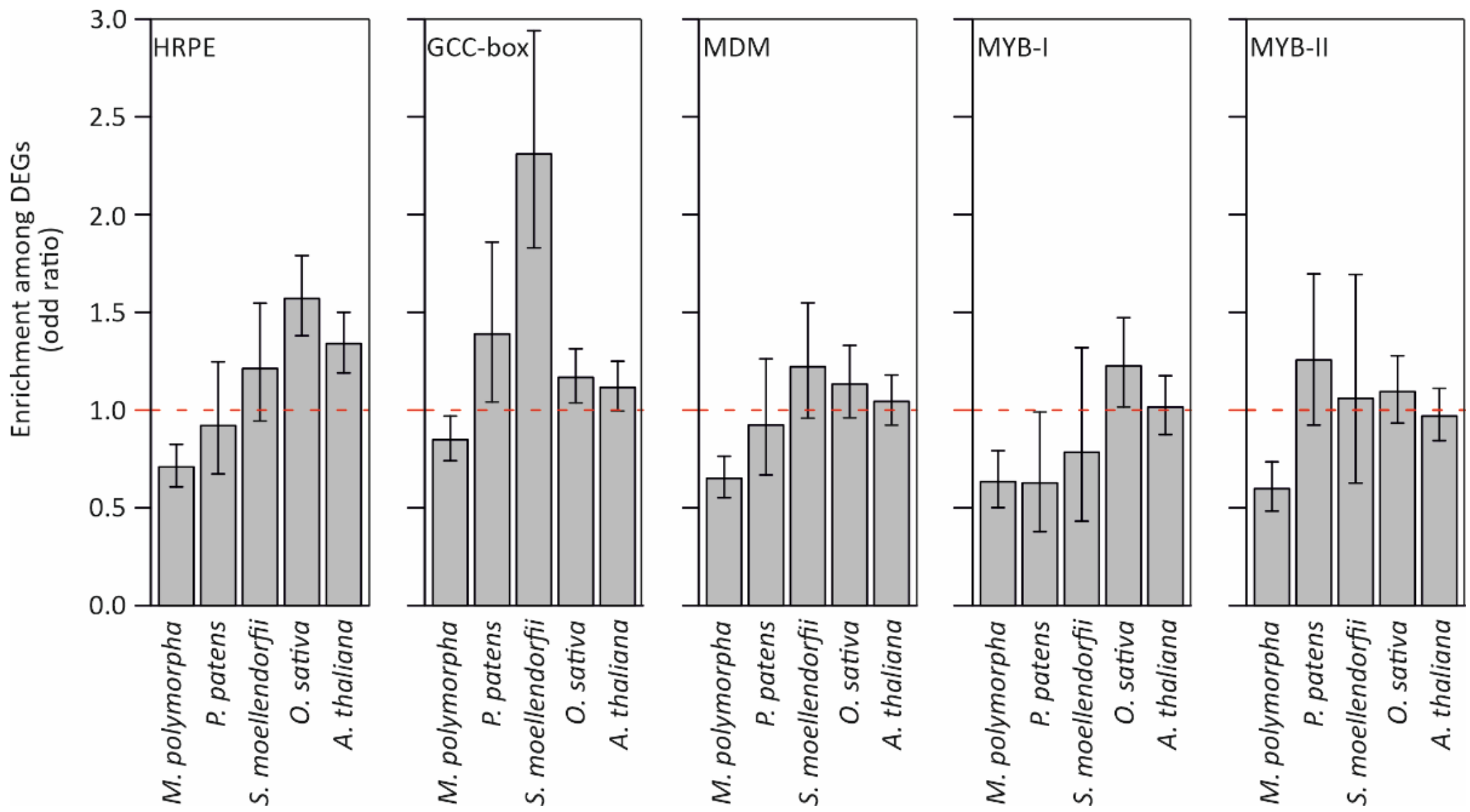

Supplemental Figure S5. **Motifs enrichment upon hypoxia in land plants**. Enrichment of *cis*-element motifs (HRPE, GCC-box, MDM, MYB-I and MYB-II) among differentially upregulated genes (P<0.05 and $|log_2FC|>1$). Odds ratio of 1 indicates no enrichment, whereas higher values are enriched. Error bars represent the 95% confidence intervals (Fisher's exact test).

A

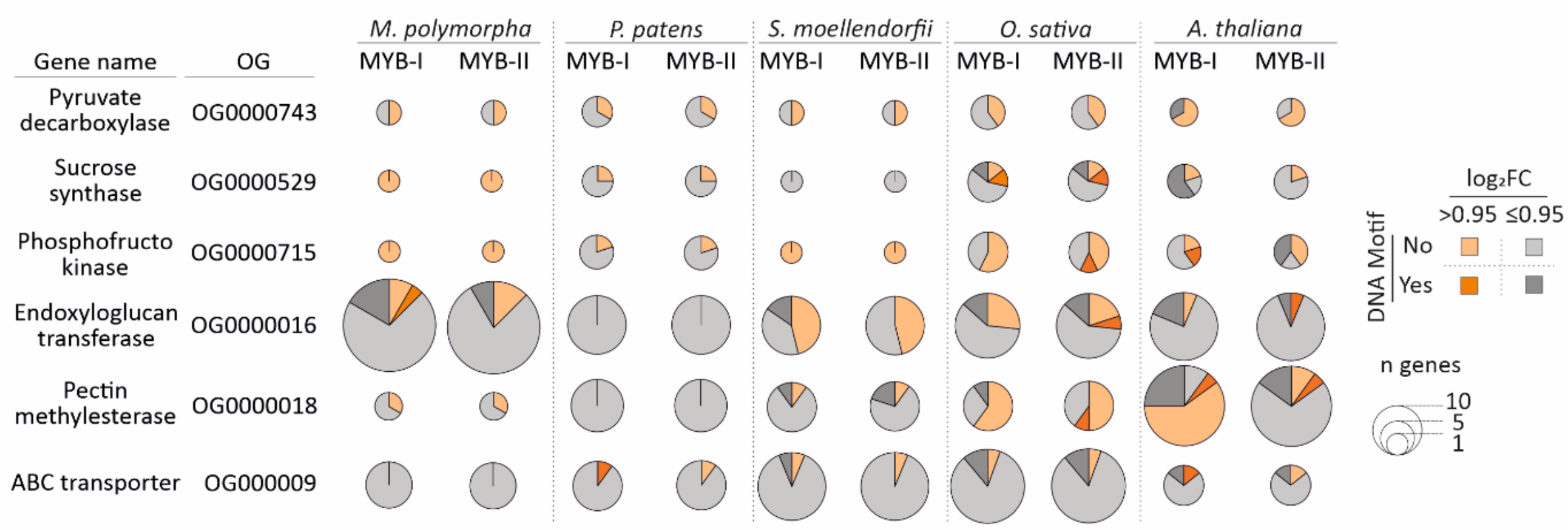

B

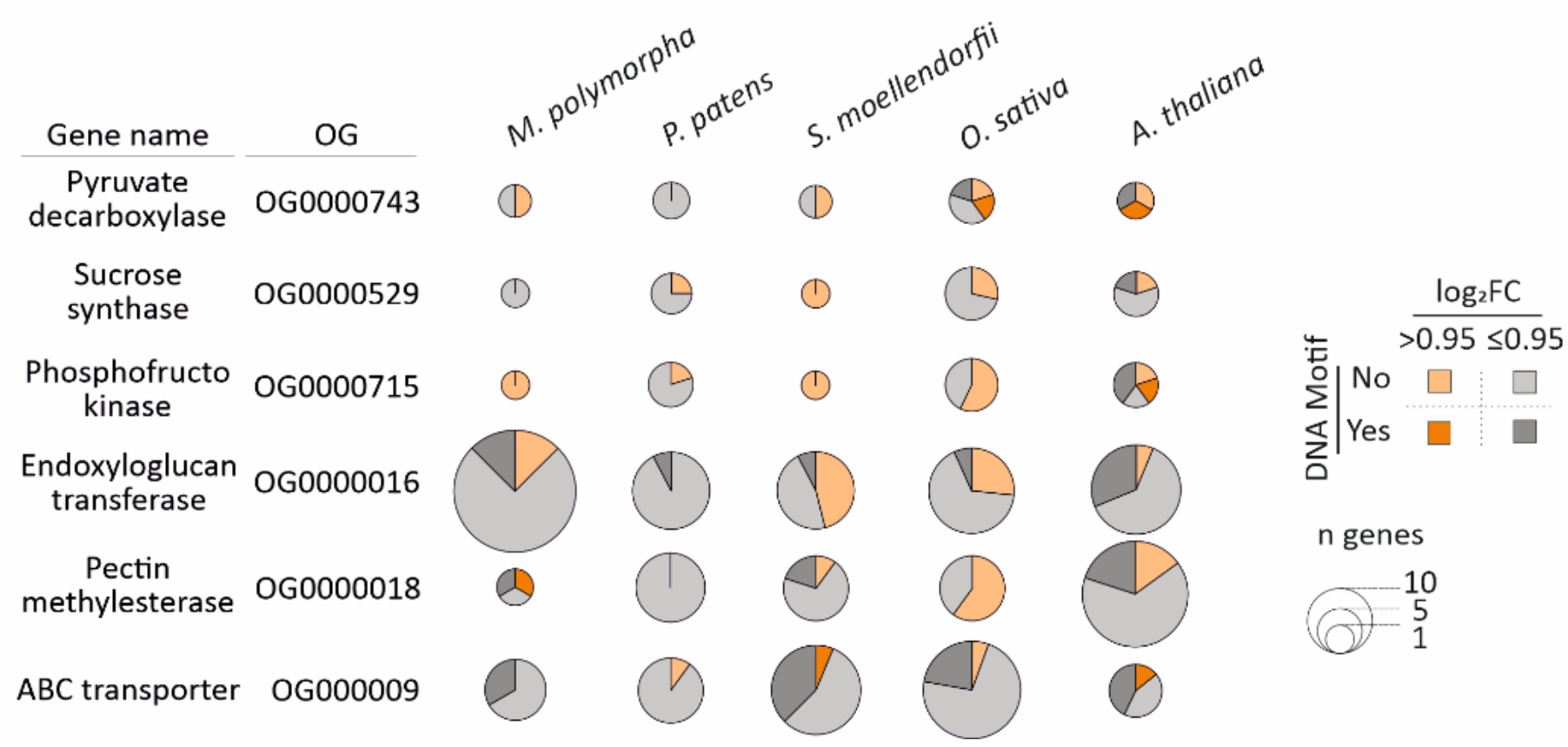

Supplemental Figure S6. **Frequency of MYB and NAC *cis*-element motifs in hypoxia-induced promoters across land plants**. **A**) Pie charts representing the occurrence of MYB-binding motifs (MYB-I and MYB-II) or **B**) NAC-binding motif (MDM) in promoters of upregulated (dark orange, $log_2FC>0.95$) and downregulated (dark grey, $log_2FC\leq0.95$) genes belonging to the seven hypoxia-induced and conserved orthogroups in *M. polymorpha*, *P. patens*, *S. moellendorfii*, *E. hyemale*, *P. vittata*, *O. sativa* and *A. thaliana*. The size of each pie chart is proportional to the number of genes belonging to the orthogroups for each individual species, with total upregulated genes in light orange ($log_2FC>0.95$) and total downregulated genes in light grey ($log_2FC\leq0.95$).

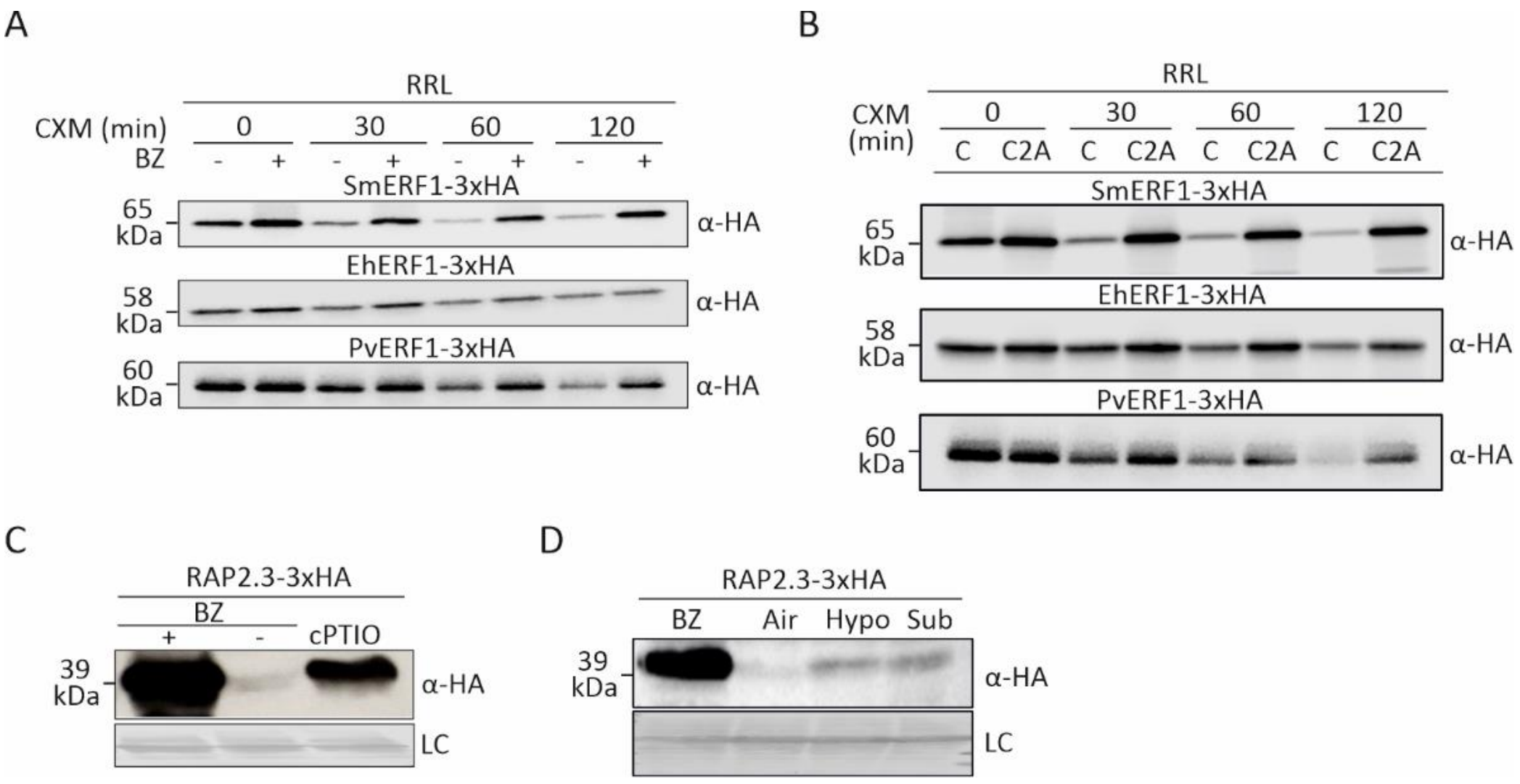

Supplemental Figure S7. **Tracheophyte ERFVIIs regulation through the PCO branch of the PRT6 N-degron pathway**. **A**) Western blot analysis of $SmERF1^{3xHA}$, $EhERF1^{3xHA}$ and $PvERF1^{3xHA}$ abundance in rabbit reticulocyte lysate (RRL) in control (DMSO) or bortezomib (BZ) conditions during a time course (0, 30, 60 and 120 min) after cycloheximide (CXM) addition. **B**) Western blot analysis of Cys2 compared to C2A $SmERF1^{3xHA}$, $EhERF1^{3xHA}$ and $PvERF1^{3xHA}$ abundance in RRL during a time course (0, 30, 60 and 120 min) after cycloheximide (CXM) treatment. **C**) Western blot analysis of $RAP2.3^{3xHA}$ abundance in control (DMSO), bortezomib (BZ) or NO-scavenger (cPTIO) treated *erfVII* mutant seedlings. **D**) Western blot analysis of $RAP2.3^{3xHA}$ abundance in bortezomib (BZ), air (21% v/v O_2), hypoxia (2h, 0.1% v/v O_2) or submergence (1h) treated *erfVII* mutant seedlings. Loading control (LC) corresponds to a re-fitted and compacted image of the membrane after Ponceau staining. The unedited images are shown in **Supplemental Fig. S16**.

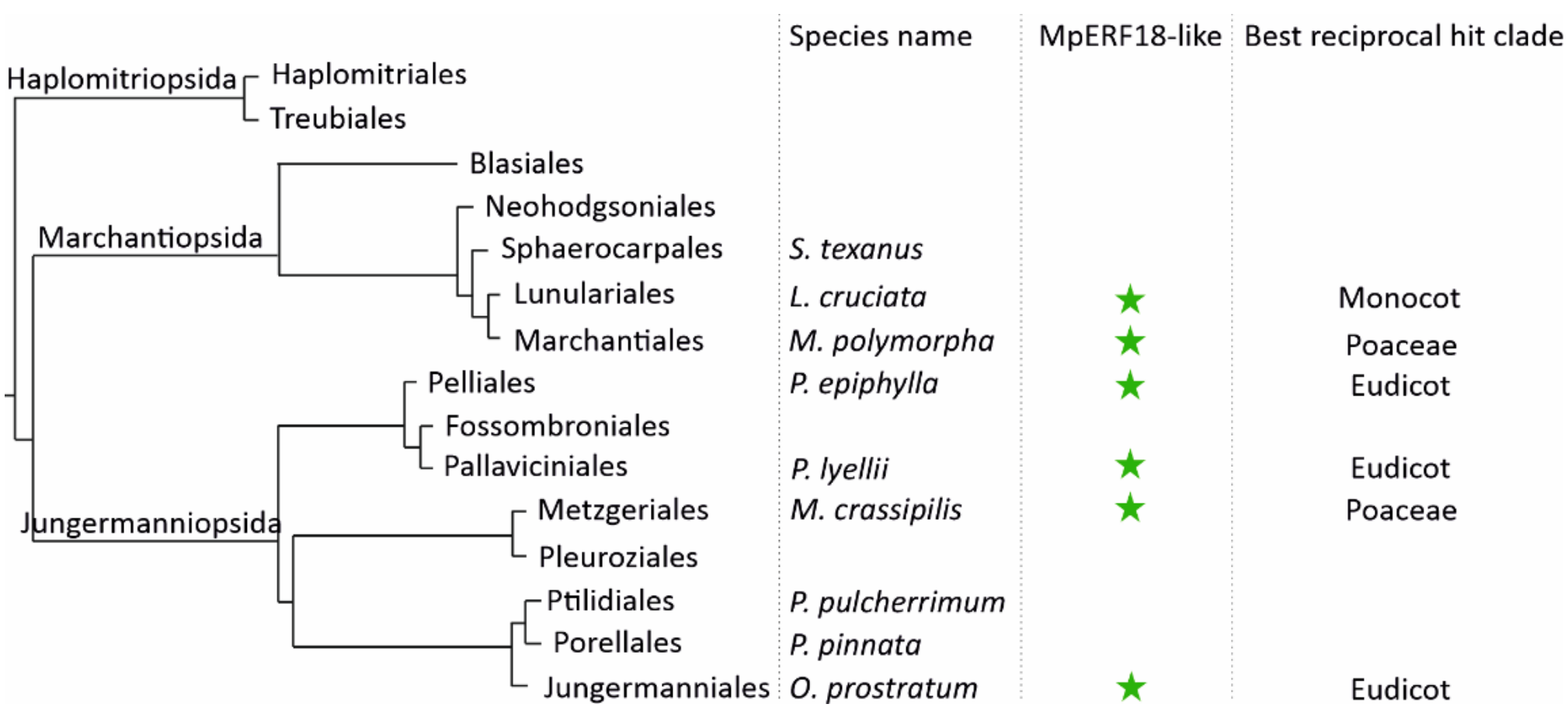

Supplemental Figure S8. **Occurrence of *M. polymorpha* MpERF18-like proteins in liverworts**. tblastn algorithm search of the 1KP database using *M. polymorpha* MpERF18 (Mapoly0092s0058) protein sequence as query. The presence of orthologues of MpERF18 are marked with a green star, together with the species name where they have been identified. For each MpERF18 orthologs found, their best reciprocal hit clade is as well reported.

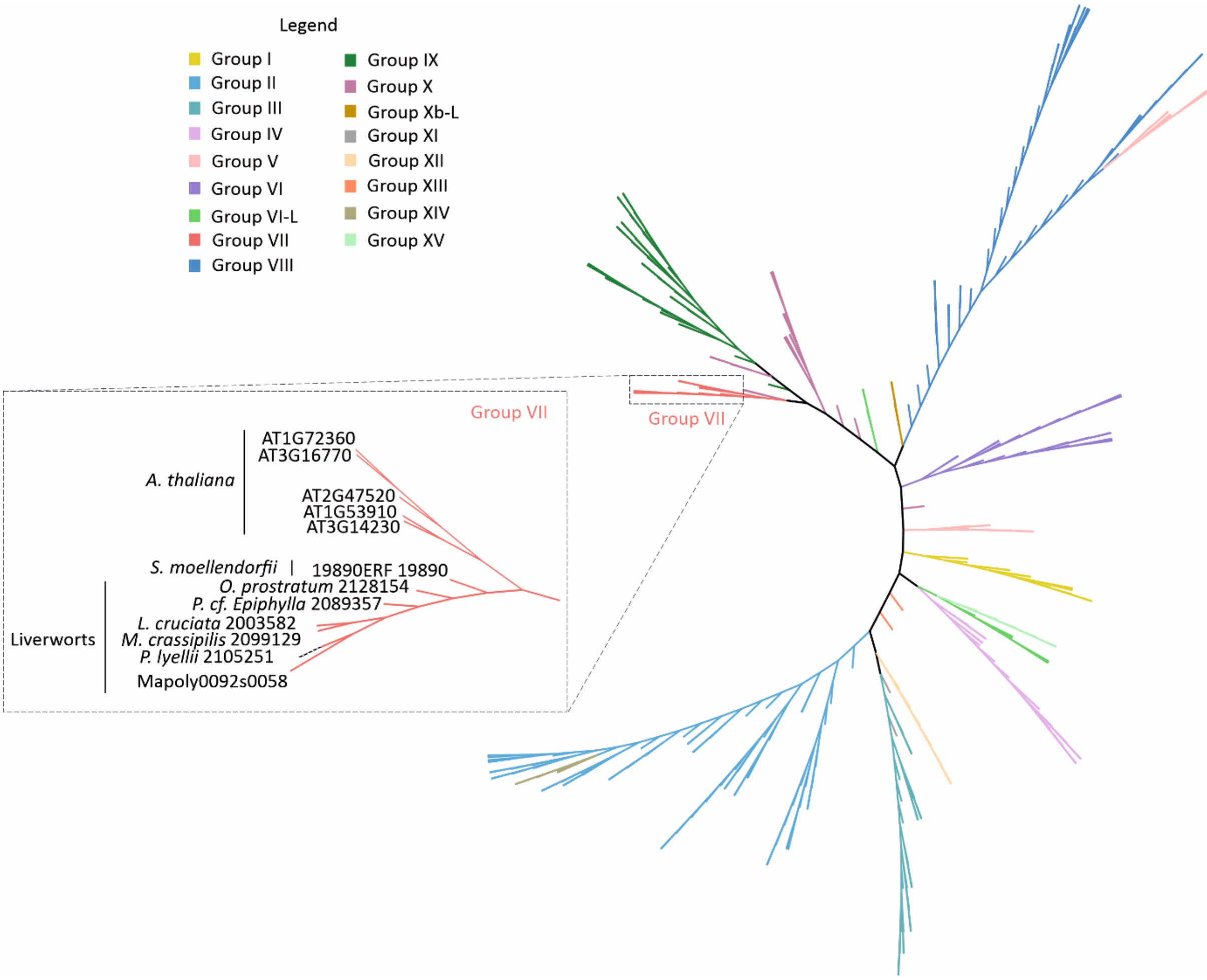

Supplemental Figure S9. **Phylogenetic relationship between ERF proteins in bryophytes, *S. moellendorfii* and *A. thaliana*.** Unrooted phylogenetic tree of ERF proteins from bryophytes (*A. punctatus*, *M. polymorpha* and *P. patens*), lycophyte (*S. moellendorfii*) and angiosperm (*A. thaliana*), including orthologous ERFVII-like proteins of MpERF18 retrieved from other groups of liverworts, which are shown in **Supplemental Fig. S8** and listed in **Supplemental Table 34**. ERFs classification is based on (Nakano et al., 2006). In the dashed box is a magnification of the Group VII branch, where the names of the sequences at each node and the species or clade to which they belong are highlighted.

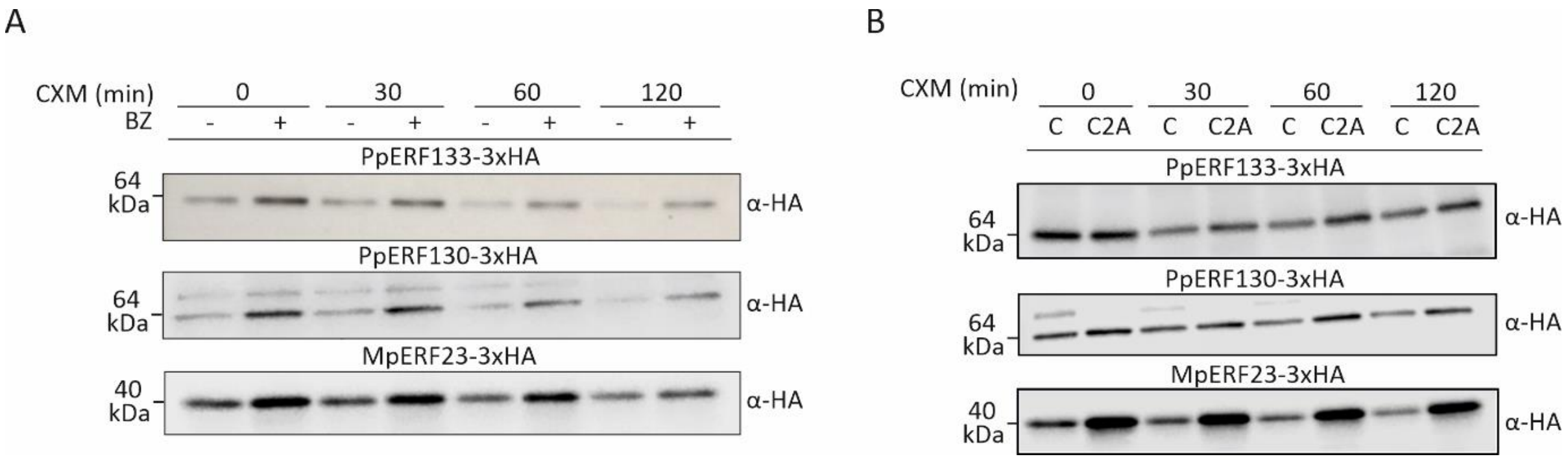

Supplemental Figure S10. **Bryophyte ERF regulation through the N-degron pathway. A**) Western blot analysis of ERF Cys2 proteins PpERF133^{3xHA}, PpERF130^{3xHA} and MpERF23^{3xHA} abundance in rabbit reticulocyte lysate (RRL) in control (DMSO) or bortezomib (BZ) conditions during a time course (0, 30, 60 and 120 min) after cycloheximide (CXM) treatment. **B**) Western blot analysis of Cys2 compared to C2A PpERF133^{3xHA}, PpERF130^{3xHA} and MpERF23^{3xHA} abundance in RRL during a time course (0, 30, 60 and 120 min) after cycloheximide (CXM) treatment.

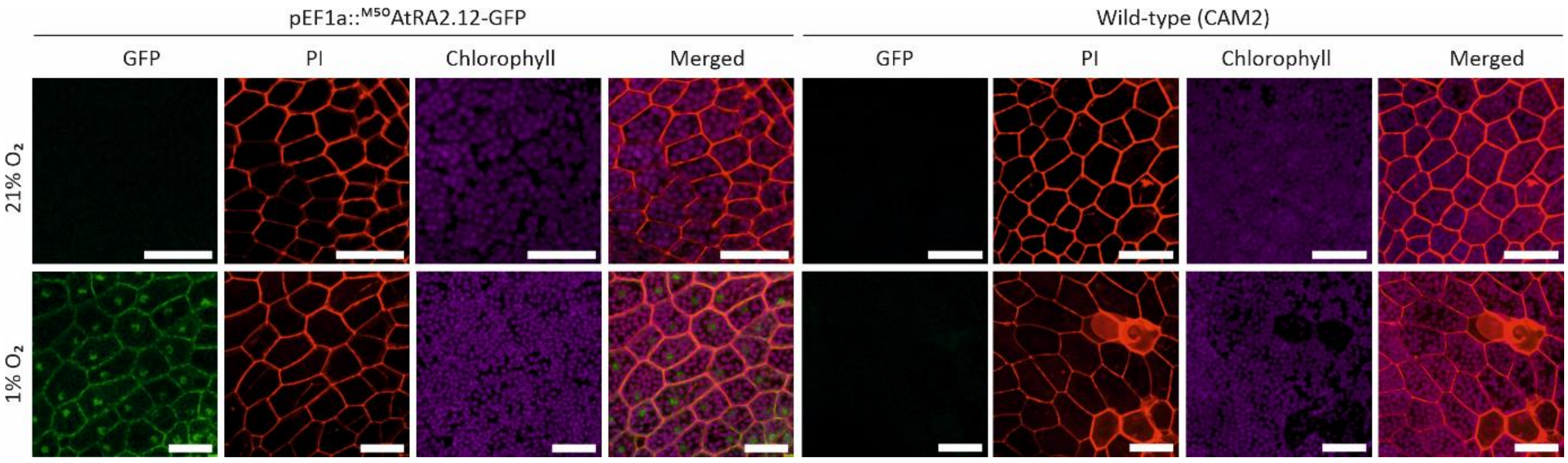

Supplemental Figure S11. ***A. thaliana* RAP2.12 stability depends on hypoxia in *M. polymorpha***. Confocal images of the subcellular localization of a reporter consisting of the first 50 aa of AtRAP2.12 fused to Citrine, overexpressed in *M. polymorpha* 13-day-old thalli in air (21% ambient O_2) and hypoxia (1% v/v O_2), compared to wild-type (CAM2) plants.

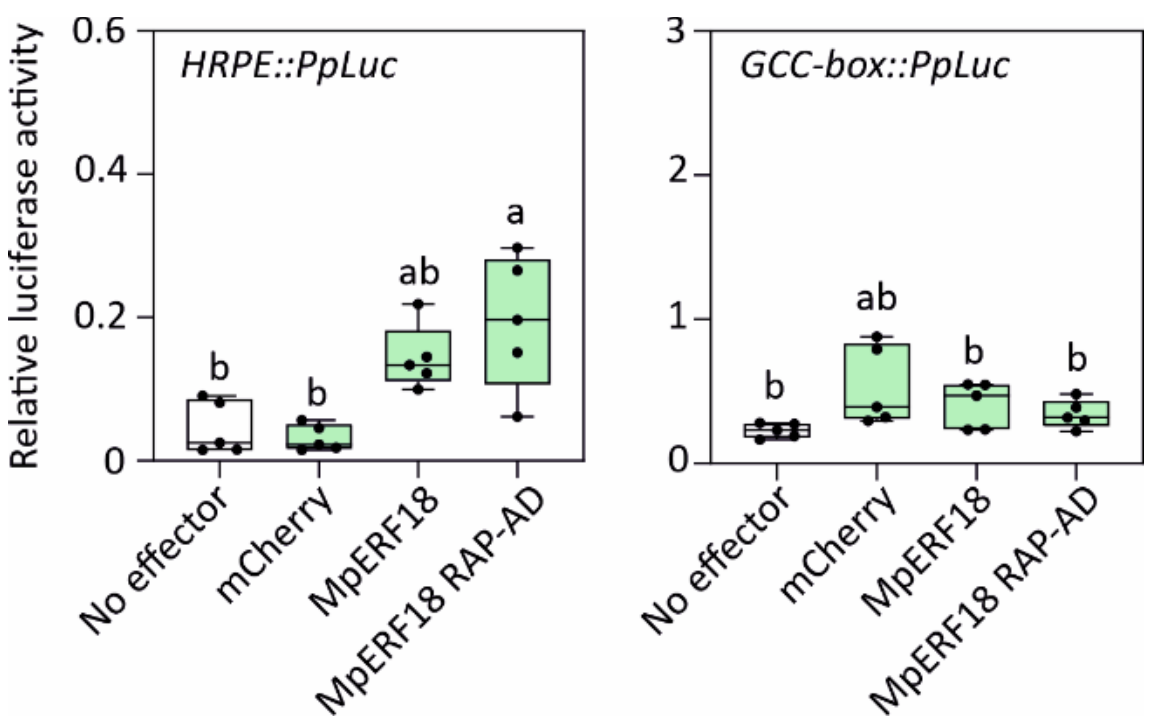

Supplemental Figure S12. **MpERF18 transactivation efficiency on hypoxia-related promoters**. Effect of *M. polymorpha* ERFVII-like (MpERF18) wild type or fused at the C-terminal to RAP2.12 activation domain (MpERF18 RAP-AD) on the synthetic promoters HRPE (left) and GCC-box (right), measured as relative firefly luciferase activity in *A. thaliana* wild-type mesophyll protoplasts. Data are presented as means ± SD (n=5). Different letters indicate statistically different averages as assessed by one-way ANOVA followed by the Tukey's posthoc test ($P < 0.05$).

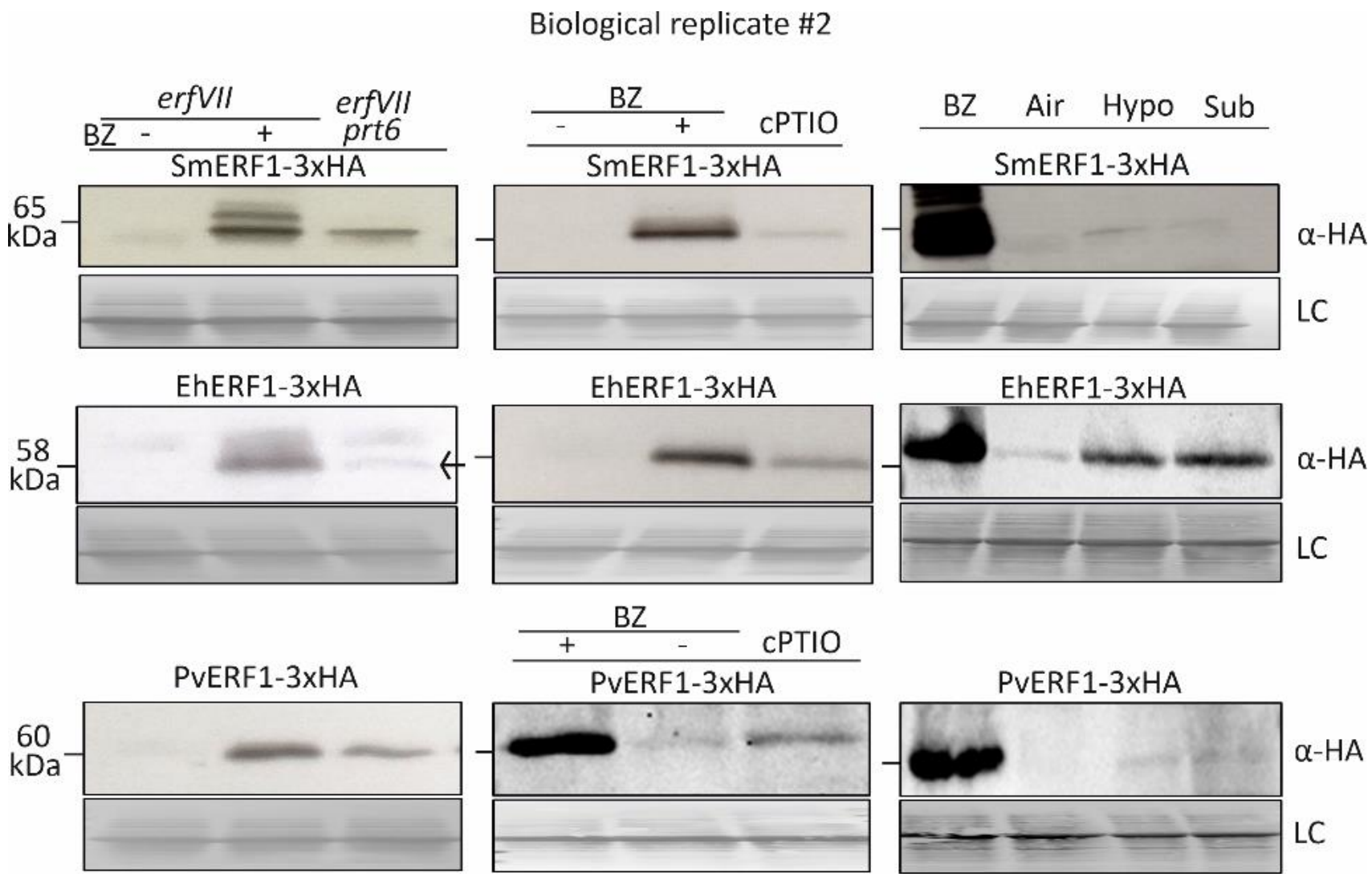

Supplemental Figure S13. Western blot analysis of SmERF1^{3xHA}, EhERF1^{3xHA} and PvERF1^{3xHA} abundance in control (DMSO), bortezomib (BZ), NO-scavenger (cPTIO), dark air (2h), dark hypoxia (2h, 0.1% v/v O_2) or dark submergence (1h) treated *erfVII* or *erfVII prt6* mutant 7-day-old whole seedlings (second biological replicate of what is shown in Fig. 3D. Loading control (LC) corresponds to a re-fitted and compacted image of the membrane after Ponceau staining. The unedited images are shown in **Supplemental Fig. S17-18**.

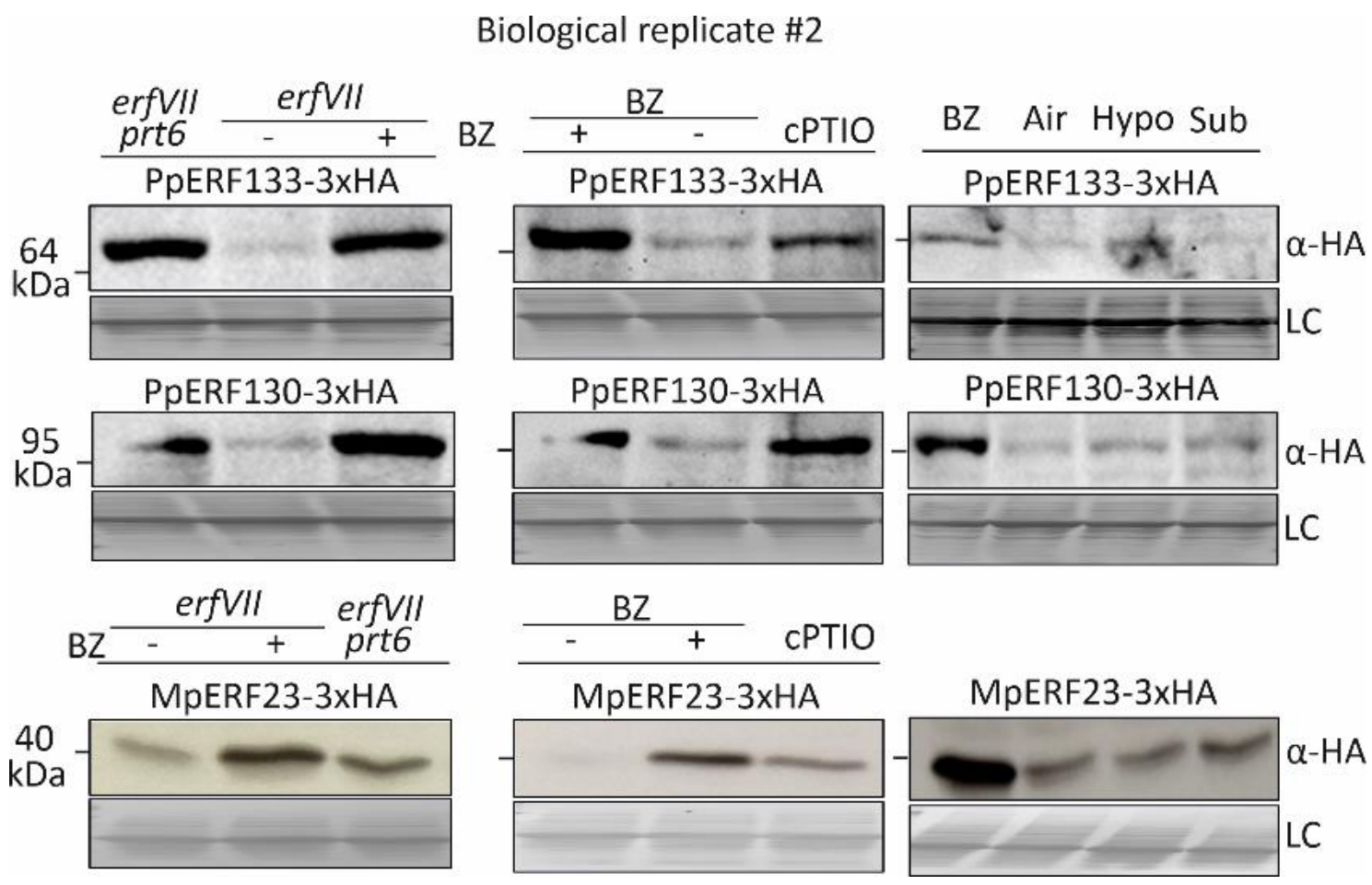

Supplemental Figure S14. Western blot analysis of PpERF133^{3xHA}, PpERF130^{3xHA} and MpERF23^{3xHA} abundance in control (DMSO), bortezomib (BZ), NO-scavenger (cPTIO), dark air (2h), dark hypoxia (2h, 0.1% v/v O_2) or dark submergence (1h) treated *erfVII* or *erfVII prt6* mutant 7-day-old whole seedlings (second biological replicate of what is shown in Fig. 4C). The black arrow indicates the expected protein size band. Loading control (LC) corresponds to a re-fitted and compacted image of the membrane after Ponceau staining. The unedited images are shown in **Supplemental Fig. S17-18**.

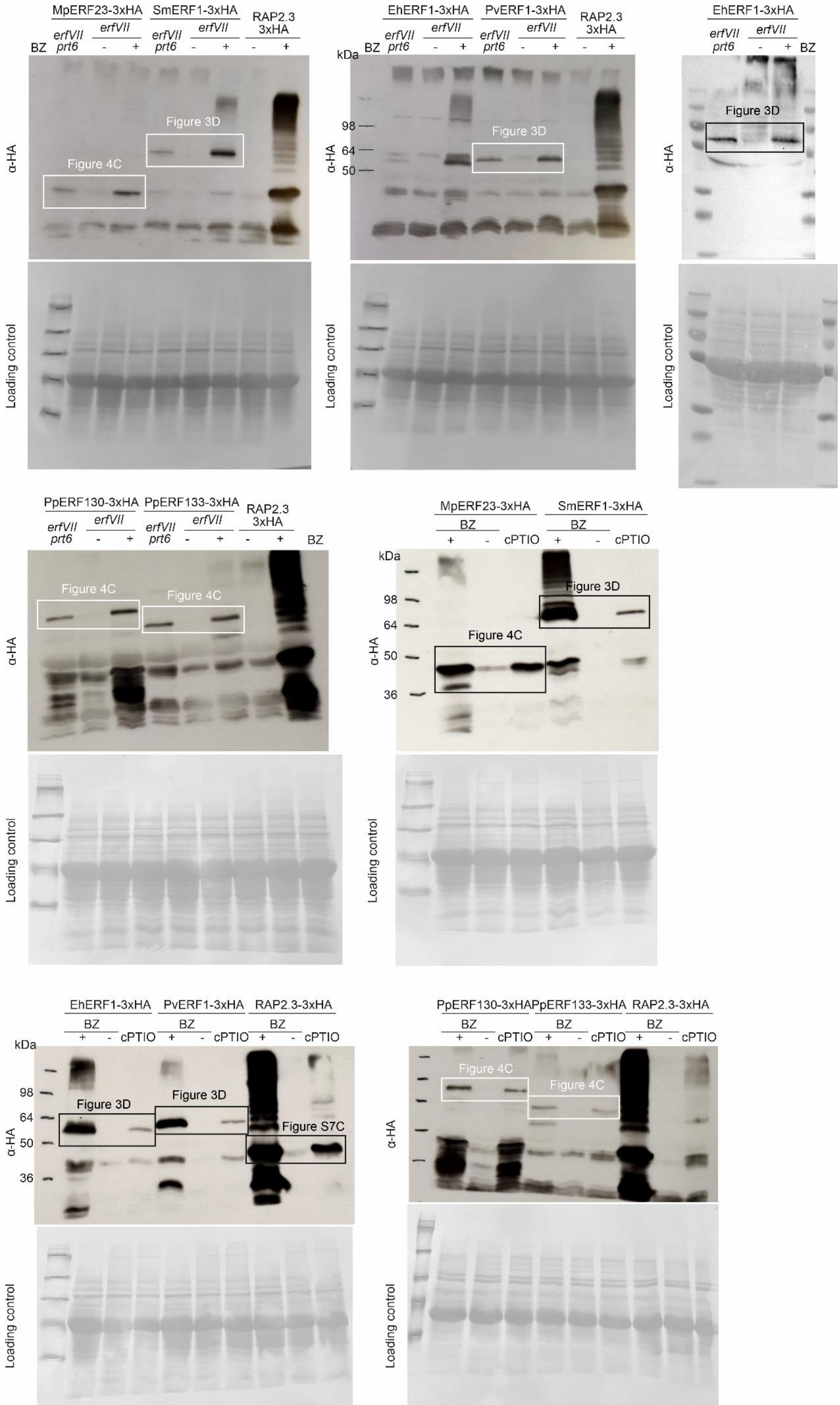
MpERF23-3xHA
SmERF1-3xHA
RAP2.3 3xHA
erfVII prt6
erfVII
BZ
Figure 3D
Figure 4C
α-HA
Loading control
EhERF1-3xHA
PvERF1-3xHA
kDa
98
64
50
PpERF130-3xHA
PpERF133-3xHA
cPTIO
36
RAP2.3-3xHA
Figure S7C

Supplemental Figure S15. Collection of original Western blots, and corresponding Ponceau staining, shown in Fig. 3D, 4C and S7C, related to cPTIO treatment and *erfVII prt6* background of various Cys2-ERFs.

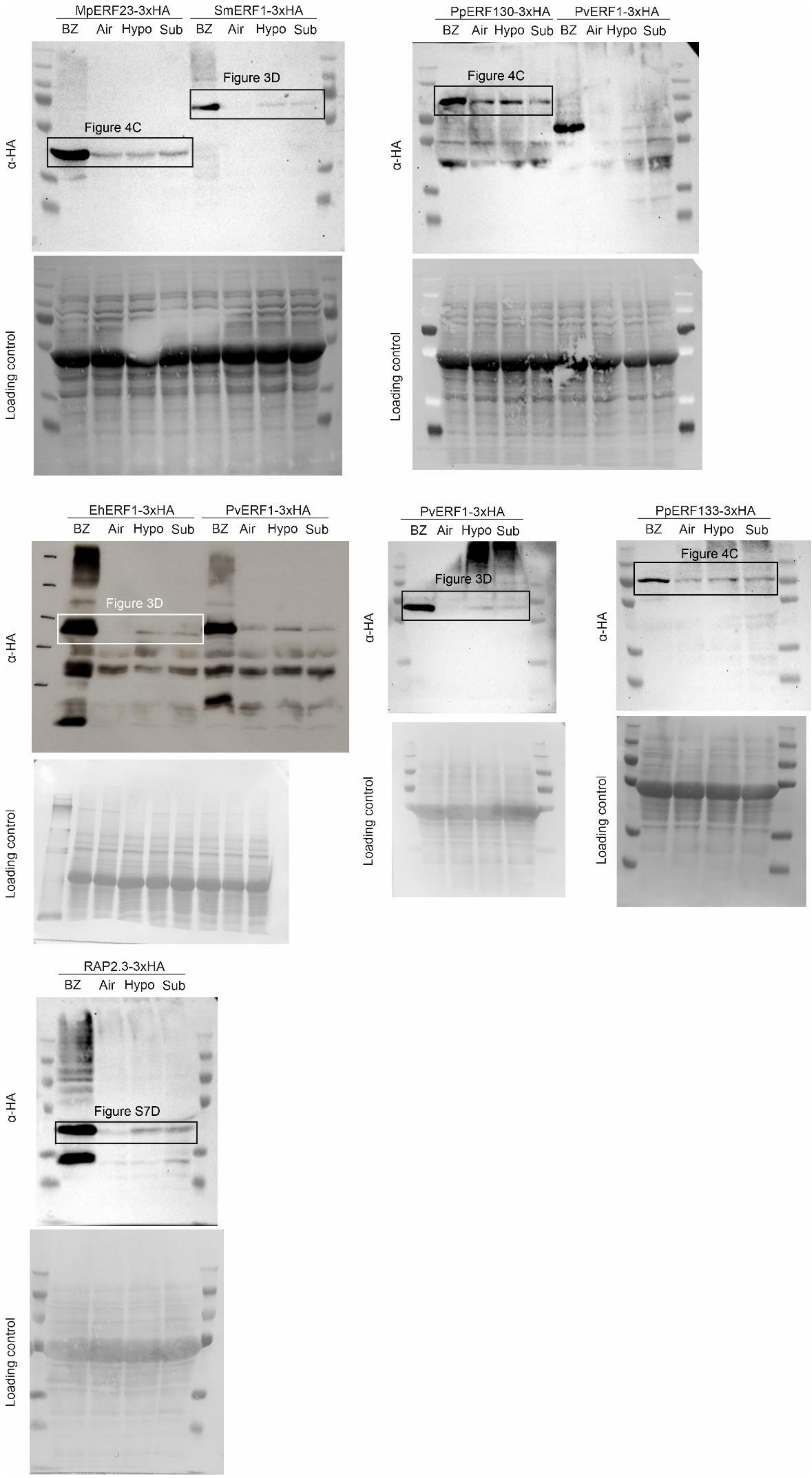
MpERF23-3xHA
SmERF1-3xHA
BZ Air Hypo Sub BZ Air Hypo Sub
Figure 3D
Figure 4C
α-HA
Loading control
PpERF130-3xHA
PvERF1-3xHA
BZ Air Hypo Sub BZ Air Hypo Sub
Figure 4C
α-HA
Loading control
EhERF1-3xHA
PvERF1-3xHA
BZ Air Hypo Sub BZ Air Hypo Sub
Figure 3D
α-HA
Loading control
PvERF1-3xHA
BZ Air Hypo Sub
Figure 3D
α-HA
Loading control
PpERF133-3xHA
BZ Air Hypo Sub
Figure 4C
α-HA
Loading control
RAP2.3-3xHA
BZ Air Hypo Sub
Figure S7D
α-HA
Loading control

Supplemental Figure S16. Collection of original Western blots, and corresponding Ponceau staining, shown in Fig. 3D, 4C and S7D, related to hypoxia and submergence treatments of various Cys2-ERFs.

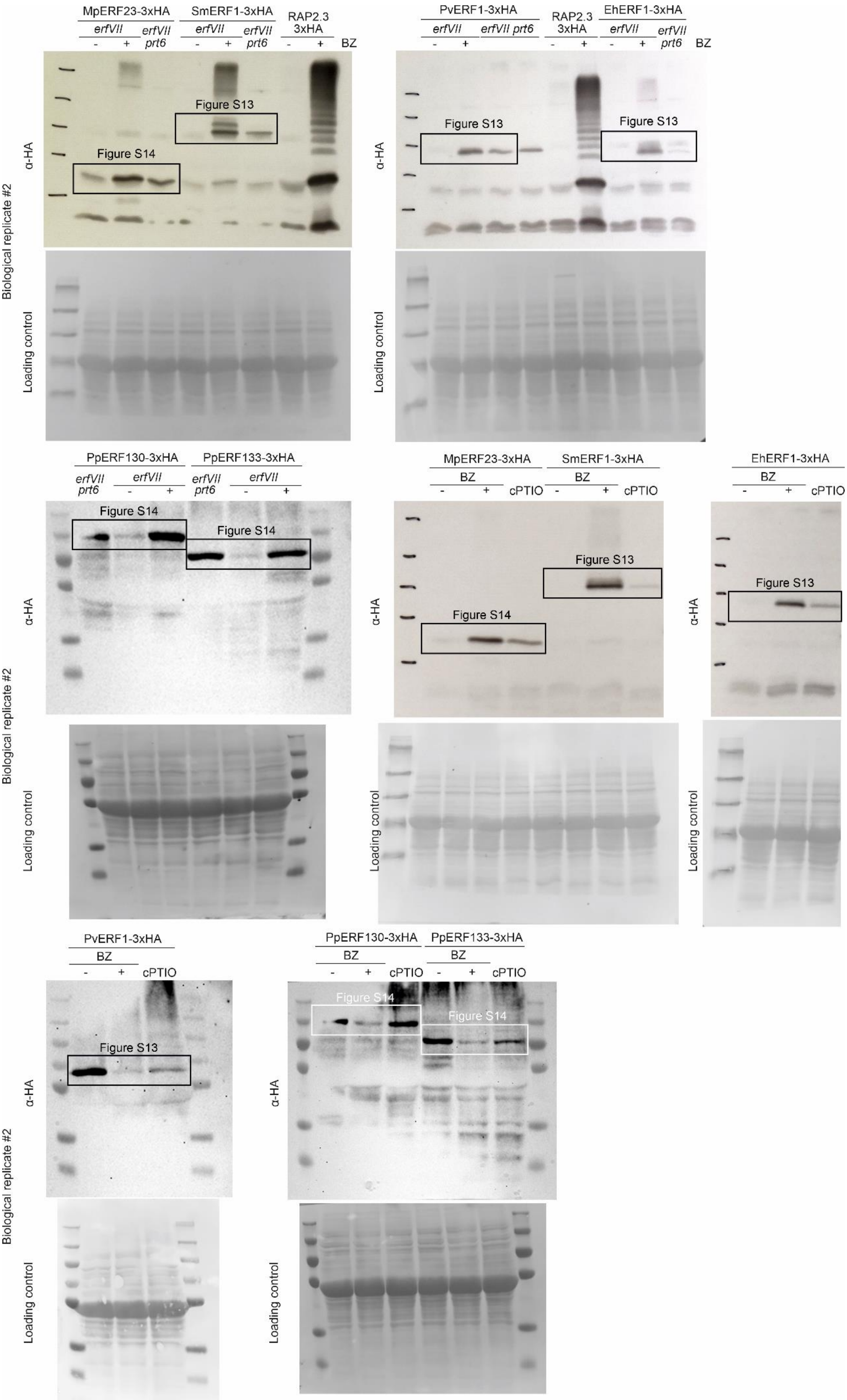

MpERF23-3xHA
SmERF1-3xHA
RAP2.3 3xHA
erfVII
erfVII prt6
BZ
Figure S13
Figure S14
α-HA
Loading control
Biological replicate #2
PvERF1-3xHA
EhERF1-3xHA
erfVII prt6
PpERF130-3xHA
PpERF133-3xHA
cPTIO

Supplemental Figure S17. Collection of original Western blots, and corresponding Ponceau staining, shown in Supplemental Fig. S13 and S14, related to cPTIO treatment and *erfVII prt6* background of various Cys2-ERFs.

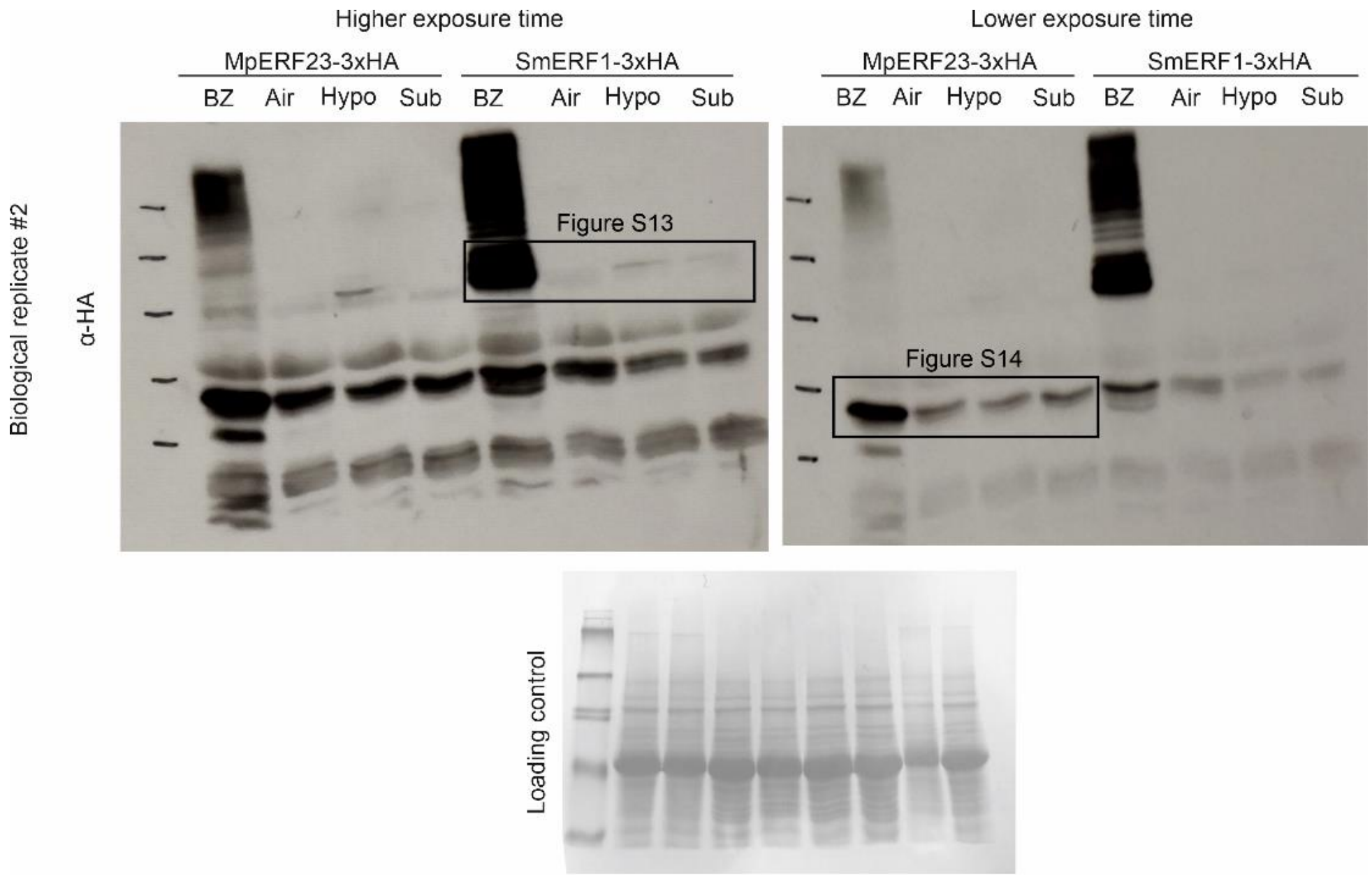
Higher exposure time
Lower exposure time
MpERF23-3xHA
SmERF1-3xHA
BZ Air Hypo Sub
Biological replicate #2
α-HA
Figure S13
Figure S14
Loading control

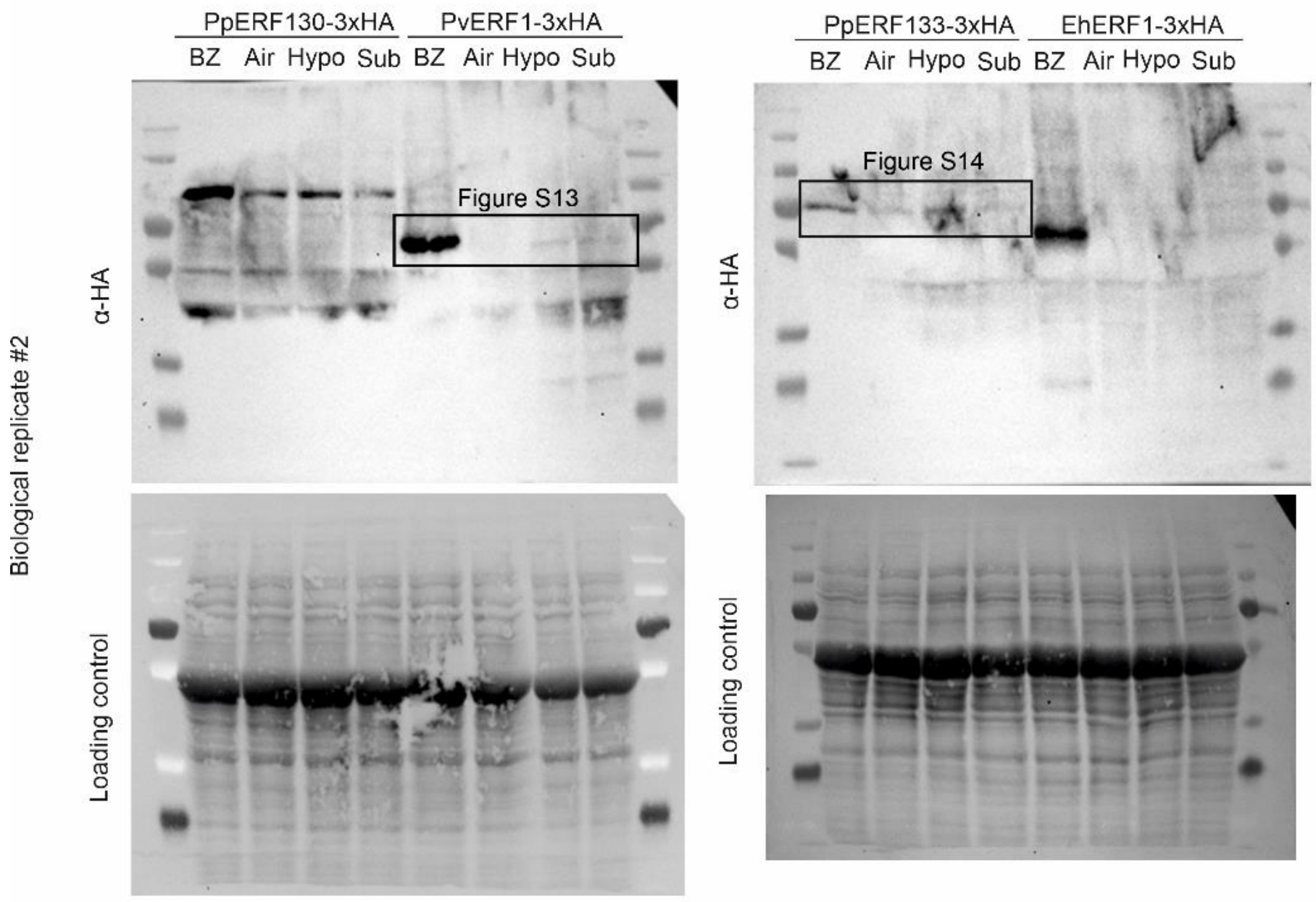
PpERF130-3xHA
PvERF1-3xHA
PpERF133-3xHA
EhERF1-3xHA
BZ Air Hypo Sub
Biological replicate #2
α-HA
Figure S13
Figure S14
Loading control

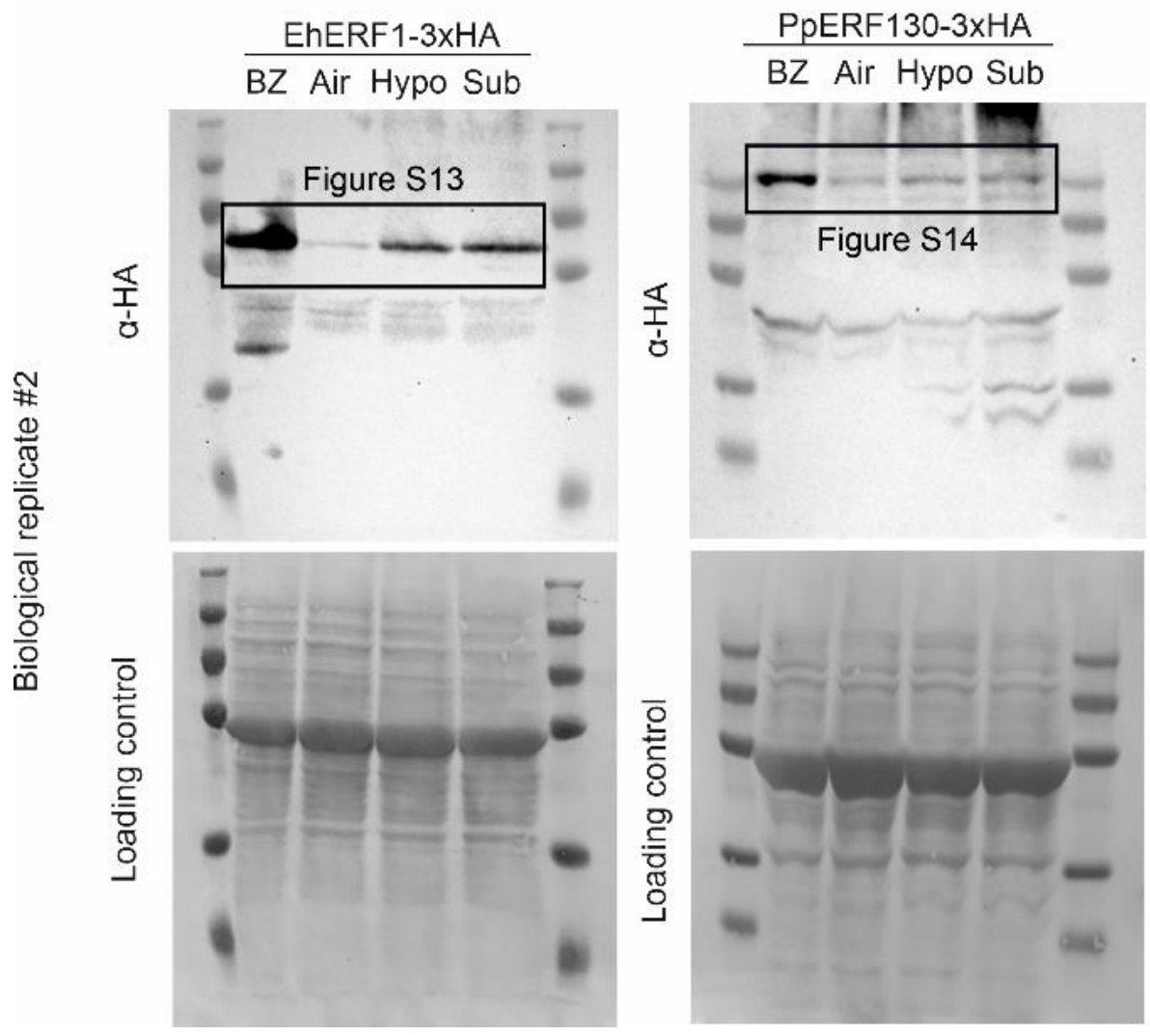
EhERF1-3xHA
PpERF130-3xHA
BZ Air Hypo Sub
Biological replicate #2
α-HA
Figure S13
Figure S14
Loading control

Supplemental Figure S18. Collection of original Western blots, and corresponding Ponceau staining, shown in Supplemental Fig. S13 and S14, related to hypoxia and submergence treatments of various Cys2-ERFs.

Supplemental Tables

Supplemental Table 1. RNAseq analysis of *Marchantia polymorpha* in control (21% v/v O_2) and hypoxia (1% v/v O_2), for eight hours in the dark.
Supplemental Table 2. RNAseq analysis of *Physcomitrium patens* in control (21% v/v O_2) and hypoxia (1% v/v O_2), for eight hours in the dark.
Supplemental Table 3. RNAseq analysis of *Selaginella moellendorfii* in control (21% v/v O_2) and hypoxia (1% v/v O_2), for eight hours in the dark.
Supplemental Table 4. RNAseq analysis of *Equisetum hyemale* in control (21% v/v O_2) and hypoxia (1% v/v O_2), for eight hours in the dark.
Supplemental Table 5. RNAseq analysis of *Pteris vittata* in control (21% v/v O_2) and hypoxia (1% v/v O_2), for eight hours in the dark.
Supplemental Table 6. List of *M. polymorpha* genes associated to each orthogroup defined according the OrthoFinder method (Emms and Kelly 2015), alongside with the corresponding log_2FC and fold discovery rate (FDR), in hypoxia.
Supplemental Table 7. List of *P. patens* genes associated to each orthogroup defined according the OrthoFinder method (Emms and Kelly 2015), alongside with the corresponding log_2FC and fold discovery rate (FDR), in hypoxia.
Supplemental Table 8. List of *S. moellendorfii* genes associated to each orthogroup defined according the OrthoFinder method (Emms and Kelly 2015), alongside with the corresponding log_2FC and fold discovery rate (FDR), in hypoxia.
Supplemental Table 9. List of *E. hyemale* genes associated to each orthogroup defined according the OrthoFinder method (Emms and Kelly 2015), alongside with the corresponding log_2FC and fold discovery rate (FDR), in hypoxia.
Supplemental Table 10. List of *P. vittata* genes associated to each orthogroup defined according the OrthoFinder method (Emms and Kelly 2015), alongside with the corresponding log_2FC and fold discovery rate (FDR), in hypoxia.
Supplemental Table 11. List of *O. sativa* genes associated to each orthogroup defined according the OrthoFinder method (Emms and Kelly 2015), alongside with the corresponding log_2FC and fold discovery rate (FDR), in hypoxia.
Supplemental Table 12. List of *A. thaliana* genes associated to each orthogroup defined according the OrthoFinder method (Emms and Kelly 2015), and the corresponding log_2FC, along with the fold discovery rate (FDR), in hypoxia.
Supplemental Table 13. Number of orthogroups identified for each land plant group analysed in this study. Orthogroups are classified in 'conserved', 'gained', 'lost' and 'lineage specific'.
Supplemental Table 14. Number of genes that constitute the hierarchical clustering of conserved orthogroups from 33 species of land plants, including those studied in this work.
Supplemental Table 15. Percentage of number of genes, over the total number of genes, included in each of the fraction of conserved orthogroups identified for the 33 land plant species analysed in this study.

Supplemental Table 16. Number of orthogroups containing exclusively upregulated (log2FC>1) or downregulated (log2FC<-1) genes or simultaneously up- and down- regulated (-1<log2FC>1) genes under hypoxia. Gene cutoff 0 defines the proportion of genes in an orthogroup that need to be differentially expressed for the OG to be considered regulated.

Species name	Log$_2$FC>1	-1<Log2FC>1	Log$_2$FC<-1
M. polymorpha	263	7	283
P. patens	28	1	65
S. moellendorfii	11	0	148
E. hyemale	92	25	375
P. vittata	86	7	639
O. sativa	908	56	464
A. thaliana	541	43	492

Supplemental Table 17. log$_2$FC<-1 (Padj. <0.05) of conserved hypoxia-regulated orthogroups in the land plant species considered in this study. Orthogroups are classified according to the molecular function of the orthologous *A. thaliana* gene.

Supplemental Table 18. log$_2$FC>1 (Padj. <0.05) of conserved hypoxia-regulated orthogroups in the land plant species considered in this study. Orthogroups are classified according to the molecular function of the orthologous *A. thaliana* gene.

Supplemental Table 19. List of hypoxia-induced and repressed conserved orthogroups in which the corresponding A. thaliana protein belongs to the 'enzyme' class.

Supplemental Table 20. RNAseq analysis of *Arabidopsis thaliana* wild-tpe (Col-0) and *erfVII* pentuple mutant after hypoxia treatment time course (2 h and 10 h of 1% v/v O2 in the dark).

Supplemental Table 21. HRPE motif enrichment analysis in promoters of orthogroups, divided by species.

Supplemental Table 22. GCC-box motif enrichment analysis in promoters of orthogroups, divided by species.

Supplemental Table 23. MDM motif enrichment analysis in promoters of orthogroups, divided by species.

Supplemental Table 24. MYB-I motif enrichment analysis in promoters of orthogroups, divided by species.

Supplemental Table 25. MYB-II motif enrichment analysis in promoters of orthogroups, divided by species.

Supplemental Table 26. HRPE motif enrichment on promoter regions of conserved hypoxia-induced orthogroups.

Supplemental Table 27. GCC-box motif enrichment on promoter regions of conserved hypoxia-induced orthogroups.

Supplemental Table 28. MDM motif enrichment on promoter regions of conserved hypoxia-induced orthogroups.

Supplemental Table 29. MYB-I motif enrichment on promoter regions of conserved hypoxia-induced orthogroups.

Supplemental Table 30. MYB-II motif enrichment on promoter regions of conserved hypoxia-induced orthogroups.

Supplemental Table 31. List or protein sequences from different species of lycophytes, eusporangiate ferns, leptosporangiate ferns, gymnosperms and angiosperms, orthologous of *Arabidopsis thaliana* RAP2.12, used to build the phylogenetic tree of ERFVII transcription factors family in tracheophytes.
Supplemental Table 32. List of ERF transcription factors from *Marchantia polymorpha*, *Physcomitrium patens*, *Anthoceros agrestis*, *Selaginella moellendorfii* and *A. thaliana* used to build the phylogenetic tree shown in **Fig. 4**.
Supplemental Table 33. List of ERF transcription factors from *Chlamydomonas reinhardtii* and *Klebsormidium flaccidum*.
Supplemental Table 34. List of the best *Marchantia polymorpha* ERFVII (Mapoly0092s0058, MpERF18) orthologs retrieved in specific classes and orders of liverworts.
Supplemental Table 35. List of ERF transcription factor proteins starting with a cysteine in second position in different species of bryophytes.
Supplemental Table 36. List of forward and reverse primer sequences (5’-3’) used for various cloning and RT-qPCR analysis in this study.

	Primer name	Gene name	Forward primer (5'-3')	Reverse primer (5'-3')
Cloning	PpERFVIII_SacII	*Pp3c7_1240V3.1*	gaaacccgcggtggttgttcacgagaagtagagaaa	ttttccgcggatattcatcggtaaagaaccc
	PpERFVIII_C2A		ccgcggtggtgcttcacgagaagta	tacttctcgtgaagcaccaccgcgg
	PpERFX_SacII	*Pp3c6_28370V3.1*	gaaacccgcggtggttgctttctgcaatcacttcca	ttttccgcggttcttgacacagagatgtgtc
	PpERFX_C2A		ccgcggtggtgcctttctgcaatca	tgattgcagaaaggcaccaccgcgg
	MpERFVII_SacII	*Mapoly0293s0001*	gaaacccgcggtggttgcagaatgaacaaaaggcta	ttttccgcggtcgaggtccatcgtatttgaa
	MpERFVI_C2A		cccgcggtggtgccagaatgaacaa	ttgttcattctggcaccaccgcggg
	SmERFVII_SacII	*73139*	gaaacccgcggtggttgccgtggtatcacctccgac	ttttccgcggccaagtaatctcttcgggatc
	SmERFVII_C2A		cccgcggtggtgcccgtggtatcac	gtgataccacgggcaccaccgcggg
	EhERF_SacII	*scaffold-JVSZ-2009360*	gaaacccgcggtggttgtggtggatccatcatttcc	ttttccgcgggtagtagtttgagaaatcttcaaag
	EhERF_C2A		cccgcggtggtgctggtggatccat	atggatccaccagcaccaccgcggg
	PvERF_SacII		gaaacccgcggtggttgcggcggatcgataatctcc	ttttccgcggctggccatcgtcaacagactt
	PvERF_C2A		cccgcggtggtgccggcggatcgat	atcgatccgccggcaccaccgcggg
	gbRAP2.12AD	*At1g53910*	gcgccgtctcgctcgttcggacaatggtgcaaaccctatg	gcgccgtctcgctcaaagctcagaagactcctccaatcat
RT-	sgUBQ	*At4g05320*	ggccttgtataatccctgatgaataag	aaagagataacaggaacggaaacatagt
	sgPDC1	*At4g33070*	cacagaatcttcaatgttcttacc	ccatgataaagcgtacatggaa

sgPCO2	*At5g39890*	cttcgagccgttttggatga	acgtcactaacggagatcgtcc
sgSmPDC	*157772*	aggtcgcctctcgtttggttg	agtgtcgttcttgcggagcttt
sgSmAHB	*80203*	gtctcacgcactcgtcgtct	acgacgtgcgtatgtcccat
sgSmADH	*145705*	acgcgattgagtgtgtcgga	ttgtgttgtcgtccaggcca
sgSm6PGD	*98399*	gctcatggatcccgagtttg	tccactcgctcgtaggtg
sgMpAct1	*Mapoly0016 s0137*	gagcgcggttactctttcac	gaccgtcaggaagctcgtag
sgMpPDC2	*Mapoly0160 s0004*	ggtcagcactccttacgctt	tagtcgttgaacacaggccc
sgMpETHE 1	*Mapoly0002 s0284*	tcatgccgggaggttctgga	cacggcaggcttgtcaggat
sgMpL2HG DH	*Mapoly0085 s0100*	ccacacgggaacgagagctg	ggtcggtgatctgcttcggg

Supplemental Table 37. Overview over Position-specific scoring matrices (PSSMs) for MYB-I and MYB-II *cis*-elements identified in this study. Matrices have been created using MEME version 5.5.7.

MYB I				MYB II			
a	c	g	t	a	c	g	t
0.5	0	0.17	0.33	0.25	0	0.75	0
0.33	0.17	0.17	0.33	0.125	0.125	0.5	0.25
0.33	0.5	0	0.17	0.1	0.1	0	0.8
0.67	0	0.17	0.17	0.6	0	0.2	0.2
0	0	1	0	0	0	1	0
0	0	0	1	0	0	0.2	0.8
0	0	0	1	0	0	0	1
0.5	0	0.5	0	0.5	0	0.5	0

Supplemental Files

Supplemental File S1. Multi alignment between protein sequences identified in *M. polymorpha*, *S. moellendorfii*, *E. hyemale*, *P. vittata*, *O. sativa* and *A. thaliana* belonging to the OG0000005 orthogroup.

Supplemental File S2. Multi alignment between protein sequences identified in *M. polymorpha*, *S. moellendorfii*, *E. hyemale*, *O. sativa* and *A. thaliana* belonging to the OG0000004 orthogroup.

Supplemental File S3. Multi alignment between protein sequences identified in *M. polymorpha*, *S. moellendorfii*, *E. hyemale*, *O. sativa* and *A. thaliana* belonging to the OG0007434 orthogroup.

Supplemental File S4. Multi alignment between protein sequences identified in *M. polymorpha*, *P. patens*, *S. moellendorfii*, *E. hyemale*, *O. sativa* and *A. thaliana* belonging to the OG0000715 orthogroup.

Supplemental File S5. Multi alignment between protein sequences identified in *M. polymorpha*, *P. patens*, *S. moellendorfii*, *E. hyemale*, *P. vittata*, *O. sativa* and *A. thaliana* belonging to the OG0000529 orthogroup.

Supplemental File S6. Multi alignment between protein sequences identified in *M. polymorpha*, *S. moellendorfii*, *E. hyemale*, *P. vittata*, *O. sativa* and *A. thaliana* belonging to the OG0000018 orthogroup.

Supplemental File S7. Multi alignment between protein sequences identified in *M. polymorpha*, *S. moellendorfii*, *E. hyemale*, *P. vittata* and *O. sativa* belonging to the OG0000016 orthogroup.

Supplemental File S8. Multi alignment between protein sequences identified in *P. patens*, *S. moellendorfii*, *E. hyemale*, *P. vittata*, *O. sativa* and *A. thaliana* belonging to the OG0000009 orthogroup.

Supplemental File S9. Multi alignment between *A. thaliana* five ERFVII protein sequences and the best ERF blast hits retrieved in bryophytes using AtRAP2.12 as query.

Supplemental File S10. Multi alignment between *A. thaliana* five ERFVII protein sequences and the best ERFVII orthologs identified in *Equisetum hyemale* and *Equisetum diffusum*, using AtRAP2.12 as query.

Supplemental Data S1. Unrooted phylogenetic tree of ERFVII orthologues in tracheophytes (lycophytes, eusporangiate ferns, leptosporangiate ferns, gymnosperms and angiosperms), with bootstrap values highlighted for each branch.

Supplemental Data S2. Unrooted phylogenetic tree of ERF proteins from bryophytes (*A. punctatus*, *M. polymorpha* and *P. patens*), lycophyte (*S. moellendorfii*) and angiosperm (*A. thaliana*), with bootstrap values highlighted for each branch.
