# Supplementary material for: ERFVIIs as transducers of oxygen-sensing in the evolution of land plant response to hypoxia
Source: Mol Plant. 2025 Jun 2;18(6):1072–87. doi: 10.1016/j.molp.2025.05.015 (PMC13448761; doi:10.1016/j.molp.2025.05.015)
Supplement: Supplementary file 2 [file mmc32.pdf]

CellPress
Partner Journal

Molecular Plant
Research article

ERFVIIs as transducers of oxygen-sensing in the evolution of land plant response to hypoxia

Laura Dalle Carbonare[1,2], Hans van Veen[3], Vinay Shukla[2], Monica Perri[2], Liem Bui[4], Michael J. Holdsworth[1,5,*] and Francesco Licausi[2,5,*]

[1]School of Biosciences, University of Nottingham, Loughborough LE12 5RD, UK

[2]Department of Biology, University of Oxford, Oxford OX1 3RB, UK

[3]Groningen Institute of Evolutionary Life Sciences, University of Groningen, Groningen, the Netherlands

[4]Biotechnology Research and Development Institute, Can Tho University, Can Tho, Vietnam

[5]These authors contributed equally to this article.

***Correspondence: Michael J. Holdsworth (michael.holdsworth@nottingham.ac.uk), Francesco Licausi (francesco.licausi@biology.ox.ac.uk)**

https://doi.org/10.1016/j.molp.2025.05.015

ABSTRACT

The transcriptional response to low oxygen (hypoxia) in flowering plants is mediated by group VII Ethylene Response Factor (ERFVII) transcription factors, whose proteolysis is oxygen-dependent via the PLANT CYSTEINE OXIDASE (PCO) N-degron pathway. However, when and how this hypoxia response evolved in land plants remains unknown. In this study, we investigated the conservation and divergence of transcriptional responses to hypoxia across major land plant clades. We found that the induction of gene functions associated with glycolysis and fermentation is part of a conserved response across all land plant divisions.Evolutinary analyses suggest that ERFVIIs emerged in the last common ancestor of vascular plants with true roots, coinciding with the evolution of oxygen-dependent destabilization mechanisms that regulate hypoxia-adaptive genes. Proteins from other ERF groups have been independently recruited multiple times in different clades as substrates of the PCO N-degron pathway. Taken together, our results demonstrate that the response of land plants to hypoxia has been refined in derived clades through the evolution of ERFVIIs as transcriptional transducers, which occurred concurrently with the emergence of vascular systems and roots as foraging structures in hypoxic soils.

Key words: ERFVII, oxygen-sensing, plant evolution, hypoxia-responsive orthogroups, transcription factor, N-degron pathway

Dalle Carbonare L., van Veen H., Shukla V., Perri M., Bui L., Holdsworth M.J., and Licausi F. (2025). ERFVIIs as transducers of oxygen-sensing in the evolution of land plant response to hypoxia. Mol. Plant. **18**, 1072–1087.

INTRODUCTION

The colonization of land by plants was one of the most important evolutionary events in Earth's history, as it shaped the atmosphere and terrestrial ecosystems (Morris et al., 2018). This event, which occurred around 480 million years ago, contributed to the stabilization and rise in molecular oxygen (O_2) concentration in the atmosphere by the end of the Ordovician period, primarily due to the photosynthetic activity of land plants (Donoghue et al., 2021). Fossil records indicate that primordial land plants were phenotypically similar to extant bryophytes and thrived in highly humid habitats such as riparian zones and marshes, which were frequentlysubjected to flooding events (Bowman et al., 2017).

One major perturbation experienced by plants during flooding is reduced diffusion of gases—particularly O_2—within tissues (Voesenek and Bailey-Serres, 2015). Responses to low oxygen availability (hypoxia) have been extensively studied in flowering plants—including the model species *Arabidopsis thaliana* and *Oryza sativa*—and involve the coordinated action of Group VII Ethylene Response Factor (ERFVII) transcription factors and Plant Cysteine Oxidase (PCO) oxygen-sensing enzymes via the PRT6 N-degron pathway (Gibbs et al., 2011; Licausi et al., 2011; Weits et al., 2014; White et al., 2017). Under normoxic conditions, constitutively expressed ERFVIIs are degraded via a conserved N-terminal cysteine (Cys), which is exposed following the removal of Met1 by constitutive Methionine Amino Peptidase. In the presence of O_2, PCO oxidizes the sulfur in the exposed N-terminal cysteine of the ERFVII degron to sulfinic acid. This modification facilitates arginylation by Arginyl Transferase, followed by polyubiquitylation through the combined actions of the E3 ubiquitin ligases PROTEOLYSIS 6 (PRT6) and BIG (Zhang et al., 2024), marking it for degradation by the

 © 2025 The Authors. Published by Elsevier Inc. on behalf of CAS Center for Excellence in Molecular Plant Sciences, Chinese Academy of Sciences, and Chinese Society for Plant Biology.
This is an open access article under the CC BY license (http://creativecommons.org/licenses/by/4.0/).

proteasome (Zubrycka et al., 2023). Nitric oxide (NO) also participates in this proteolytic pathway, although its precise mechanism of action remains unclear (Gibbs et al., 2014; Puerta et al., 2019). PCO activity is impaired under oxygen-limiting conditions and, consequently, stabilized ERFVIIs accumulate in the nucleus, where they regulate gene expression to promote plant adaptation to low O_2 conditions. Since O_2 serves as the terminal electron acceptor in oxidative phosphorylation, one of the primary effects of reduced O_2 availability in green plants is a metabolic shift from mitochondrial respiration to fermentation to sustain ATP synthesis (van Dongen and Licausi, 2015; Voesenek and Bailey-Serres, 2015; Bui et al., 2019).

Despite the conservation of the enzymatic components of the N-degron pathway across eukaryotes (Hammarlund et al., 2020; Holdsworth and Gibbs, 2020) and the critical role of low O_2 sensing in the adaptation of flowering plants to hypoxia (Xu et al., 2006; Weits et al., 2019; Abbas et al., 2022), our understanding of low O_2 sensing, signaling, and molecular responses outside angiosperms remains limited. This knowledge gap prevents comparative studies on the evolution of oxygen-sensing mechanisms in plants and animals. Notably, while recent studies have traced the origin of the metazoan Hypoxia Inducible Factor to the early Cryogenian (Mills et al., 2018; Song et al., 2022), a phylogenetic reconstruction of ERFVIIs as O_2-dependent N-degron substrates is still lacking. Here, we present a comprehensive analysis of the evolution of transcriptional regulation in response to hypoxia in land plants. We reconstruct the evolutionary history of ERFVIIs, analyze their regulation via the PRT6 N-degron pathway, and clarify their function as O_2-sensing transducers in land plants.

RESULTS

Transcriptional responses to hypoxia in land plants

We compared the transcriptional response to hypoxia across a range of species that recapitulate the major events in the evolution of land plants. We selected *Marchantia polymorpha* (liverwort) and *Physcomitrium patens* (moss) to represent bryophytes, *Selaginella moellendorffii* for lycophytes, and *Equisetum hyemale* and *Pteris vittata* for eusporangiate and leptosporangiate ferns, respectively (Figure 1A). Given the differences in life cycles among these divisions, we selected developmental stages that include the main photosynthetically active organs to ensure comparability of tissues with similar metabolic features. Therefore, we treated gametophores of *M. polymorpha* (n) and *P. patens* (n), as well as mature sporophytes of *S. moellendorffii* (2n), *E. hyemale* (2n), and *P. vittata* (2n) with 1% ambient O_2 under dark conditions for 8 h. Controls were maintained under aerobic conditions (21% ambient O_2) in darkness. Differentially expressed genes (DEGs) were identified through mRNA sequencing (Supplemental Tables 1–5). We compared the hypoxia responses of these five species with hypoxia transcriptome datasets available for photosynthetic sporophytes of angiosperms (*A. thaliana*; Licausi et al., 2011 and *Oryza sativa*; Narsai et al., 2009). The magnitude of the response varied across species. *P. patens* exhibited the fewest DEGs (68 upregulated [$\log_2 FC > 1$] and 30 downregulated [$\log_2 FC < -1$] genes, respectively, using a false discovery rate (FDR) $\leq$ 0.01) (Figure 1B). In contrast, *A. thaliana* and *O. sativa* showed the strongest transcriptional responses to the treatment (Figure 1B).

The diversification of genes across plant taxa strongly limited an exhaustive analysis of the conservation and divergence of hypoxia responses between lineages. To facilitate comparisons, we identified gene sets that originated from a single gene in the last common ancestor of all species studied, referred to as orthogroups (Wapinski et al., 2007). To define orthogroups, we used the OrthoFinder method (Emms and Kelly, 2015) on the transcriptomes of 33 species representing 9 clades: dicot and monocot angiosperms, gymnosperms, leptosporangiate ferns, horsetail ferns, lycophytes, mosses, liverworts, and hornworts. This included the 7 species for which we generated or retrieved hypoxia transcriptomes (Supplemental Tables 6–12). We identified 4969 orthogroups conserved across all clades, as well as 1543 orthogroups gained and 808 orthogroups lost in angiosperms. Additionally, a total of 10 281 orthogroups were found to be specific to 1 of the 9 clades (Figure 1C and Supplemental Table 13, Supplemental Figures 1A, 1B, and 2A). Among conserved orthogroups, our analysis revealed a high number of orthologs shared among more closely related species (Supplemental Figure 2B; Supplemental Table 14).

Given the considerable phylogenetic breadth of this study, most orthogroups are represented by multiple genes within a single species. For 96% of the orthogroups, the average gene count per species ranged from 1 to 8 (Supplemental Figure 3; Supplemental Table 15), potentially leading to divergent hypoxic responses within the same orthogroup. However, only 3.066% of DEG-containing orthogroups ($|\log_2 FC > 1|$ and *p*adj. < 0.05) had both up- and downregulated genes within a single species (Figure 1D; Supplemental Table 16). This suggests that coordinated regulation of gene expression among highly similar genes within a species is more prevalent than compensatory mechanisms or isoform switching. Moreover, this approach allows orthogroups to serve as a framework for directly comparing transcriptomic responses among distantly related species and identifying both differential and shared hypoxia responses in the seven species analyzed. We defined an orthogroup as up- or downregulated in a given species if it contained at least one DEG ($|\log_2 FC > 1|$ and *padj*. < 0.05). Overall, the strongest transcriptional response to hypoxia, in terms of number of regulated orthogroups, was observed in *O. sativa* and *A. thaliana* (Figure 1D). Notably, numerousorthogroups—classified as conserved, gained, lost, or clade-specific—were downregulated by hypoxia in these two angiosperms (Figure 1D and 1E). Substantial downregulation was also observed in *M. polymorpha*, whereas this response was markedly attenuated in other species and nearly absent in *P. patens* and *S. moellendorffii*. Furthermore, the strong regulation observed in angiosperms and weaker regulation in other lineages was not restricted to newly evolved, lost, or clade-specific orthogroups, but was equally evident among conserved orthogroups.

The molecular function of conserved hypoxia-regulated orthogroups

We analyzed the extent of functional overlap among regulated orthogroups across species. To this end, we compared the similarity of up- and downregulated orthogroups between all species pairs, normalized by the total number of shared orthogroups (Supplemental Figure 2B). As expected, the greatest overlap in

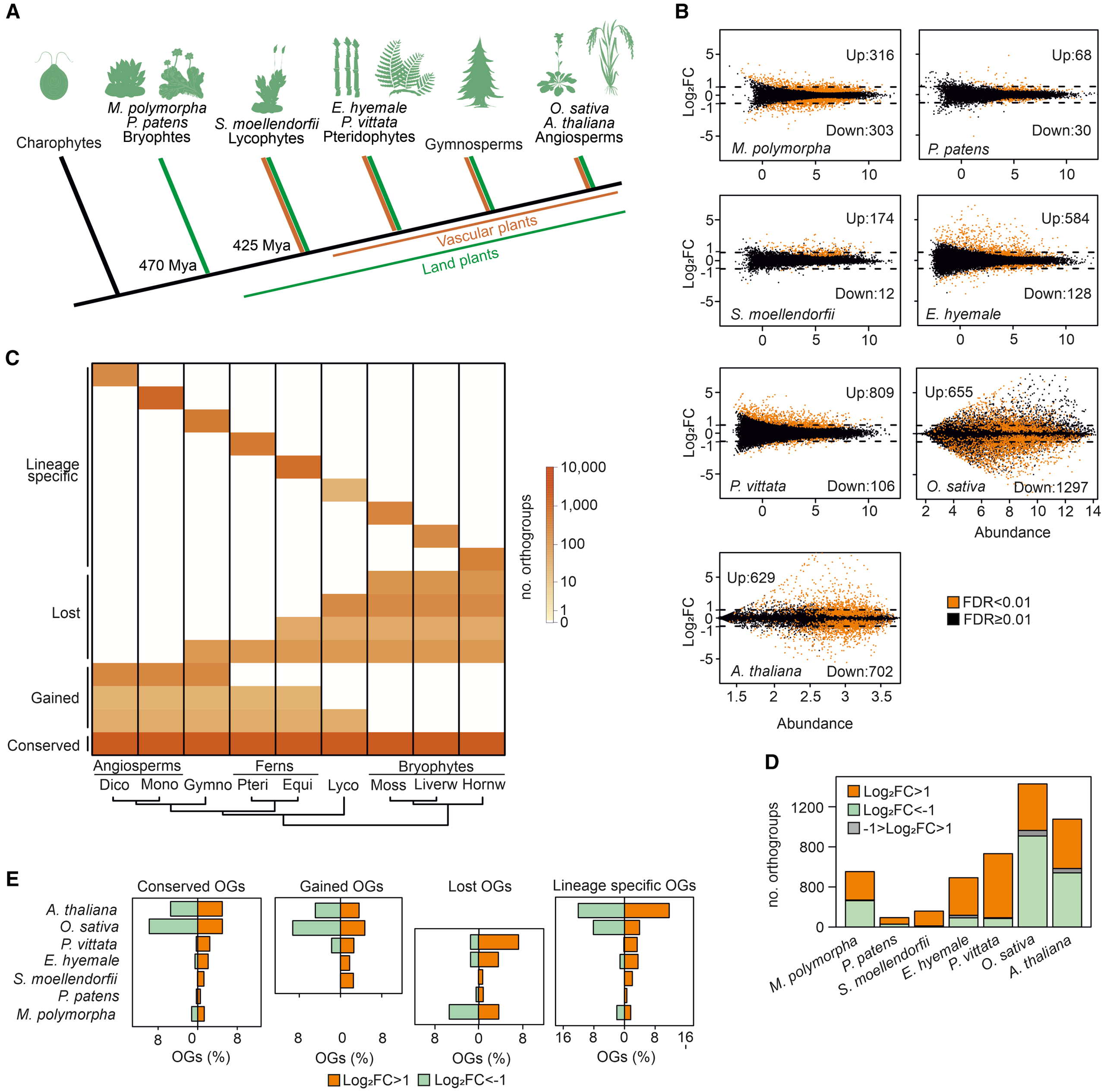

Figure 1. The transcriptional responses to hypoxia across land plant divisions.

(A) Timeline depicting the divergence of major phylogenetic groups. The first land plants colonized terrestrial environments around 470 million years ago (Mya) and possessed features similar to extant bryophytes, whereas the first vascular plants appeared on land approximately 425 Mya (Donoghue et al., 2021).

(B) Funnel plots showing changes in gene expression, expressed as $\log_2$FC, in response to 8 h of hypoxia treatment (1% v/v O_2) compared with normoxia in *M. polymorpha*, *P. patens*, *S. moellendorffii*, *E. hyemale*, *P. vittata*, *O. sativa*, and *A. thaliana*.

(C) Heatmap displaying the number of orthogroups categorized as "conserved," "gained," "lost," or "lineage-specific", for each major group of land plants.

(D) Bar plot showing the number of orthogroups containing upregulated ($\log_2$FC > 1), downregulated ($\log_2$FC < −1), or simultaneously up- and down-regulated (−1> $\log_2$FC > 1) genes for the dataset described in **(B)**.

(E) Bar plots showing the percentage (%) of orthogroups upregulated ($\log_2$FC > 1) or downregulated ($\log_2$FC < −1) by hypoxia (1% v/v O_2, 8 h) for the seven species described in **(B)**. Orthogroups are classified as "conserved," "gained," "lost," or "lineage-specific" (Supplemental Figures 1 and 2A).

regulation was observed among more closely related species; for example, tracheophytes exhibited more similar regulatory patterns than bryophytes (Figure 2A). However, this trend did not extend to repressed orthogroups: in *S. moellendorffii*, only a few genes were downregulated in response to hypoxia, while in *P. vittata*, a unique set of orthogroups—mainly in the gained and lost categories—was repressed (Figure 1E). Both pteridophyte species, *P. vittata* and *E. hyemale*, shared a

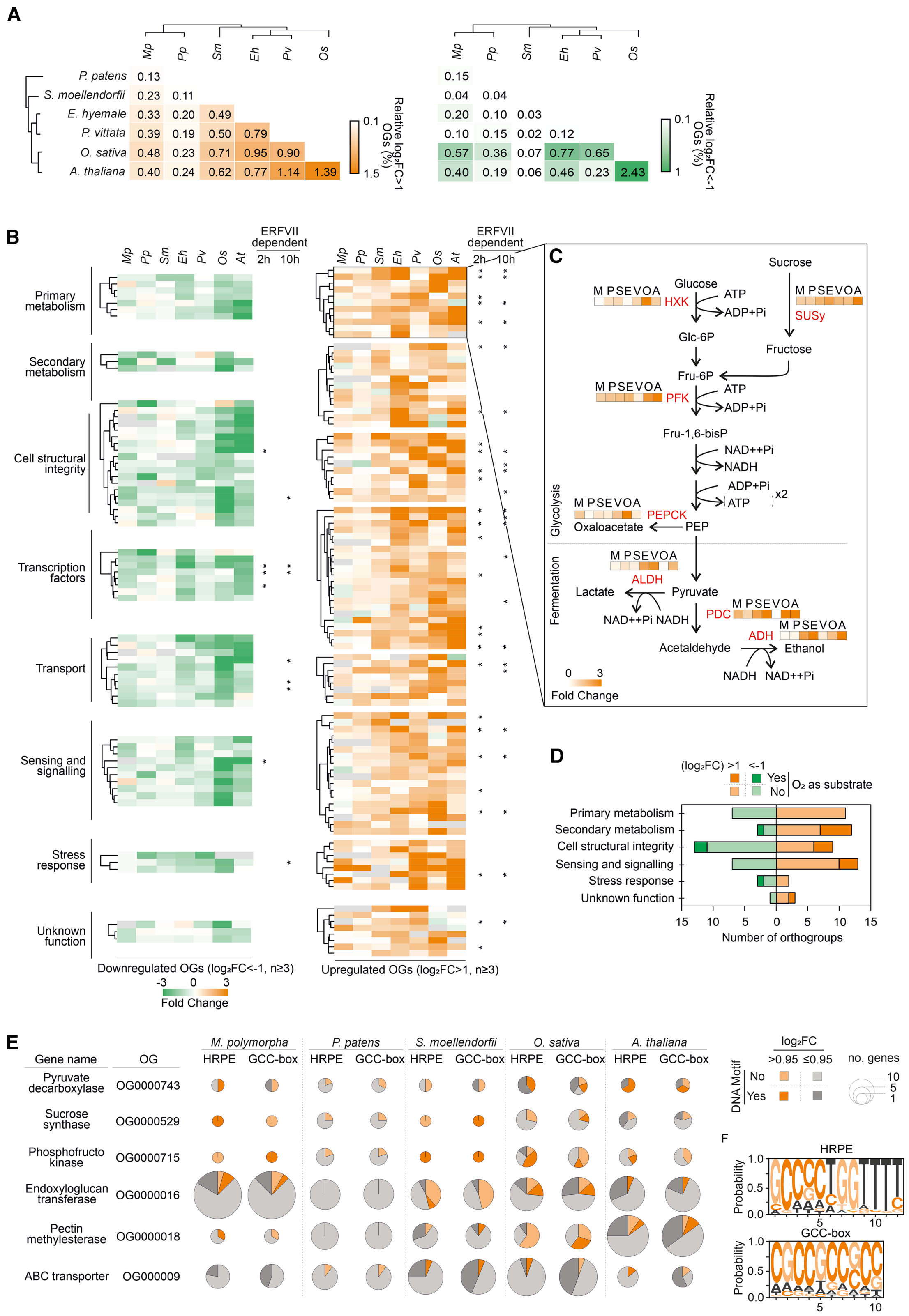

Figure 2. Evolution of the transcriptional responses to hypoxia in land plants.

(A) Pairwise comparisons of differentially regulated (|log_2FC|>1) orthogroups among the six species analyzed in this study, normalized by the total number of shared orthogroups for each pair.

(legend continued on next page)

similar number of repressed orthogroups with angiosperms. Angiosperms themselves shared the highest proportion of up- and downregulated orthogroups.

To further explore the conservation of the transcriptional response to hypoxia in land plants, we focused on orthogroups exhibiting similar regulation across species. Given the relatively low conservation across all species, we identified orthogroups that were regulatedin at least three clades to capture shared regulatory patterns across major plant divisions. Using this criterion, we identified 78 conserved upregulated orthogroups and 54 downregulated orthogroups. These were grouped according to the molecular function of their *A. thaliana* orthologs, where available (Figure 2B; Supplemental Tables 17 and 18). These orthogroups contained genes involved in primary and secondary metabolism, cell structure integrity, small molecule and ion transport, regulation of mRNA transcription, signal transduction, and stress responses (Figure 2B). Several orthogroups could not be assigned a function and were classified as "unknown." Many of the conserved upregulated orthogroups include proteins known to be part of the "core anaerobic response" in *A. thaliana* (Mustroph et al., 2009). Most of the highly conserved induced orthogroups—those upregulated in six out of seven species—belong to the "metabolism" category, including genes involved in glycolytic and fermentative pathways, such as sucrose synthase, ATP-dependent phosphofructokinase, and pyruvate decarboxylase (PDC) (Figure 2C). Remarkably, several genes included in these conserved induced, but not repressed, orthogroups encode signaling and metabolic enzymes that use molecular oxygen as a co-substrate (Figure 2D; Supplemental Tables 17, 18, and 19). Among transcriptional regulators, the ERF and MYB families were universally represented, although sequence similarity of upregulated genes within these orthogroups was limited to the DNA-binding domain—in contrast to the high similarity observed throughout the entire sequence in metabolic enzymes (Supplemental Files 1, 2, 3, 4, 5, 6, 7, and 8).

A transcriptome analysis comparing wild-type *A. thaliana* and the pentuple *erfVII* mutant (which lacks all ERFVII activity; *rap2.12 rap2.2 rap2.3 hre1 hre2*; Abbas et al., 2015), subjected to 2 and 10 h of hypoxia (Supplemental Table 20), revealed that approximately half of the genes induced after 2 h of hypoxia in the wild type were expressed at lower levels in the *erfVII* mutant. In contrast, the absence of ERFVII activity affected only about one-fifth of the hypoxia-repressed genes. After 10 h of hypoxia, only one-fifth of the induced genes exhibited ERFVII dependency, suggesting that additional regulatory mechanisms act at later time points to control transcript abundance (Supplemental Figure 4). Notably, several of the conserved upregulated orthogroups contained *A. thaliana* genes that require ERFVIIs for induction, whereas only a few of the downregulated orthogroups showed this feature (Figure 2B; Supplemental Tables 17 and 18) (Giuntoli et al., 2017; Zubrycka et al., 2023).

Among hypoxia-induced transcription factors, ERF and MYB orthogroups were the most conserved, being upregulated in at least six of the seven species examined. To assess their potential regulatory roles, we tested for enrichment of their respective promoter binding elements among differentially upregulated genes. At the genome-wide level, we observed significant enrichment only for the Hypoxia Responsive Promoter Element (HRPE) motif in angiosperms and for the GCC box in *P. patens* and *S. moellendorffii* (Supplemental Figure 5; Supplemental Tables 21–25). However, analysis of consensus DNA sequences in the promoters of genes belonging to orthogroups with similarly conserved regulation revealed a more nuanced picture (Figure 2E and 2F; Supplemental Tables 26–30). The DNA sequences generally recognized by ERF proteins (the GCC-box) (Hao et al., 1998) and the ERFVII-specific HRPE motif (Gasch et al., 2016) were individually present in at least one promoter of most, but not all, induced genes in the orthogroups. These motifs were sometimes also found in promoters of genes not responsive to hypoxia, indicating that their presence alone is not sufficient for regulation by ERFVIIs. The HRPE and GCC elements were rarely detected in *P. patens* promoters but were frequently found in those of *M. polymorpha* and the tracheophytes analyzed. The two DNA elements rococognized by R2R3-MYB TFs (MYB-I and MYB-II) (Kelemen et al., 2015) were enriched in the promoters of upregulated orthogroups in *O. sativa* and *A. thaliana*, and more widely distributed in the promoters of downregulated orthogroups, although they were absent in *P. patens* (Supplemental Figure 6A). The motif recognized by membrane-tethered NAC transcription factors (MDM) (De Clercq et al., 2013)—which have been implicated in alleviating oxidative stress associated with submergence and reoxygenation in *A. thaliana* (Giuntoli et al., 2017)—was not enriched in our cross-species dataset (Supplemental Figure 6B).

Evolution of ERFVIIs in land plants

The enrichment of ERF-recognized DNA elements and the conservation of ERF-dependent genes among hypoxia-inducible orthogroups across land plants prompted us to investigate the

(B) Heatmaps showing shared upregulated ($log_2FC > 1$) and downregulated ($log_2FC < -1$) orthogroups between at least three species, grouped by molecular function. Orthogroups whose hypoxia regulation (2 and 10 h treatments) is ERFVII-dependent in *A. thaliana* (Supplemental Figure 4) are marked with an asterisk.

(C) Simplified schematic of the glycolytic and fermentation pathways in plants. Enzymes corresponding to conserved upregulated orthogroups within the "metabolism" category are highlighted in red. The degree of upregulation for each orthogroup is shown in the adjacent heatmap. Species abbreviations: M (*M. polymorpha*), P (*P. patens*), S (*S. moellendorffii*), E (*E. hyemale*), V (*P. vittata*), O (*O. sativa*), and A (*A. thaliana*).

(D) Number of enzymes among the conserved hypoxia-inducible ($log_2FC > 1$) or -repressed ($log_2FC < -1$) orthogroups that use O_2 as a substrate or do not require O_2.

(E) Pie charts showing the frequency of HRPE or GCC-box *cis*-elements found in promoters of upregulated ($log_2FC > 0.95$) or unchanged ($log_2FC \leq 0.95$) genes belonging to conserved orthogroups under hypoxia. The size of each pie chart is proportional to the number of genes included in each orthogroup per species.

(F) HRPE and GCC-box motif logos, adapted from Gasch et al. (2016).

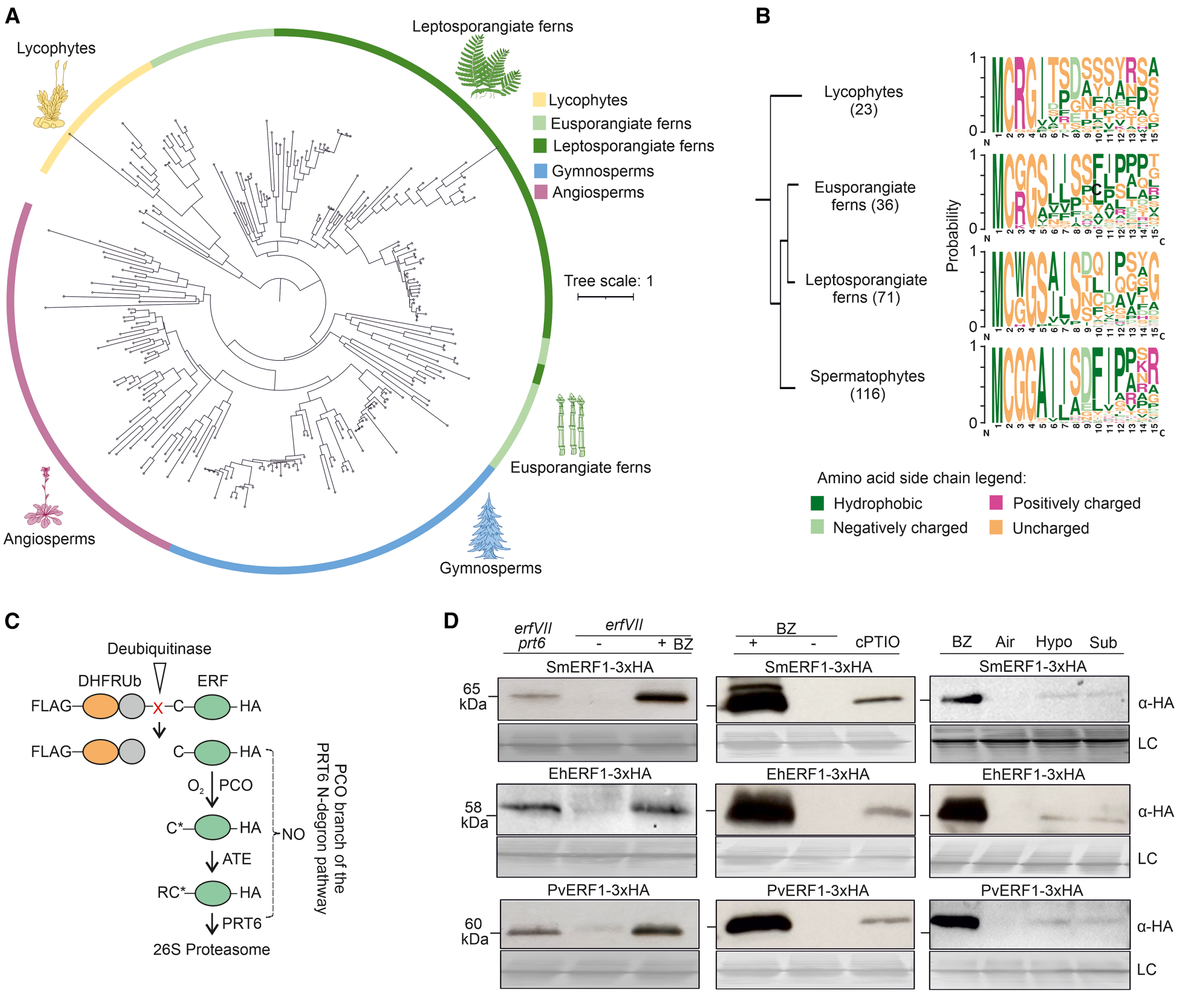

Figure 3. Evolution of group VII ERFs in tracheophytes.

(A) Rooted phylogenetic tree of ERFVII orthologs in tracheophytes (lycophytes, eusporangiate ferns, leptosporangiate ferns, gymnosperms, and angiosperms). Bootstrap values are shown in Supplemental Dataset 6.

(B) Motif logo of the first 15 N-terminal amino acids of the ERFVII sequences used to construct the phylogenetic tree shown in **(A)**. The number of species included in each group is indicated in brackets.

(C) Schematic representation of the ubiquitin fusion construct (Zubrycka et al., 2023) used to study protein stability of different ERFVIIs, each tagged with 3×HA for immunoblot detection through the PCO branch of the PRT6 N-degron pathway.

(D) Western blot analysis of SmERF1^{3xHA}, EhERF1^{3xHA}, and PvERF1^{3xHA} abundance in control (DMSO), bortezomib (BZ), NO-scavenger (cPTIO), dark air (2 h), dark hypoxia (2 h, 0.1% v/v O_2), or dark submergence (1 h) treated *erfVII* or *erfVII prt6* mutant 7-day-old *A. thaliana* seedlings. Loading control (LC) corresponds to a re-fitted and compacted image of the membrane after Ponceau staining. Unedited images are shown in Supplemental Figures 15 and 16.

evolutionary breadth of ERFVII involvement in regulating transcriptional responses to acute hypoxia within the green lineage. Because our orthogroup analysis could not fully resolve groups within the ERF subfamily (Nakano et al., 2006), we performed a reciprocal best hits analysis using the *A. thaliana* RAP2.12 protein as a query against genome-predicted proteomes from 104 land plant species (12 bryophytes and 92 tracheophytes) and 2 algae species (1 chlorophyte and 1 charophyte). For each tracheophyte species, we retrieved at least 1 protein that shared similarity with *A. thaliana* ERFVIIs beyond the AP2 domain (Supplemental Table 31). In contrast, only a few bryophyte species contained sequences similar to *A. thaliana* ERFVIIs, while in most bryophytes and algae, sequence similarity was restricted to the AP2 DNA-binding domain (Supplemental File 9; Supplemental Tables 32 and 33). We used all retrieved tracheophyte ERFVII-like proteins to construct a phylogenetic tree, which recapitulated species division into four major clades: lycophytes, ferns, gymnosperms, and angiosperms (Figure 3A). The fern branch in our tree did not separate eusporangiate and leptosporangiate species, suggesting that ERFVII divergence predated the split between these two lineages (Figure 3A). Additionally, a group of ERFVIIs from the genus *Equisetum*

shared such high sequence similarity to gymnosperm ERFVIIs that they clustered within the same branch of the tree (Figure 3A; Supplemental File 10). A detailed inspection of the N-terminus of ERFVII-like proteins (first 15 amino acids [aa]) confirmed the extreme sequence conservation previously reported in angiosperms (Nakano et al., 2006; Holdsworth and Gibbs, 2020). Specifically, the Cys2 residue required for oxygen-regulated degradation (Gibbs et al., 2011; Licausi et al., 2011) is present in lycophyte and fern ERFVII-like proteins. However, the remainder of the N-terminal sequence diverges, displaying high variability from the 5th to 6th residues onward (Figure 3B). Gly is the preferred residue at the 4th position across all tracheophytes, while Arg predominates at the 3rd position in lycophytes and is often found in eusporangiate ferns (Figure 3B). In approximately half of the fern sequences and in spermatophytes, Gly replaces Arg at the 3rd position generating the MCGGAI consensus previously reported for angiosperms. Notably, leptosporangiate ERFVII-like proteins often contain a Trp at the 3rd position (Figure 3B). To test whether ERFVII-like proteins from lycophytes and ferns are substrates of the PRT6 N-degron and ubiquitin proteasome (UPS) pathways (Figure 3C), we cloned the coding sequences of ERFVII-like protein genes from *S. moellendorffii*, *E. hyemale*, and *P. vittata* downstream of a dihydrofolate reductase and Ubiquitin (Ub) fusion, and expressed the fusion protein under the control of the CaMV 35S promoter (Varshavsky, 2011; Zubrycka et al., 2023). This construct allows assessment of ERFVII protein stability after co-translational release from the precursor polypeptide by constitutive deubiquitinase activity (Figure 3C). Pulse-chase experiments using coupled transcription/translation in rabbit reticulocyte extracts revealed that these proteins are unstable following translational shutdown with cycloheximide; however, their half-life was increased in the presence of the proteasome inhibitor bortezomib (BZ) or in constructs with a Cys2Ala mutation (Supplemental Figure 7A and 7B), indicating that their stability depends on the presence of Cys2. We further assessed PRT6- and UPS-mediated degradation *in vivo* in *A. thaliana*. When expressed in the *A. thaliana erfVII* mutant, these ERFVII-like proteins were highly unstable and failed to accumulate in seedling tissues. In contrast, their accumulation increased either by genetic inactivation of the N-recognin PRT6 (in the *erfVII prt6* sextuple mutant) or by application of the proteasome inhibitor BZ (Figure 3D). Similarly, low O_2 conditions—and, even more strongly, scavenging of NO with cPTIO—led to stabilization of these ERFVII proteins (Figure 3D and Supplemental Figure 7C and 7D). Notably, the increase in ERFVII levels under low oxygen was smaller than that achieved by proteasome inhibition (Figure 3D), possibly reflecting the involvement of multiple proteolytic pathways. Together, these results confirm that lycophyte and fern ERFVII-like proteins are substrates of the NO- and O_2-dependent branch of the N-degron pathway in *A. thaliana*. Given the conserved nature of this pathway in eukaryotes (Holdsworth and Gibbs, 2020; Weits et al., 2023), it is likely that ERFVIIs also act as bona fide PCO N-degron pathway substrates in their species of origin.

Non-ERFVII ERF proteins have been recruited as substrates of the PCO N-degron pathway in bryophytes

Analysis of algal genomes revealed no ERFVII-like proteins or ERF proteins containing Cys2 (Supplemental Table 33). Within the Marchantiophyta, *M. polymorpha* (MpERF18, Mapoly0092s0058), along with other Marchantiopsida and Jungermannopsida species, includes proteins with similarity to angiosperm ERFVIIs, although none contained Cys2 (Supplemental Figure 8; Supplemental Table 34). While these sequences clustered with *A. thaliana* and *S. moellendorffii* ERFVIIs, similar proteins were absent in moss (*P. patens*) and hornwort (*Anthoceros punctatus*) (Supplemental Figure 9). However, we identified several ERF proteins with a conserved Cys2 residue (Cys2-ERFs) (Supplemental Table 35). A genome-wide analysis of the entire ERF family in *M. polymorpha*, *P. patens*, *S. moellendorffii*, and *A. punctatus* revealed a conserved set of Cys2-ERFs with strong sequence similarity to ERF group X, group IV (in *M. polymorpha*), and group VIII (in *P. patens*) (Figure 4A). The conserved group X Cys2-ERFs exhibited an extended N-terminal consensus sequence, with positively charged residues frequently occupying the third position—a feature also observed in ERFVIIs from lycophytes and eusporangiate ferns (Figures 3B and 4B). To test whether Cys2-ERFs from *M. polymorpha* and *P. patens* are substrates of the PCO N-degron pathway, we conducted both *in vitro* assays in rabbit reticulocyte extracts and *in vivo* experiments in *A. thaliana* seedlings, following the same procedures used for tracheophyte ERFVII-like proteins (Figure 3C). These assays indicated that the Cys2-ERF proteins are unstable unless proteasome activity is inhibited or N-degron processing is blocked—either genetically (*erfVII prt6* background) or chemically via NO scavenging with cPTIO (Figure 4C and Supplemental Figure 10). However, unlike tracheophyte sequences, we did not observe stabilization of bryophyte Cys2-ERFs in response to hypoxia or submergence, whereas an *A. thaliana* ERFVII RAP2.3-based reporter (Zubrycka et al., 2023) exhibited the expected stabilization under both conditions (Supplemental Figure 7C and 7D). This suggests that the apparent insensitivity of bryophyte Cys2-ERFs to hypoxia could be due to sustained Cys oxidation by *A. thaliana* PCOs under low-oxygen conditions. Based on these findings, we speculate that while bryophyte Cys2-ERF proteolysis is insensitive to hypoxia, the affinity of tracheophyte ERFVIIs for PCOs is optimally reduced by Gly and positively charged residues, thereby preventing oxidation and subsequent degradation under hypoxic conditions. Indeed, a fusion of a 50-aa N-terminal fragment of *A. thaliana* RAP2.12 to the fluorescent protein citrine exhibited increased accumulation under hypoxia when expressed in *M. polymorpha* (Figure 4D and Supplemental Figure 11), confirming the intrinsic property of tracheophyte ERFVIIs to be regulated by O_2 availability. Additionally, we found that the only *M. polymorpha* Cys2-ERF (MpERF23, Mapoly0293s0001) is not required for the induction of genes involved in primary metabolism under hypoxia: CRISPR-mediated inactivation of *MpERF23* did not affect the expression of *PDC2*, the mitochondrial sulfur dioxygenase *ETHYLMALONIC ENCEPHALOPATHY PROTEIN1* (*ETHE1*) or *L2-HYDROXYGLUTARATE DEHYDROGENASE* (*L2HGDH*) in *M. polymorpha* (Figure 4E).

Evolution of Cys2-ERFs as oxygen-dependent transcriptional regulators

We set out to assess whether the bryophyte and tracheophyte Cys2-ERF—substrates of the PCO N-degron pathway—could substitute for the endogenous *A. thaliana* ERFVIIs in activating

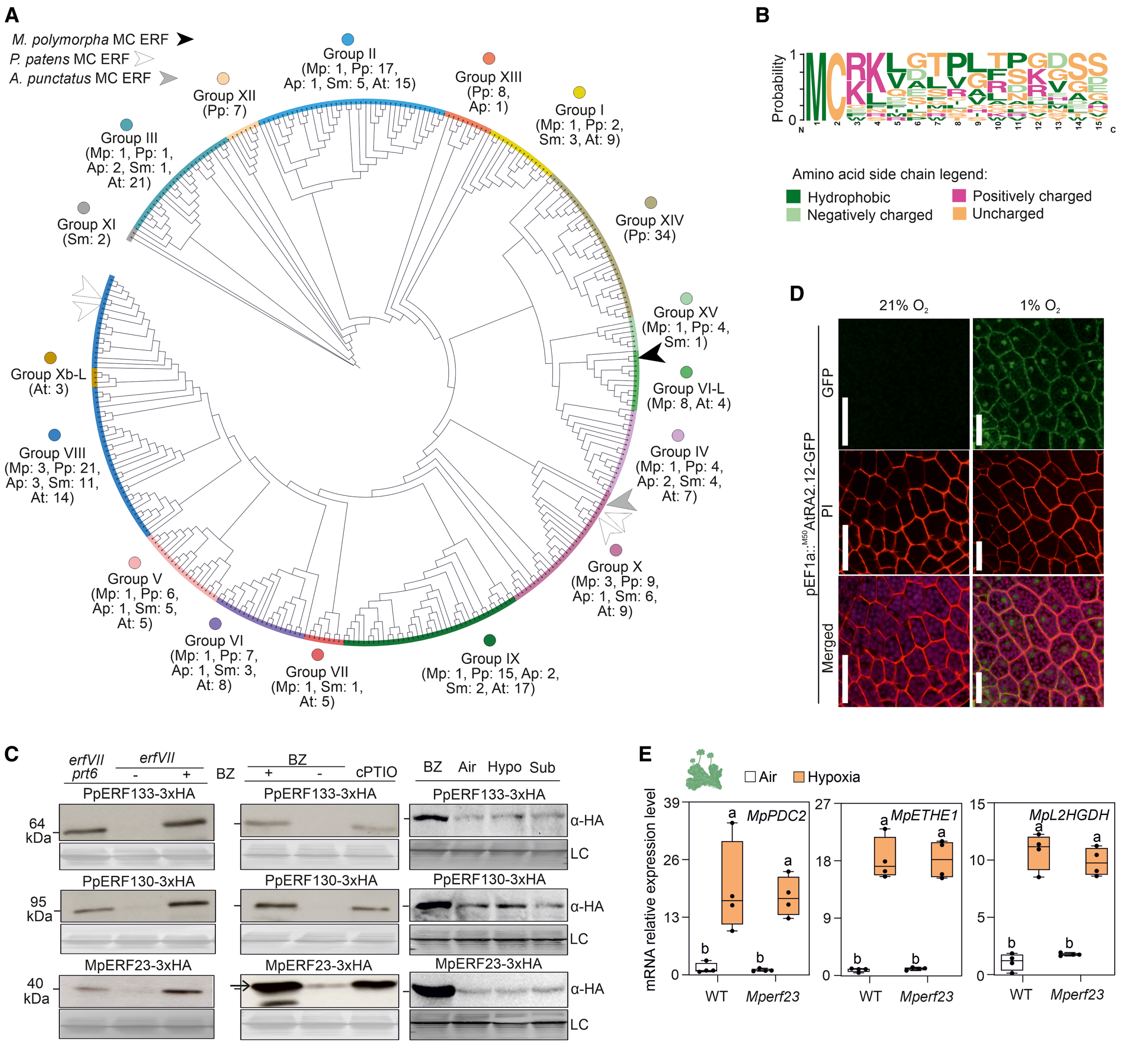

Figure 4. Characterization of Cys2-ERF proteins in bryophytes.
(A) Unrooted phylogenetic tree of ERF proteins from bryophytes (*A. punctatus*, *M. polymorpha*, and *P. patens*), lycophyte (*S. moellendorffii*), and angiosperm (*A. thaliana*). ERF classification is based on Nakano et al. (2006). White, gray, and black arrowheads indicate Cys2 ERFs in *P. patens*, *A. punctatus*, and *M. polymorpha*, respectively. Bootstrap values are provided in Supplemental Dataset 7.
(B) Motif logo of the first 15 N-terminal amino acids of Cys2-ERF group X sequences retrieved in bryophytes (liverworts, mosses, and hornworts).
(C) Western blot analysis of PpERF133^{3xHA}, PpERF130^{3xHA}, and MpERF23^{3xHA} abundance in control (DMSO), bortezomib (BZ), NO-scavenger (cPTIO), dark air (2 h), dark hypoxia (2 h, 0.1% v/v O_2), or dark submergence (1 h) treated *erfVII* or *erfVII prt6* mutant 7-day-old *A. thaliana* seedlings. The black arrow indicates the protein band with the expected size. Loading control (LC) corresponds to a re-fitted and compacted image of the membrane after Ponceau staining. Unedited images are shown in Supplemental Figures 15 and 16.
(D) Confocal images showing the subcellular localization of $^{1-50}$AtRAP2.12-Citrine, overexpressed in 4-day-old *M. polymorpha* gemmae under air (21% ambient O_2) and dark hypoxia (1% v/v O_2) (Supplemental Figure 11).
(E) Effect of MpERF23 on *MpPDC2*, *MpETHE1*, and *MpL2HGDH* expression levels in dark air and dark hypoxia (1% v/v O_2) in wild-type compared with *Mperf23* knockout 13-day-old *M. polymorpha* thalli. Data are presented as means ± SD ($n = 4$). Different letters indicate statistically significant differences determined by two-way ANOVA followed by Holm-Sidak post-hoc test ($p < 0.05$).

hypoxia-related gene expression in seedlings. Under submergence, no significant changes in the expression of conserved hypoxia-activated genes were observed in plants containing Cys2-ERF transgenes compared with the *erfVII* mutant (Figure 5A). However, non-vascular Cys2-ERFs increased the expression of *PDC1*, and MpERF23 could also induce *ADH1* in the *erfVII prt6* background, where the Arg-branch of the N-degron pathway is inhibited (Figure 5B). It is possible that

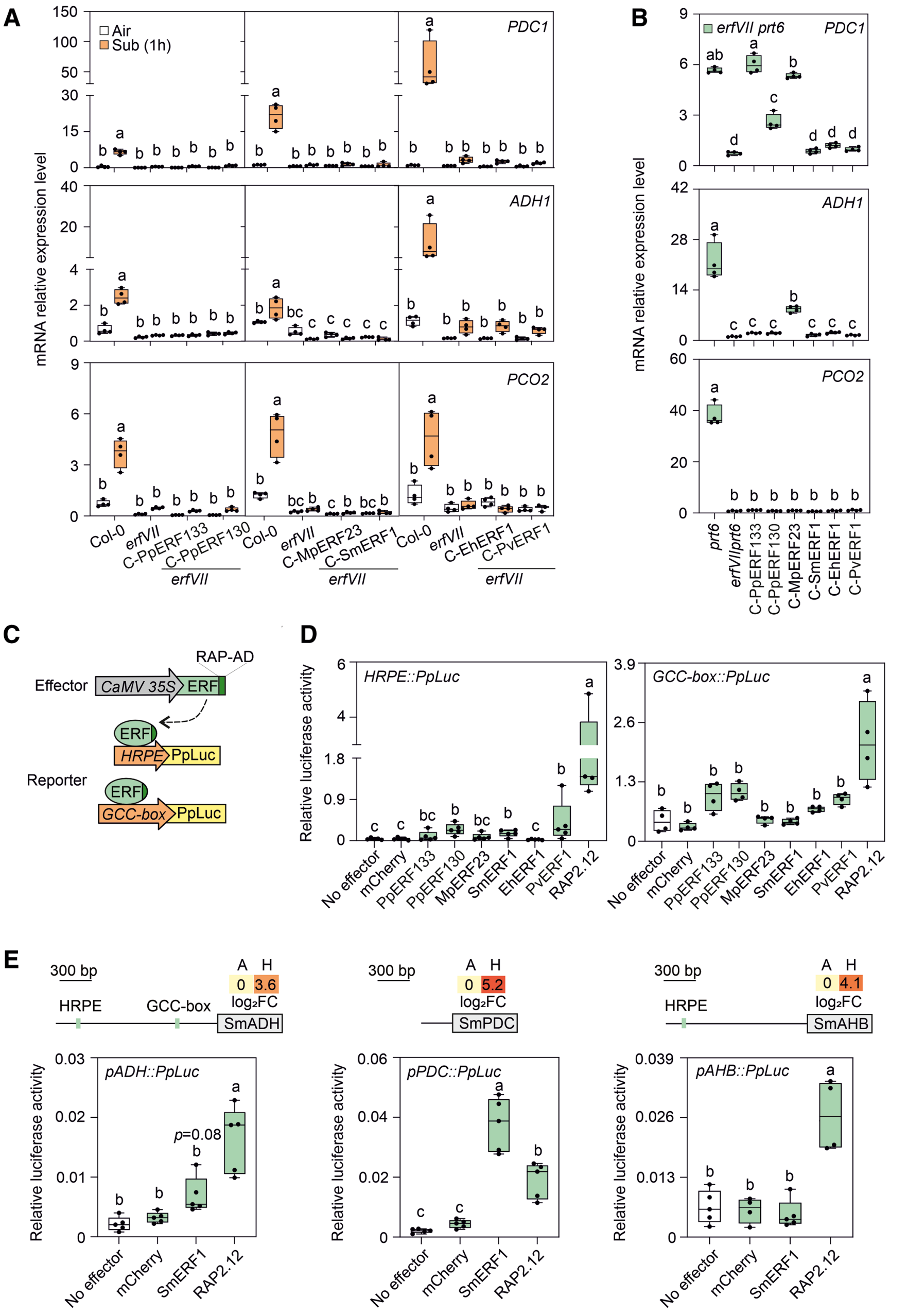

Figure 5. Potential roles of Cys2-ERFs and non-angiosperm ERFVIIs in regulating the expression of hypoxia-responsive genes in *A. thaliana*.

(A) Effect of Cys2-ERFs or ERFVIIs on the expression of *PDC1*, *ADH1*, and *PCO2* in 7-day-old *A. thaliana erfVII* seedlings following 1 h of dark submergence compared with aerobic conditions.

(B) Effect of Cys2-ERFs or ERFVIIs on the expression of *PDC1*, *ADH1*, and *PCO2* in 7-day-old *A. thaliana erfVII prt6* seedlings.

(C) Schematic of the effector (ERF coding sequence fused to the AtRAP2.12 activation domain under the constitutive CaMV *35S* promoter) and reporter (firefly luciferase coding sequence under the synthetic, hypoxia-inducible HRPE or ERF-specific GCC-box promoter) constructs used in the protoplast transactivation assay in **(D)**.

(D) Effect of Cys2-ERFs or ERFVIIs on synthetic HRPE and GCC-box promoters, measured as relative firefly luciferase activity in *A. thaliana erfVII prt6* mesophyll protoplasts.

(E) Effect of SmERF1 and RAP2.12 on *S. moellendorffii* hypoxia-inducible promoters (*SmADH*, *SmPDC*, and *SmAHB*; log2FC in hypoxia vs. air reported in Supplemental Table 3) measured as relative firefly luciferase activity in *A. thaliana erfVII prt6* mesophyll protoplasts. Positions of predicted HRPE and GCC-box elements in the promoter regions are indicated. In **(D)** and **(E)**, data are presented as means ± SD (n = 5). Different letters indicate statistically significant differences as assessed by one-way or two-way ANOVA followed by Tukey's or Holm-Sidak post-hoc tests, respectively ($p < 0.05$).

transiently accumulated Cys2-ERFs are unable to interact with the *Arabidopsis* transcriptional machinery required for gene activation. Persistence of these transcription factors throughout the life of the plant, as occurs in N-degron mutants, might overcome this limitation.

To test this hypothesis, we fused the activation domain of *A. thaliana* RAP2.12 (aa 340–358) (Bui et al., 2015) to each Cys2-ERF and assessed their ability to transactivate an HRPE- or GCC-box-containing synthetic promoter in *A. thaliana* mesophyll protoplasts (Figure 5C). The Cys2-ERFs from *P. patens*, *S. moellendorffii*, and *P. vittata* could significantly induce the luciferase reporter gene driven by the HRPE promoter, albeit at a lower level than RAP2.12, but were unable to activate the GCC-box-containing promoter (Figure 5D). This suggests that HRPE-binding is not restricted to ERFVIIs, and that other phylogenetically distant Cys2-ERFs also possess specificity for this DNA motif (Figure 2D). To explore the ancestral role of HRPEs in mediating hypoxia responsiveness in *cis*, we cloned three *S. moellendorffii* genomic regions upstream of hypoxia-induced genes (ADH_{prom}, PDC_{prom}, and HB_{prom}) and tested whether they could be activated by RAP2.12 or SmERF1 in *A. thaliana* protoplasts. RAP2.12 activated all three promoters, whereas SmERF1 activated only two, suggesting that the regulation of hypoxia-responsive genes in lycophytes is likely mediated by ERFVIIs, as demonstrated in angiosperms (Licausi et al., 2011), though this apparently does not require the presence of an HRPE- or GCC-box motif (Figure 5E). While our assay did not directly test for DNA binding, these results suggest that the function of HRPEs depends on the DNA context in which they occur. A protoplast assay also showed that the *M. polymorpha* ERFVII-like protein—similar to angiosperm ERFVIIs but lacking Cys2—can regulate expression through the synthetic HRPE promoter, but not the GCC-box (Supplemental Figure 12), supporting the hypothesis of an early partnership between ERFVIIs and HRPE.

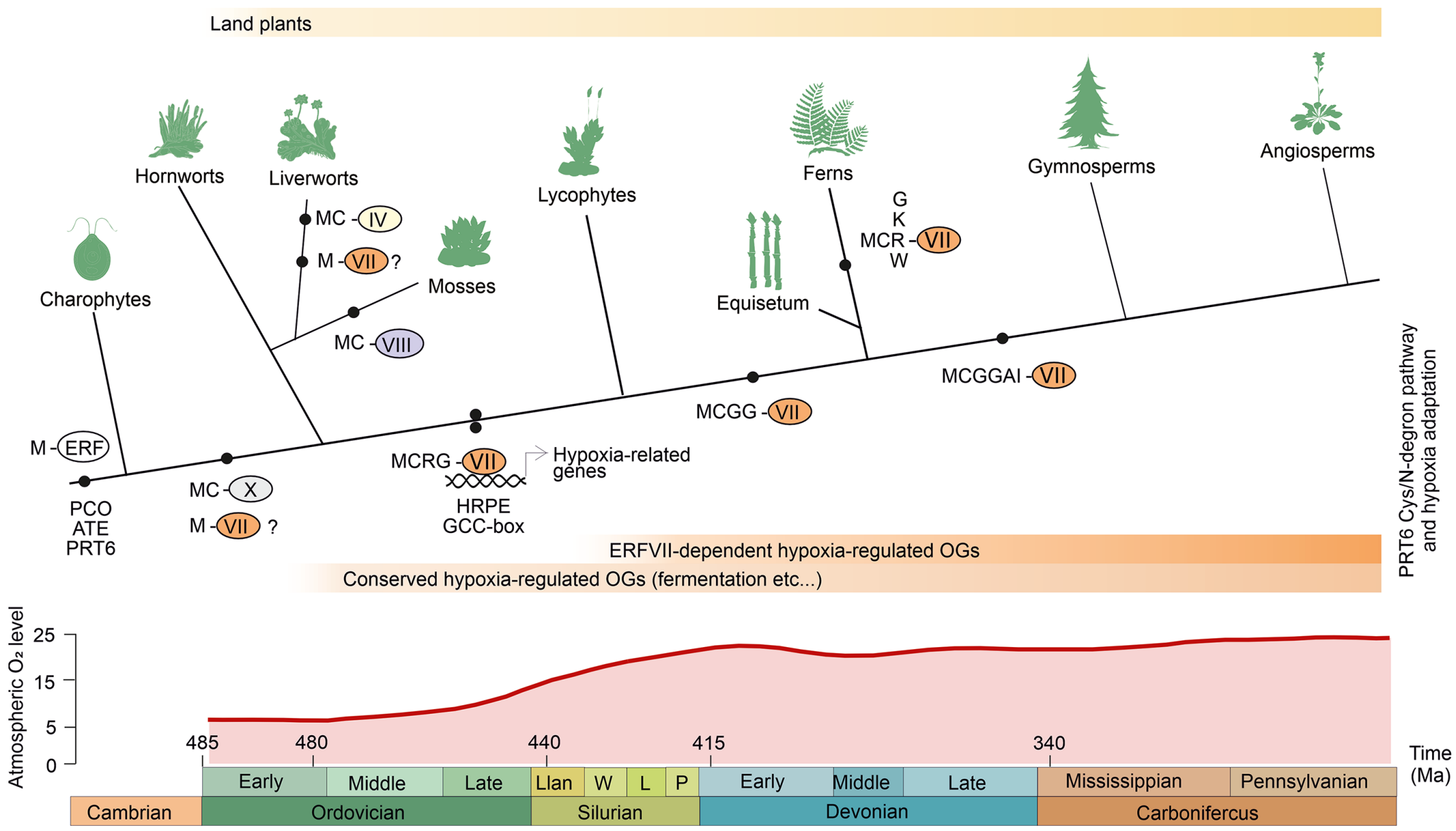

Figure 6. Evolution of Cys2-ERFs as transducers of hypoxia signaling in land plants.
The key enzymatic components of the PCO N-degron pathway (PCO, ATE, and PRT6) likely evolved in the last common ancestor of eukaryotes. Cys2-ERFs arose independently multiple times in land plants, with ERFVIIs evolving O_2-dependent degradability in vascular plants. In bryophytes, Cys2-ERF proteins belong to groups X, VII (mosses), and IV (liverworts). In ferns, the extended N-terminal sequence of ERFVIIs (the CMVII motif) shows greater variability compared with spermatophytes and lycophytes. ERFVII-like proteins are also found in liverworts, although these proteins lack the Cys residue at the second position. The recruitment of HRPE and GCC-box *cis-elements* in promoters of hypoxia-regulated genes is a feature specific to tracheophytes.

DISCUSSION

This study explored the conservation and diversification of the transcriptional response to hypoxia in land plants, as well as the evolution of ERFVII transcription factors, which serve as key transducers of hypoxic signaling in angiosperms. While the enzymatic components of the PCO N-degron pathway are ubiquitously distributed throughout the plant kingdom—inherited from the last common ancestor of eukaryotes (Holdsworth and Gibbs, 2020; Weits et al., 2023)—we found that the ERFVII substrate transducers are specific to tracheophytes, where they orchestrate gene expression changes under low oxygen conditions, suggesting that the evolution of ERFVIIs occurred at least 250 million years after the emergence of the metazoan hypoxia-inducible factor (Erwin et al., 2011; Mills et al., 2018). Our data indicate that different members of the ERF family were independently recruited as N-degron pathway substrates multiple times during land plant evolution, as seen in liverworts and mosses. However, it remains unclear whether these TFs contribute significantly to the transcriptional response to hypoxia when tested in *A. thaliana* (Figure 6). For instance, ABR1, a highly conserved Cys2-ERFX, appears to escape N-degron-mediated proteolysis in *A. thaliana*, where it regulates stress signaling and wound repair mechanisms (Baumler et al., 2019).

The orthogroups whose expression changed in response to reduced oxygen availability varied across species, as would be expected given their anatomical and ecological divergence. Nonetheless, a small set of induced orthogroups was conserved across all species investigated in this study (Figure 2B). These "core hypoxia-response orthogroups" include genes involved in metabolic reconfiguration toward glycolysis and fermentation, cell wall modification, and molecular transport, all of which were consistentlyupregulated (Figure 2B). Indeed, ethanol production has previously been identified as a highly conserved adaptation to low oxygen environments in plants (Bui et al., 2019). Moreover, many of the induced metabolic genes encode enzymes that utilize oxygen as a co-substrate, and therefore their induction likely represents a compensatory mechanism, as has been reported for the oxygen sensor PCO (Weits et al., 2023). Our analysis also identified a set of hypoxia-regulated orthogroups whose functions remain unknown due to a lack of similarity to known proteins. Further studies of these genes will be critical to fully elucidate the mechanisms underlying plant adaptation to hypoxia.

Our findings suggest the existence of an ancestral hypoxia signaling mechanism that is common to all land plants. Notably, *A. thaliana* is still able to activate a transcriptional response in the absence of ERFVIIs (Supplemental Figure 4). Further evidence for conserved signaling is provided by the induction of MYB and ERF orthologs in most of the species we examined. Interestingly, non-flowering species exhibited a reduced transcriptional response to hypoxia compared to angiosperms. This may reflect a lower sensitivity to hypoxic stress or, alternatively, the existence of non-transcriptional regulatory mechanisms. The stronger transcriptional response to hypoxia observed in *A. thaliana* and *O. sativa* (Figures 1 and 2A) suggests an expansion of the regulatory sphere of ERFVIIs beyond plant cell physiology, likely through the recruitment of

newly evolved and conserved orthogroups. Our phylogenetic analysis revealed a trend in which ERFs have been independently recruited as substrates of the PCO branch of the PRT6 N-degron pathway, with the evolution of Cys2-ERFs arising multiple times in different plant lineages. The conservation of Cys2 in ERFs outside angiosperms suggests its functional importance in environmental or developmental conditions that require PRT6 N-degron pathway-mediated proteolysis. When heterologously expressed in *A. thaliana*, non-vascular Cys2-ERFs were stabilized by NO scavenging, but not by hypoxia or submergence (Figure 4C). This finding confirms previous reports that PCO-mediated cysteine oxidation is independent of cellular NO levels, and further suggests that NO is required for steps downstream of PCO in the PRT6 N-degron pathway (Puerta et al., 2019; Gibbs and Holdsworth, 2020). Consequently, Cys2-ERFs from non-vascular species, as well as ERFVII-like proteins from vascular plants, may specifically regulate environmental or developmental conditions that decrease NO availability, a mechanism already demonstrated in flowering plants (Vicente et al., 2017, 2019). For example, NO synthesis via nitrate reductase is reduced under drought and high salinity stress, and shoot apical meristem establishment is responsive to lower NO levels (Gibbs et al., 2014; Vicente et al., 2017; Zeng et al., 2023).

Our data also revealed that ERFVIIs are present exclusively as Cys2-containing proteins—and thus as substrates of the PCO N-degron pathway—in vascular plants (Figure 6) (Weits et al., 2023). Their N-terminal consensus sequence (CMVII motif), which is absent from non-vascular plant ERFs, exhibits strong sequence conservation beyond Cys2 (Figure 3B). This motif may represent an optimized sequence for interaction with N-degron pathway enzymes or with other components of the hypoxia-responsive transcriptional machinery in tracheophytes. Supporting this notion, the stability of MpERF23 was not increased by hypoxia in *Arabidopsis* (Figure 4C). Instead, MpPCO was shown to act on the N-degron of both MpERF23 and AtRAP2.12 *in vitro* (Taylor-Kearney et al., 2022), with the latter able to confer protein stabilization under hypoxia in *M. polymorpha* (Figure 4D).

Importantly, the presence of ERFVIIs in vascular plants correlates with the recruitment of genes that constitute a core transcriptional response previously identified in angiosperms (Supplemental Tables 1–5) (Mustroph et al., 2010). When tested in *A. thaliana* as a heterologous host, non-spermatophyte ERFVIIs were unable to restore the rapid induction of low oxygen-responsive genes under submergence (Figure 5A), which we attribute to the lack of complementation for division-specific components of the transcriptional machinery activated under these conditions. However, this limitation was apparently overcome in the case of persistent, genetic inactivation of the N-degron pathway (the *erfVII prt6* background) (Figure 5B). Fusion of the angiosperm activation domain of RAP2.12 (Bui et al., 2015) to Cys2-ERFs from non-vascular clades led to significant induction of a hypoxia-responsive reporter in protoplasts (Figure 5D). However, the precise role of bryophyte Cys2-ERFs in their species of origin has yet to be thoroughly characterized. Moreover, transient transformation of *A. thaliana* protoplasts with the *S. moellendorffii* ERFVII and hypoxia-responsive promoters indicated conservation of both *cis*- and *trans*-regulatory components (Figure 5E). We therefore conclude that ERFVIIs likely acquired their role as transducers of the hypoxia response in the last common ancestor of vascular plants. The presence of similar ERFs in liverworts—which lack bothCys2 and the CMVII motif—suggests that they may have originated from an earlier ancestor in non-vascular plants but were subsequentlylost in sister lineages (Supplemental Table 34; Figure 6). However, the existence of several unique motifs in ERFVIIs points to a *de novo* origin and functional diversification of these transcription factors in the evolutionary transition from non-vascular to vascular plants.

Although non-vascular plants do not possess hypoxia-stabilized ERFVIIs, they are nontheless capable of adjusting gene expression in response to hypoxia. A direct oxygen-sensing mechanism could be responsible for the transcriptional response in bryophytes. This might involve a non-ERF N-terminal Cys-containing transcriptional regulator that is converted to a substrate of the PCO N-degron pathway by endoproteolysis. Alternatively, bryophytes may rely on a distinct biochemical strategy for direct oxygen sensing, such as 2-oxoglutarate-dependent dioxygenases, similar to those identified in the animal kingdom (Ivan et al., 2001; Batie et al., 2019; Chakraborty et al., 2019). The induction of O_2-consuming enzymes in *M. polymorpha* and *P. patens* suggests the existence of a feedback mechanism in which O_2-sensing enzymes themselves are hypoxia inducible, as is the case for PCOs in plants and PHDs in metazoans (Weits et al., 2014; D'Angelo et al., 2003). Less direct O_2-sensing mechanisms could also contribute to gene induction under hypoxia, involving variations in reactive oxygen species levels, cytosolic Ca^{2+} concentrations, and changes in lipid saturation that have been shown to play roles in low O_2 signaling in *A. thaliana* (Pucciariello and Perata, 2017; Schmidt et al., 2018; Fan et al., 2023).

The ERFVIIs are the most ancient PCO N-degron substrates identified to date. They are ubiquitous and highly conserved across all vascular plants. In contrast, VERNALIZATION 2 (VRN2) and LITTLE ZIPPER 2 (ZPR2) are angiosperm-specific, suggesting that their roles are derived and specific to this lineage (Gibbs et al., 2018; Labandera et al., 2021). It remains puzzling why ERFVIIs evolved specifically in vascular plants. A recent report (Zubrycka et al., 2023) demonstrated that, in *A. thaliana*, ERFVIIs are essential for maintaining an extensive root system in waterlogged soils and contribute to root growth under normal conditions. This indicates a critical requirement for ERFVII function in root development and survival in soil. Notably, complex soils first emerged in terrestrial environments at the time of the first appearance of plants with true roots (Spencer et al., 2022). An important property of soils is their capacity to undergo changes in water saturation levels. We speculate that ERFVIIs may have evolved to ensure root survival, a developmental innovation closely associated with plant vascularization (Hetherington and Dolan, 2018). This feature could have been essential for soil exploration under fluctuating water saturation levels and may have contributed to the successful colonization of diverse terrestrial habitats.

METHODS

Plant material and growth conditions

Marchantia polymorpha thalli or gemmae (accession Cambridge-2; Cam-2), *Selaginella moellendorffii*, and *Pteris vittata* shoots were cultivated

in vitro on half-strength MS medium (Murashige and Skoog, 1962) supplemented with 0.5% (w/v) sucrose and 0.8% (w/v) plant agar. *Physcomitrium patens* (kindly provided by Prof. Tomas Morosinotto, University of Padua) was grown on Knop medium (Reski and Abel, 1985). *M. polymorpha* thalli were subcultured from single gemmae, whereas *P. patens*, *S. moellendorffii*, and *P. vittata* plantlets were propagated from shoot cuttings. Plants were grown under controlled conditions: 50–60 μmol m^{-2} s^{-1} white light, a 16:8 h day:night photoperiod, and a temperature of 22°C. *Equisetum hyemale* plants were grown in soil (1:3 peat-free compost soil and vermiculite), under controlled environmental conditions (photoperiod 16:8 h, temperature 20:18°C light:dark), with white light supplemented by 10 μm far-red light.

The *A. thaliana erfVII* and *erfVII prt6* mutants were previously described (Abbas et al., 2015). The Col-0 accession was used as the wild-type reference. Transgenic Col-0 *A. thaliana* seeds carrying pUFT:RAP2.3^{3HA} were described previously (Zubrycka et al., 2023). *A. thaliana* seeds were sown in moist peat-free compost and vermiculite (1:3), stratified at 4°C in the dark for 48 h, and germinated at 22:18°C day:night under a 16:8 h photoperiod. For *in vitro* seedling cultivation, seeds were surface-sterilized, and sown on square plates containing half-strength MS medium supplemented with 1% (w/v) sucrose and 0.8% (w/v) plant agar. After cold treatment, plates were positioned vertically, and seeds were germinated under a 16:8 h day:night photoperiod at 22°C. The *M. polymorpha* transgenic line pEF1a:^{M50}AtRAP2.12-GFP was available in the laboratory.

Vector construction

The coding sequences (CDSs), starting at Cys^2 or C^{2}A, of MpERF23 (Mapoly0293s0001), PpERF133 (Pp3c7_1240V3.1), PpERF130 (Pp3c6_28370V3.1), SmERF1 (SELMODRAFT_73139), EhERF1 (scaffold-JVSZ-2009360), and PvERF1 were cloned from *M. polymorpha*, *P. patens*, *S. moellendorffii*, *E. hyemale*, and *P. vittata*, respectively, using Phusion High Fidelity DNA Polymerase (New England Biolabs). The amplified CDS fragments were cloned via *Sac*II digestion into the pCD1 vector (carrying the ^{FLAG}DHFR-UBIQUITIN-3xHA cassette) (Zubrycka et al., 2023) to generate entry vectors, which were then recombined individually into the plant transformation Gateway vector pH7WG2 using the LR reaction mix II (Life Technologies) to generate expression vectors, as described in Zubrycka et al. (2023). The *p35S:ccdB-RAP2.12-AD* vector was generated by assembling the Cauliflower mosaic virus (CaMV) 35S promoter, a synthesized minimal ccdB cassette, the activation domain of RAP2.12 (RAP2.12-AD, corresponding to 1021–1077 bp [351–358 aa]), and the 35S terminator into the pDGB3alpha2 backbone using the GoldenBraid cloning system (Sarrion-Perdigones et al., 2011). The full CDS of MpERF23, PpERF133, PpERF130, SmERF1, EhERF1, PvERF1, and RAP2.3 were cloned into the Gateway entry vector pDONR207 and then subcloned into the destination vector p35S:RAP2.12-AD via LR reaction. Primer sequences used to generate these constructs are listed in Supplemental Table 36.

Generation of transgenic plants

The DNA constructs described above were introduced into *erfVII* and *erfVII prt6 A. thaliana* mutants via *Agrobacterium tumefaciens*-mediated transformation using the floral dip method (Clough and Bent, 1998). Transgenic seeds were selected on agarized MS medium supplemented with hygromycin B (cat. no. 10687010, Thermo Fisher Scientific). Homozygous plants carrying a single transfer DNA insertion were identified by segregation analysis.

Identification of DNA motifs

Identification of Cys2-ERFs and ERFVIIs

ERFVII transcription factor sequences in tracheophytes (lycophytes, ferns, and gymnosperms) were identified by searching the ONEKP project database (https://db.cngb.org/onekp/) using the BLAST (tblastn) algorithm (Altschul et al., 1990), with the *A. thaliana* RAP2.12 (AT1G53910) protein sequence as a query. The retrieved protein sequences were then aligned against the *A. thaliana* protein database (www.phytozome.net) to confirm that they represent the closest orthologs. The best *A. thaliana* ERFVII orthologs from angiosperms, along with the five *Arabidopsis* ERFVIIs (AT1G53910, AT1G72360, AT2G47520, AT3G14230, and AT3G16770) ,were selected from those identified by Weits et al. (2023). Orthologous ERFVII sequences from lycophytes, ferns, gymnosperms, and angiosperms are listed in Supplemental Table 31. Identification of ERF transcription factor sequences that feature a Cys2 residue and an AP2 DNA binding domain in bryophytes and algae was performed as described above for tracheophyte sequences. ERF transcription factors from *C. reinhardtii*, *K. flaccidum*, *M. polymorpha*, *P. patens*, *S. moellendorffii*, and *A. thaliana* were retrieved from the PlantTFDB database (https://planttfdb.gao-lab.org/index.php) (Jin et al., 2017). *Anthoceros punctatus* ERF transcription factors were identified by BLAST search using orthologous ERF sequences from the hornwort *Anthoceros agrestis* (Li et al., 2020). Complete lists of the above-mentioned ERF sequences can be found in Supplemental Tables 32 and 33.

Phylogenetic analysis

Full-length ERFVII sequences retrieved from tracheophytes were aligned using the MUSCLE algorithm (Edgar, 2004) and then subjected to phylogenetic analysis. For ERF transcription factor sequences, only the aligned region corresponding to the AP2 binding domain was exported for subsequent analysis. Phylogenetic trees were constructed using the IQ-TREE online application (Trifinopoulos et al., 2016) with 1000 bootstrap replicates under either the VT + F + I + G4 or LG + G4 substitution models, as appropriate. Phylogenetic trees were visualized, annotated, and exported using the ''Interactive Tree of Life'' online tool (https://itol.embl.de) (Letunic and Bork, 2021).

Low oxygen, submergence, and chemical treatments

For hypoxia treatments, 13-day-old *M. polymorpha*, *P. patens*, *S. moellendorffii*, *P. vittata*, and *E. hyemale* plantlets were exposed to hypoxia (1% v/v O_2/N_2) for 8 h in the dark (from 8 a.m. to 4 p.m.), while control plants were kept in the dark under atmospheric conditions (21% ambient O_2). Each biological replicate consisted of three thalli (*M. polymorpha*), three young plantlets (*P. patens*), or three young shoots (*S. moellendorffii*, *E. hyemale*, and *P. vittata*). For western blot analysis or qRT-PCR, hypoxia and submergence treatments were performed on 7-day-old *A. thaliana* seedlings grown *in vitro*. These seedlings were treated with 0.1% v/v O_2/N_2 for 2 h in the dark or submerged in 2 mL Eppendorf tubes filled with deionized water for 1 h in the dark respectively. Chemical treatments were conducted on 7-day-old *in vitro Arabidopsis* seedlings with 50 μM bortezomib (CAS 179324-69-7, Santa Cruz Biotechnology), 100 μM cPTIO (cat. no. C221, Sigma-Aldrich), or DMSO (control), dissolved in deionized water, on an orbital shaker at 100 rpm for 3 and 2 h, respectively.

RNA extraction and qRT-PCR analysis

Total RNA was isolated from 80 to 100 mg of frozen and ground 13-day-old *M. polymorpha* thalli or young shoots of *S. moellendorffii*, *E. hyemale*, and *P. vittata* using the Spectrum Plant Total RNA Kit (Sigma-Aldrich) and the GeneJET Plant RNA Purification Kit (Thermo Fisher Scientific), according to the manufacturers' protocols. For 7-day-old *A. thaliana* seedlings, total RNA was extracted using the RNeasy Plant Mini Kit (QIAGEN) following the manufacturer's instructions. DNase treatment and cDNA synthesis were performed on 1 μg of RNA using the Maxima First Strand cDNA Synthesis Kit for RT-qPCR with dsDNase (Thermo Fisher Scientific). Quantitative real-time PCR (qRT-PCR) analysis was performed on *M. polymorpha*, *S. moellendorffii*, and *A. thaliana* RNA samples using the CFX384 Touch Real-Time PCR Detection System (Bio-Rad) and an SYBR Green PCR Master Mix (Life Technologies). Housekeeping genes *UBIQUITIN10* (*UBIQ10*, *At4g05320*), *Actin1* (*MpACT1*, *Mapoly0016s0137*), and *6-Phosphogluconate dehydrogenase* (*Sm6PGD*, *98 399*) were used as the endogenous controls for *A. thaliana*,

M. polymorpha, and *S. moellendorffii*, respectively. The primer sequences used for qRT-PCR analysis are listed in Supplemental Table 36. Relative gene expression levels were quantified using the comparative threshold cycle (ΔΔCt) method, as described in Livak and Schmittgen (2001). Two technical replicates were analyzed for each of four biological samples, and the data presented are representative of at least two independent experiments yielding comparable results.

RNA sequencing analysis

The RNA sequencing reads were first quality-trimmed by removing base calls with poor Phred scores (PHRED score > 30). Trimmed reads were then aligned using Kallisto (Bray et al., 2016) to the reference transcriptomes of the corresponding species for *M. polymorpha*, *P. patens*, and *S. moellendorffii*, or to *de novo* assembled transcriptomes for *E. hyemale* and *P. vittata*. *De novo* transcriptome assemblies were generated from quality-trimmed reads using the Trinity program (Grabherr et al., 2011) using a *k*-mer size of 31 and a minimum *k*-mer abundance of 2. Loci/genes with no sample exceeding 20 reads were excluded from further analysis. Library normalization for composition and size was performed using the TMM (trimmed mean of M values) approach (Robinson and Oshlack, 2010). Gene abundance, fold changes, and significance were calculated using a negative binomial model, with tagwise dispersion estimated via the "edgeR" R package (Robinson and Oshlack, 2010). *p* values were adjusted to a false discovery rate (FDR) of 0.05 using the Benjamini-Hochberg correction (Hochberg, 1995).

Seven-day-old *A. thaliana* seedlings of Col-0 and the *erfVII* mutant were treated with hypoxia (1% v/v O_2) or control conditions (21% ambient O_2), in the dark, starting at the end of the day, for 2 and 10 h. Total RNA was extracted using the GeneJET Plant RNA Purification Kit (Thermo Fisher Scientific), and genomic DNA was removed using the AMPure XP system (Beckman Coulter, Beverly, MA). Library quality was assessed using the Agilent Bioanalyzer 2100 system. Clustering of index-coded samples was performed on a cBot Cluster Generation System using the TruSeq PE Cluster Kit v3-cBot-HS (Illumina) according to the manufacturer's instructions. Libraries were sequenced on an Illumina NovaSeq 6000 platform to generate 150 bp paired-end reads. Paired-end clean reads were aligned to the *A. thaliana* TAIR10 reference genome (Ensembl Plants genome ID: 3702) using Hisat2 v2.0.5. Mapped reads for each sample were assembled with StringTie (v1.3.3b) (Pertea et al., 2015) in a reference-based approach. featureCounts v1.5.0-p3 (Liao et al., 2014) was used to count the number of reads mapped to each gene. Fragments per kilobase of transcript sequence per million base pairs sequenced for each gene was calculated based on gene length and mapped read counts. Differential expression analysis was performed using the DESeq2 R package (v1.20.0), with *p* values adjusted by the Benjamini-Hochberg method to control for FDR. Genes were considered differentially expressed when adjusted $p \leq 0.05$ and $|log_2FC| > 1$ (Supplemental Table 20).

In silico analysis

Gene expression profiles of the DEGs identified in the RNA-seq dataset ($log_2FC > 1$, $log_2FC < -1$ and $p < 0.05$) were compared across different experimental conditions by exploring public microarray data for *A. thaliana* under 1% v/v O_2 (Licausi et al., 2011) and *O. sativa* under anoxic conditions (Narsai et al., 2009), selecting DEGs based on $log_2FC > 1$, $log_2FC < -1$. and $p < 0.05$ (*A. thaliana*) or $p < 0.01$ (*O. sativa*).

Orthogroups were identified across 33 representative species of land plants using the OrthoFinder algorithm (Emms and Kelly, 2015). To facilitate the incorporation of *de novo* assembled transcriptomes, a discontiguous Megablast (Altschul et al., 1990) was used instead of the default Diamond alignment program for all-vs-all comparisons. The inflation parameter controlling the granularity of orthogroup clustering was set at 1.3 to maximize the number of conserved orthogroups.

Promoter binding sites were identified using established position-specific scoring matrices for HRPE, GCC, MDM, and group I and II R2R3-MYB binding sites (Supplemental Table 37). The FIMO tool was used to scan genome wide, examining 1000 bp upstream and 100 bp downstream of predicted transcription start sites. Sequences matching the position-specific scoring matrices with an E score of 10^{-4} or lower were considered authentic transcription factor binding sites. Enrichment of binding motifs was tested using Fisher's exact test.

SDS–PAGE and immunoblotting

Equal amounts of total protein (70 μg) were resolved by SDS–PAGE and transferred to a PVDF membrane using the Mini Trans-Blot electrophoretic transfer cell (Bio-Rad). Membranes were probed with an anti-HA primary antibody (Sigma-Aldrich, H3663) at a 1:2000 dilution, followed by an HRP-conjugated anti-mouse secondary antibody (Sigma-Aldrich, 12-349) at a 1:10 000 dilution. Immunoblots were developed on film using Pierce ECL Western Blotting Substrate (Thermo Fisher Scientific) or with SuperSignal West Pico PLUS Chemiluminescent Substrate (Thermo Fisher Scientific), and imaged on the iBright CL1500 Imaging System (Thermo Fisher Scientific).

Confocal imaging

M. polymorpha gemmae were used for probe detection following hypoxic treatment (1% v/v O_2/N_2) or control conditions (21% ambient O_2), for 6 h, in the dark. For propidium iodide (PI) staining, gemmae were incubated with 10 μg mL^{-1} of PI solution for 5–10 min, then rinsed with deionized water, and mounted on slides. Imaging was performed using a Zeiss LSM 880 microscope (Department of Plant Sciences, University of Oxford) equipped with a 25× objective lens. GFP, PI, and chlorophyll were excited at 488 nm, and emissions were collected at 495–560 nm (GFP), 590–635 nm (PI), and 650–750 nm (chlorophyll).

Luciferase transactivation assay in protoplasts

A. thaliana erfVII prt6 or Col-0 (wild-type) mesophyll protoplasts were isolated from leaves of 3-week-old plants and transfected as described by Yoo et al. (2007). Two micrograms of each plasmid were used to transfect 100 μL of protoplast suspension. After 16 h of incubation in the dark at 22°C, firefly (*Photinus pyralis*) luciferase activity was measured and normalized to the sea pansy (*Renilla reniformis*) luciferase signal using the Dual-Luciferase Reporter Assay System (Promega), according to the manufacturer's instructions. Each sample consisted of five independent replicates.

Statistical analysis

Statistical analyses were performed using GraphPad Prism version 10. Depending on the dataset, one-way or two-way ANOVAs were conducted, with differences between means considered significant at $p < 0.05$. For one-way ANOVA, multiple comparisons of means were performed using Tukey's method, whereas for two-way ANOVA, comparisons were made using the Holm-Sidak method.

DATA AVAILABILITY

All RNA sequencing raw data generated in this study can be accessed at the Sequence Read Archive at the National Center for Biotechnology Information under accession numbers PRJNA1128557 (accessible through the following link: https://www.ncbi.nlm.nih.gov/geo/query/acc.cgi?acc=GSE270814, token ybyniygoxxgjrwx) and PRJNA1150224 (accessible through the following link: https://dataview.ncbi.nlm.nih.gov/object/PRJNA1150224?reviewer=gtgp3k045jhk99v5p6i342iqgf).

FUNDING

This work was supported by a Leverhulme Trust Research Project Grant (RPG-2017-132) to M.J.H.; the European Research Council (ERC, grant agreement no. 101001320, "Synoxys"); the Italian Ministry of University and Research (MIUR, PRIN grant no. 20173EWRT9); and the Biotechnology and Biological Sciences Research Council (BB/X001059/1) to F.L.

ACKNOWLEDGMENTS
No conflict of interest declared.

AUTHOR CONTRIBUTIONS
M.J.H. and F.L. conceived the research. L.D.C., H.v.V., M.J.H., and F.L. designed the experiments and interpreted the data. All authors carried out the research. F.L., L.D.C., and M.J.H. wrote the manuscript. All authors read, contributed to editing, and approved the manuscript.

SUPPLEMENTAL INFORMATION
Supplemental information is available at *Molecular Plant Online*.

Received: October 31, 2024
Revised: March 31, 2025
Accepted: May 24, 2025
Published: May 28, 2025

REFERENCES
Abbas, M., Berckhan, S., Rooney, D.J., Gibbs, D.J., Vicente Conde, J., Sousa Correia, C., Bassel, G.W., Marín-De La Rosa, N., León, J., Alabadí, D., et al. (2015). Oxygen sensing coordinates photomorphogenesis to facilitate seedling survival. Curr. Biol. **25**:1483–1488. https://doi.org/10.1016/j.cub.2015.03.060.

Abbas, M., Sharma, G., Dambire, C., Marquez, J., Alonso-Blanco, C., Proaño, K., and Holdsworth, M.J. (2022). An oxygen-sensing mechanism for angiosperm adaptation to altitude. Nature **606**:565–569. https://doi.org/10.1038/s41586-022-04740-y.

Altschul, S.F., Gish, W., Miller, W., Myers, E.W., and Lipman, D.J. (1990). Basic local alignment search tool. J. Mol. Biol. **215**:403–410. https://doi.org/10.1016/S0022-2836(05)80360-2.

Batie, M., Frost, J., Frost, M., Wilson, J.W., Schofield, P., and Rocha, S. (2019). Hypoxia induces rapid changes to histone methylation and reprograms chromatin. Sci. Technol. Humanit. **363**:1222–1226. https://doi.org/10.1126/science.aau5870.

Baumler, J., Riber, W., Klecker, M., Muller, L., Dissmeyer, N., Weig, A. R., and Mustroph, A. (2019). AtERF#111/ABR1 is a transcriptional activator involved in the wounding response. Plant J. **100**:969–990. https://doi.org/10.1111/tpj.14490.

Bowman, J.L., Kohchi, T., Yamato, K.T., Jenkins, J., Shu, S., Ishizaki, K., Yamaoka, S., Nishihama, R., Nakamura, Y., Berger, F., et al. (2017). Insights into Land Plant Evolution Garnered from the Marchantia polymorpha Genome. Cell **171**:287–304.e15. https://doi.org/10.1016/j.cell.2017.09.030.

Bray, N.L., Pimentel, H., Melsted, P., and Pachter, L. (2016). Near-optimal probabilistic RNA-seq quantification. Nat. Biotechnol. **34**:525–527. https://doi.org/10.1038/nbt.3519.

Bui, L.T., Giuntoli, B., Kosmacz, M., Parlanti, S., and Licausi, F. (2015). Constitutively expressed ERF-VII transcription factors redundantly activate the core anaerobic response in Arabidopsis thaliana. Plant Sci. **236**:37–43. https://doi.org/10.1016/j.plantsci.2015.03.008.

Bui, L.T., Novi, G., Lombardi, L., Iannuzzi, C., Rossi, J., Santaniello, A., Mensuali, A., Corbineau, F., Giuntoli, B., Perata, P., et al. (2019). Conservation of ethanol fermentation and its regulation in land plants. J. Exp. Bot. **70**:1815–1827. https://doi.org/10.1093/jxb/erz052.

Chakraborty, A.A., Laukka, T., Myllykoski, M., Ringel, A.E., Booker, M. A., Tolstorukov, M.Y., Meng, Y.J., Meier, S.R., Jennings, R.B., Creech, A.L., et al. (2019). Histone demethylase KDM6A directly senses oxygen to control chromatin and cell fate. Sci. Technol. Humanit. **363**:1217–1222. https://doi.org/10.1126/science.aaw1026.

Clough, S.J., and Bent, A.F. (1998). Floral dip: A simplified method for Agrobacterium-mediated transformation of Arabidopsis thaliana. Plant J. **16**:735–743. https://doi.org/10.1046/j.1365-313X.1998.00343.x.

D'Angelo, G., Duplan, E., Boyer, N., Vigne, P., and Frelin, C. (2003). Hypoxia up-regulates prolyl hydroxylase activity: a feedback mechanism that limits HIF-1 responses during reoxygenation. J. Biol. Chem. **278**:38183–38187. https://doi.org/10.1074/jbc.M302244200.

De Clercq, I., Vermeirssen, V., Van Aken, O., Vandepoele, K., Murcha, M.W., Law, S.R., Inzé, A., Ng, S., Ivanova, A., Rombaut, D., et al. (2013). The membrane-bound NAC transcription factor ANAC013 functions in mitochondrial retrograde regulation of the oxidative stress response in Arabidopsis. Plant Cell **25**:3472–3490. https://doi.org/10.1105/tpc.113.117168.

Donoghue, P.C.J., Harrison, C.J., Paps, J., and Schneider, H. (2021). The evolutionary emergence of land plants. Curr. Biol. **31**:R1281–R1298. https://doi.org/10.1016/j.cub.2021.07.038.

Edgar, R.C. (2004). MUSCLE: Multiple sequence alignment with high accuracy and high throughput. Nucleic Acids Res. **32**:1792–1797. https://doi.org/10.1093/nar/gkh340.

Emms, D.M., and Kelly, S. (2015). OrthoFinder: solving fundamental biases in whole genome comparisons dramatically improves orthogroup inference accuracy. Genome Biol. **16**:157. https://doi.org/10.1186/s13059-015-0721-2.

Erwin, D.H., Laflamme, M., Tweedt, S.M., Sperling, E.A., Pisani, D., and Peterson, K.J. (2011). The Cambrian conundrum: early divergence and later ecological success in the early history of animals. Sci. Technol. Humanit. **334**:1091–1097. https://doi.org/10.1126/science.1206375.

Fan, B., Liao, K., Wang, L.N., Shi, L.L., Zhang, Y., Xu, L.J., Zhou, Y., Li, J.F., Chen, Y.Q., Chen, Q.F., et al. (2023). Calcium-dependent activation of CPK12 facilitates its cytoplasm-to-nucleus translocation to potentiate plant hypoxia sensing by phosphorylating ERF-VII transcription factors. Mol. Plant **16**:979–998. https://doi.org/10.1016/j.molp.2023.04.002.

Gasch, P., Fundinger, M., Müller, J.T., Lee, T., Bailey-Serres, J., and Mustroph, A. (2016). Redundant ERF-VII transcription factors bind an evolutionarily-conserved cis-motif to regulate hypoxia-responsive gene expression in Arabidopsis. Plant Cell **28**:160–180. https://doi.org/10.1105/tpc.15.00866.

Gibbs, D.J., and Holdsworth, M.J. (2020). Every Breath You Take: New Insights into Plant and Animal Oxygen Sensing. Cell **180**:22–24. https://doi.org/10.1016/j.cell.2019.10.043.

Gibbs, D.J., Lee, S.C., Isa, N.M., Gramuglia, S., Fukao, T., Bassel, G. W., Correia, C.S., Corbineau, F., Theodoulou, F.L., Bailey-serres, J., et al. (2011). Homeostatic response to hypoxia is regulated by N-end rule pathway in plants. Nature **479**:415–418. https://doi.org/10.1038/nature10534.

Gibbs, D.J., Mdlsa, N., Movahedi, M., Lozano-Juste, J., Mendiondo, G.M., Berckhan, S., Marín-delaRosa, N., VicenteConde, J., SousaCorreia, C., Pearce, S.P., et al. (2014). Nitric Oxide Sensing in Plants Is Mediated by Proteolytic Control of Group VII ERF Transcription Factors. Mol. Cell **53**:369–379. https://doi.org/10.1016/j.molcel.2013.12.020.

Gibbs, D.J., Tedds, H.M., Labandera, A.M., Bailey, M., White, M.D., Hartman, S., Sprigg, C., Mogg, S.L., Osborne, R., Dambire, C., et al. (2018). Oxygen-dependent proteolysis regulates the stability of angiosperm polycomb repressive complex 2 subunit VERNALIZATION 211. Nat. Commun. **9**:5438. https://doi.org/10.1038/s41467-018-07875-7.

Giuntoli, B., Shukla, V., Maggiorelli, F., Giorgi, F.M., Lombardi, L., Perata, P., and Licausi, F. (2017). Age-dependent regulation of ERF-VII transcription factor activity in Arabidopsis thaliana. Plant Cell Environ. **40**:2333–2346. https://doi.org/10.1111/pce.13037.

Grabherr, M.G., Haas, B.J., Yassour, M., Levin, J.Z., Thompson, D.A., Amit, I., Adiconis, X., Fan, L., Raychowdhury, R., Zeng, Q., et al. (2011). Full-length transcriptome assembly from RNA-Seq data

without a reference genome. Nat. Biotechnol. **29**:644–652. https://doi.org/10.1038/nbt.1883.

Hammarlund, E.U., Flashman, E., Mohlin, S., and Licausi, F. (2020). Oxygen-sensing mechanisms across eukaryotic kingdoms and their roles in complex multicellularity. Sci. Technol. Humanit. **370**: eaba3512. https://doi.org/10.1126/science.aba3512.

Hao, D., Ohme-Takagi, M., and Sarai, A. (1998). Unique mode of GCC box recognition by the DNA-binding domain of ethylene-responsive element-binding factor (ERF domain) in plant. J. Biol. Chem. **273**:26857–26861. https://doi.org/10.1074/jbc.273.41.26857.

Hetherington, A.J., and Dolan, L. (2018). Stepwise and independent origins of roots among land plants. Nature **561**:235–238. https://doi.org/10.1038/s41586-018-0445-z.

Hochberg, B. (1995). Controlling the False Discovery Rate: a Practical and Powerful Approach to Multiple Testing. J. R. Stat. **57**:289–300. https://doi.org/10.2307/2346101.

Holdsworth, M.J., and Gibbs, D.J. (2020). Comparative Biology of Oxygen Sensing in Plants and Animals. Curr. Biol. **30**:R362–R369. https://doi.org/10.1016/j.cub.2020.03.021.

Ivan, M., Kondo, K., Yang, H., Kim, W., Valiando, J., Ohh, M., Salic, A., Asara, J.M., Lane, W.S., and Kaelin, W.G., Jr. (2001). HIFalpha targeted for VHL-mediated destruction by proline hydroxylation: implications for O2 sensing. Sci. Technol. Humanit. **292**:464–468. https://doi.org/10.1126/science.1059817.

Jin, J., Tian, F., Yang, D.C., Meng, Y.Q., Kong, L., Luo, J., and Gao, G. (2017). PlantTFDB 4.0: toward a central hub for transcription factors and regulatory interactions in plants. Nucleic Acids Res. **45**:D1040–D1045. https://doi.org/10.1093/nar/gkw982.

Kelemen, Z., Sebastian, A., Xu, W., Grain, D., Salsac, F., Avon, A., Berger, N., Tran, J., Dubreucq, B., Lurin, C., et al. (2015). Analysis of the DNA-Binding Activities of the Arabidopsis R2R3-MYB Transcription Factor Family by One-Hybrid Experiments in Yeast. PLoS One **10**:e0141044. https://doi.org/10.1371/journal.pone.0141044.

Labandera, A.M., Tedds, H.M., Bailey, M., Sprigg, C., Etherington, R. D., Akintewe, O., Kalleechurn, G., Holdsworth, M.J., and Gibbs, D.J. (2021). The PRT6 N-degron pathway restricts VERNALIZATION 2 to endogenous hypoxic niches to modulate plant development. New Phytol. **229**:126–139. https://doi.org/10.1111/nph.16477.

Letunic, I., and Bork, P. (2021). Interactive Tree Of Life (iTOL) v5: an online tool for phylogenetic tree display and annotation. Nucleic Acids Res. **49**:W293–W296. https://doi.org/10.1093/nar/gkab301.

Li, F.W., Nishiyama, T., Waller, M., Frangedakis, E., Keller, J., Li, Z., Fernandez-Pozo, N., Barker, M.S., Bennett, T., Blázquez, M.A., et al. (2020). Anthoceros genomes illuminate the origin of land plants and the unique biology of hornworts. Nat. Plants **6**:259–272. https://doi.org/10.1038/s41477-020-0618-2.

Liao, Y., Smyth, G.K., and Shi, W. (2014). featureCounts: an efficient general purpose program for assigning sequence reads to genomic features. Bioinformatics **30**:923–930. https://doi.org/10.1093/bioinformatics/btt656.

Licausi, F., Kosmacz, M., Weits, D.a., Giuntoli, B., Giorgi, F.M., Voesenek, L.a.C.J., Perata, P., and van Dongen, J.T. (2011). Oxygen sensing in plants is mediated by an N-end rule pathway for protein destabilization. Nature **479**:419–422. https://doi.org/10.1038/nature10536.

Livak, K.J., and Schmittgen, T.D. (2001). Analysis of relative gene expression data using real-time quantitative PCR and the 2-ΔΔCT method. Methods **25**:402–408. https://doi.org/10.1006/meth.2001.1262.

Mills, D.B., Francis, W.R., Vargas, S., Larsen, M., Elemans, C.P., Canfield, D.E., and Wörheide, G. (2018). The last common ancestor of animals lacked the HIF pathway and respired in low-oxygen environments. eLife **7**:e31176. https://doi.org/10.7554/eLife.31176.

Morris, J.L., Puttick, M.N., Clark, J.W., Edwards, D., Kenrick, P., Pressel, S., Wellman, C.H., Yang, Z., Schneider, H., and Donoghue, P.C.J. (2018). The timescale of early land plant evolution. Proc. Natl. Acad. Sci. USA **115**:E2274–E2283. https://doi.org/10.1073/pnas.1719588115.

Murashige, T., and Skoog, F. (1962). A Revised Medium for Rapid Growth and Bio Assays with Tobacco Tissue Cultures. Physiol. Plantarum **15**:473–497. https://doi.org/10.1111/j.1399-3054.1962.tb08052.x.

Mustroph, A., Lee, S.C., Oosumi, T., Zanetti, M.E., Yang, H., Ma, K., Yaghoubi-Masihi, A., Fukao, T., and Bailey-Serres, J. (2010). Cross-Kingdom Comparison of Transcriptomic Adjustments to Low-Oxygen Stress Highlights Conserved and Plant-Specific Responses. Plant Physiol. **152**:1484–1500. https://doi.org/10.1104/pp.109.151845.

Mustroph, A., Zanetti, M.E., Jang, C.J.H., Holtan, H.E., Repetti, P.P., Galbraith, D.W., Girke, T., and Bailey-Serres, J. (2009). Profiling translatomes of discrete cell populations resolves altered cellular priorities during hypoxia in Arabidopsis. Proc. Natl. Acad. Sci. USA **106**:18843–18848. https://doi.org/10.1073/pnas.0906131106.

Nakano, T., Suzuki, K., Fujimura, T., and Shinshi, H. (2006). Genome-Wide Analysis of the ERF Gene Family. Plant Physiol. **140**:411–432. https://doi.org/10.1104/pp.105.073783.

Narsai, R., Howell, K.A., Carroll, A., Ivanova, A., Millar, A.H., and Whelan, J. (2009). Defining Core Metabolic and Transcriptomic Responses to Oxygen Availability in Rice Embryos and Young Seedlings. Plant Physiol. **151**:306–322. https://doi.org/10.1104/pp.109.142026.

Pertea, M., Pertea, G.M., Antonescu, C.M., Chang, T.C., Mendell, J.T., and Salzberg, S.L. (2015). StringTie enables improved reconstruction of a transcriptome from RNA-seq reads. Nat. Biotechnol. **33**:290–295. https://doi.org/10.1038/nbt.3122.

Pucciariello, C., and Perata, P. (2017). New insights into reactive oxygen species and nitric oxide signalling under low oxygen in plants. Plant Cell Environ. **40**:473–482. https://doi.org/10.1111/pce.12715.

Puerta, M.L., Shukla, V., Dalle Carbonare, L., Weits, D.A., Perata, P., Licausi, F., and Giuntoli, B. (2019). A Ratiometric Sensor Based on Plant N-Terminal Degrons Able to Report Oxygen Dynamics in Saccharomyces cerevisiae. J. Mol. Biol. **431**:2810–2820. https://doi.org/10.1016/j.jmb.2019.05.023.

Reski, R., and Abel, W.O. (1985). Induction of budding on chloronemata and caulonemata of the moss, Physcomitrella patens, using isopentenyladenine. Planta **165**:354–358. https://doi.org/10.1007/BF00392232.

Robinson, M.D., and Oshlack, A. (2010). A scaling normalization method for differential expression analysis of RNA-seq data. Genome Biol. **11**: R25–R29. https://doi.org/10.1186/gb-2010-11-3-r25.

Sarrion-Perdigones, A., Falconi, E.E., Zandalinas, S.I., Juárez, P., Fernández-del-Carmen, A., Granell, A., and Orzaez, D. (2011). GoldenBraid: an iterative cloning system for standardized assembly of reusable genetic modules. PLoS One **6**:e21622. https://doi.org/10.1371/journal.pone.0021622.

Schmidt, R.R., Fulda, M., Paul, M.V., Anders, M., Plum, F., Weits, D.A., Kosmacz, M., Larson, T.R., Graham, I.A., Beemster, G.T.S., et al. (2018). Low-oxygen response is triggered by an ATP-dependent shift in oleoyl-CoA in Arabidopsis. Proc. Natl. Acad. Sci. USA **115**: E12101–E12110. https://doi.org/10.1073/pnas.1809429115.

Song, B., Modjewski, L.D., Kapust, N., Mizrahi, I., and Martin, W.F. (2022). The origin and distribution of the main oxygen sensing

mechanism across metazoans. Front. Physiol. **13**:977391. https://doi.org/10.3389/fphys.2022.977391.

Spencer, C.J., Davies, N.S., Gernon, T.M., Wang, X., McMahon, W.J., Morrell, T.R.I., Hincks, T., Pufahl, P.K., Brasier, A., Seraine, M., et al. (2022). Composition of continental crust altered by the emergence of land plants. Nat. Geosci. **15**:735–740. https://doi.org/10.1038/s41561-022-00995-2.

Taylor-Kearney, L.J., Madden, S., Wilson, J., Myers, W.K., Gunawardana, D.M., Pires, E., Holdship, P., Tumber, A., Rickaby, R.E.M., and Flashman, E. (2022). Plant Cysteine Oxidase Oxygen-Sensing Function Is Conserved in Early Land Plants and Algae. ACS Bio. Med. Chem. Au **2**:521–528. https://doi.org/10.1021/acsbiomedchemau.2c00032.

Trifinopoulos, J., Nguyen, L.T., von Haeseler, A., and Minh, B.Q. (2016). W-IQ-TREE: a fast online phylogenetic tool for maximum likelihood analysis. Nucleic Acids Res. **44**:W232–W235. https://doi.org/10.1093/nar/gkw256.

van Dongen, J.T., and Licausi, F. (2015). Oxygen Sensing and Signaling. Annu. Rev. Plant Biol. **66**:345–367. https://doi.org/10.1146/annurev-arplant-043014-114813.

Varshavsky, A. (2011). The N-end rule pathway and regulation by proteolysis. Protein Sci. **20**:1298–1345. https://doi.org/10.1002/pro.666.

Vicente, J., Mendiondo, G.M., Movahedi, M., Peirats-Llobet, M., Juan, Y.t., Shen, Y.y., Dambire, C., Smart, K., Rodriguez, P.L., Charng, Y. y., et al. (2017). The Cys-Arg/N-End Rule Pathway Is a General Sensor of Abiotic Stress in Flowering Plants. Curr. Biol. **27**:3183–3190.e4. https://doi.org/10.1016/j.cub.2017.09.006.

Vicente, J., Mendiondo, G.M., Pauwels, J., Pastor, V., Izquierdo, Y., Naumann, C., Movahedi, M., Rooney, D., Gibbs, D.J., Smart, K., et al. (2019). Distinct branches of the N-end rule pathway modulate the plant immune response. New Phytol. **221**:988–1000. https://doi.org/10.1111/nph.15387.

Voesenek, L.A.C.J., and Bailey-Serres, J. (2015). Flood adaptive traits and processes: an overview. New Phytol. **206**:57–73. https://doi.org/10.1111/nph.13209.

Wapinski, I., Pfeffer, A., Friedman, N., and Regev, A. (2007). Automatic genome-wide reconstruction of phylogenetic gene trees. Bioinformatics **23**:i549–i558. https://doi.org/10.1093/bioinformatics/btm193.

Weits, D.A., Giuntoli, B., Kosmacz, M., Parlanti, S., Hubberten, H.M., Riegler, H., Hoefgen, R., Perata, P., Van Dongen, J.T., and Licausi, F. (2014). Plant cysteine oxidases control the oxygen-dependent branch of the N-end-rule pathway10. Nat. Commun. **5**:3425. https://doi.org/10.1038/ncomms4425.

Weits, D.A., Kunkowska, A.B., Kamps, N.C.W., Portz, K.M.S., Packbier, N.K., Nemec Venza, Z., Gaillochet, C., Lohmann, J.U., Pedersen, O., van Dongen, J.T., et al. (2019). An apical hypoxic niche sets the pace of shoot meristem activity. Nature **569**:714–717. https://doi.org/10.1038/s41586-019-1203-6.

Weits, D.A., Zhou, L., Giuntoli, B., Carbonare, L.D., Iacopino, S., Piccinini, L., Lombardi, L., Shukla, V., Bui, L.T., Novi, G., et al. (2023). Acquisition of hypoxia inducibility by oxygen sensing N-terminal cysteine oxidase in spermatophytes. Plant Cell Environ. **46**:322–338. https://doi.org/10.1111/pce.14440.

White, M.D., Klecker, M., Hopkinson, R.J., Weits, D.A., Mueller, C., Naumann, C., O'Neill, R., Wickens, J., Yang, J., Brooks-Bartlett, J.C., et al. (2017). Plant cysteine oxidases are dioxygenases that directly enable arginyl transferase-catalysed arginylation of N-end rule targets. Nat. Commun. **8**:14690. https://doi.org/10.1038/ncomms14690.

Xu, K., Xu, X., Fukao, T., Canlas, P., Maghirang-Rodriguez, R., Heuer, S., Ismail, A.M., Bailey-Serres, J., Ronald, P.C., and Mackill, D.J. (2006). Sub1A is an ethylene-response-factor-like gene that confers submergence tolerance to rice. Nature **442**:705–708. https://doi.org/10.1038/nature04920.

Yoo, S.-D., Cho, Y.-H., and Sheen, J. (2007). Arabidopsis mesophyll protoplasts: a versatile cell system for transient gene expression analysis. Nat. Protoc. **2**:1565–1572. https://doi.org/10.1038/nprot.2007.199.

Zeng, J., Zhao, X., Liang, Z., Hidalgo, I., Gebert, M., Fan, P., Wenzl, C., Gornik, S.G., and Lohmann, J.U. (2023). Nitric oxide controls shoot meristem activity via regulation of DNA methylation. Nat. Commun. **14**:8001. https://doi.org/10.1038/s41467-023-43705-1.

Zhang, H., Rundle, C., Winter, N., Miricescu, A., Mooney, B.C., Bachmair, A., Graciet, E., and Theodoulou, F.L. (2024). BIG enhances Arg/N-degron pathway-mediated protein degradation to regulate Arabidopsis hypoxia responses and suberin deposition. Plant Cell **36**:3177–3200. https://doi.org/10.1093/plcell/koae117.

Zubrycka, A., Dambire, C., Dalle Carbonare, L., Sharma, G., Boeckx, T., Swarup, K., Sturrock, C.J., Atkinson, B.S., Swarup, R., Corbineau, F., et al. (2023). ERFVII action and modulation through oxygen-sensing in Arabidopsis thaliana. Nat. Commun. **14**:4665. https://doi.org/10.1038/s41467-023-40366-y.

Molecular Plant, Volume *18*

Supplemental information

ERFVIIs as transducers of oxygen-sensing in the evolution of land plant response to hypoxia

Laura Dalle Carbonare, Hans van Veen, Vinay Shukla, Monica Perri, Liem Bui, Michael J. Holdsworth, and Francesco Licausi

Supplemental Information

ERFVIIs as transducers of oxygen-sensing in the evolution of land plant response to hypoxia

Laura Dalle Carbonare 1,2, Hans van Veen 3, Vinay Shukla 1, Monica Perri 1, Liem Bui 4, Michael J. Holdsworth* 1 and Francesco Licausi* 2

Affiliations

1 School of Biosciences, University of Nottingham, Loughborough, LE12 5RD, UK

2 Department of Biology, University of Oxford, Oxford, OX1 3RB, UK

3 Groningen Institute of Evolutionary Life Sciences, University of Groningen, Groningen, NL

4 Biotechnology Research and Development Institute, Can Tho University, Can Tho, Vietnam

Contact information: Michael J. Holdsworth (michael.holdsworth@nottingham.ac.uk) and Francesco Licausi (francesco.licausi@biology.ox.ac.uk)

Additional footnotes: *Corresponding authors, equal contribution

Supplemental Figures

A

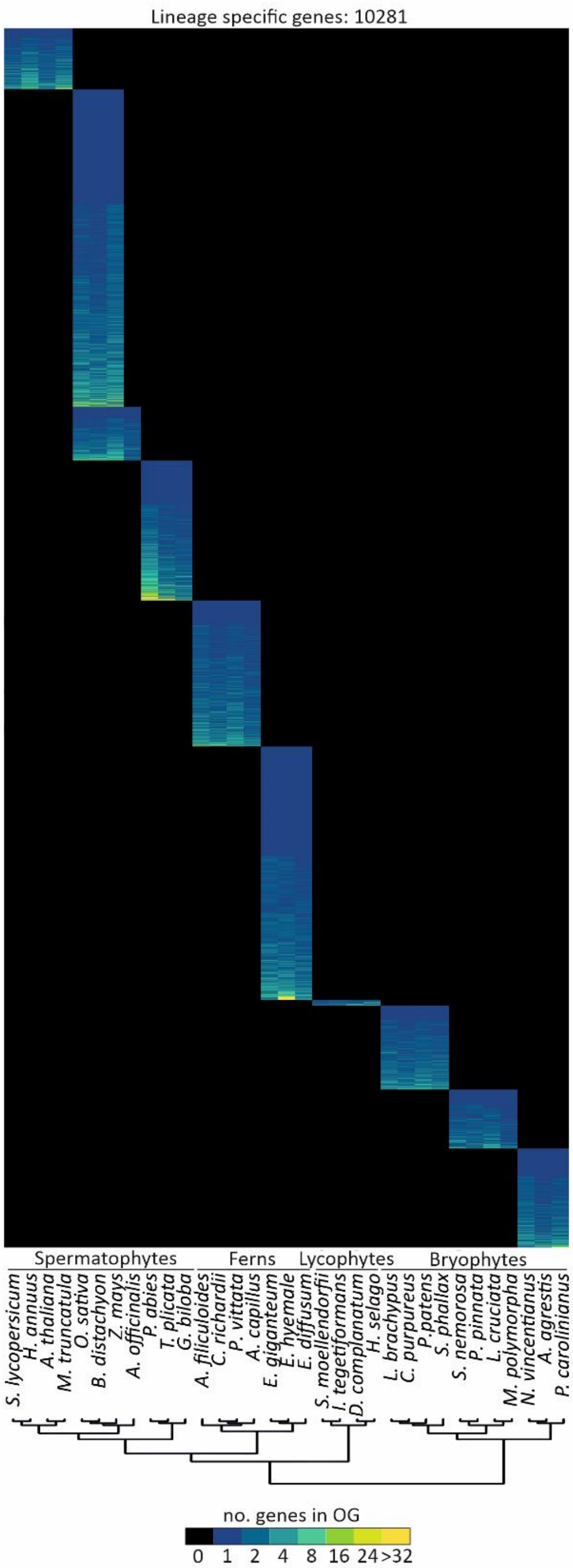

B

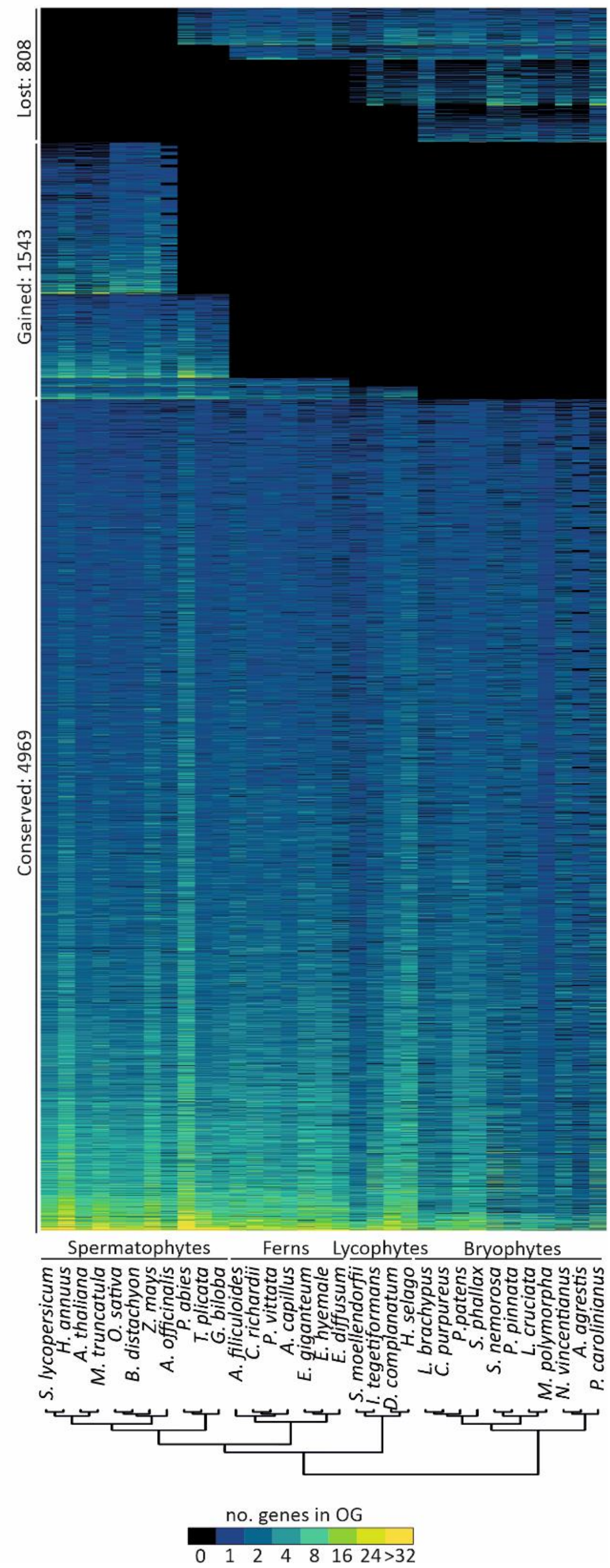

Supplemental Figure S1. **Distribution of genes across land plant evolution**. **A**) Hierarchical clustering of lineage specific genes (10281) from 33 land plant species, including those studied in this work: *M. polymorpha*, *P. patens*, *S. moellendorfii*, *E. hyemale*, *P. vittata*, *O. sativa* and *A. thaliana*. **B**) Conserved (4969), gained (1543) and lost (808) orthogroups from 33 land plant species.

A

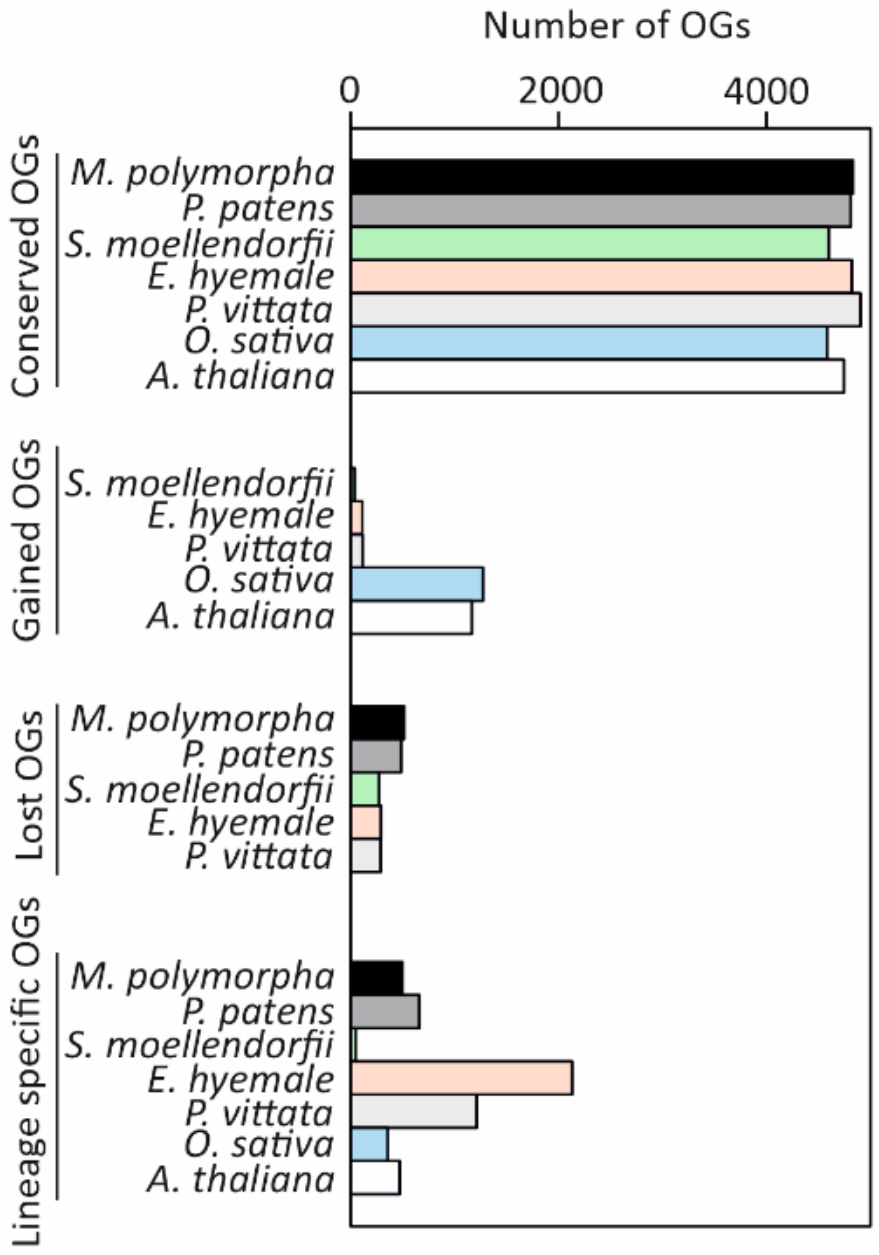

B

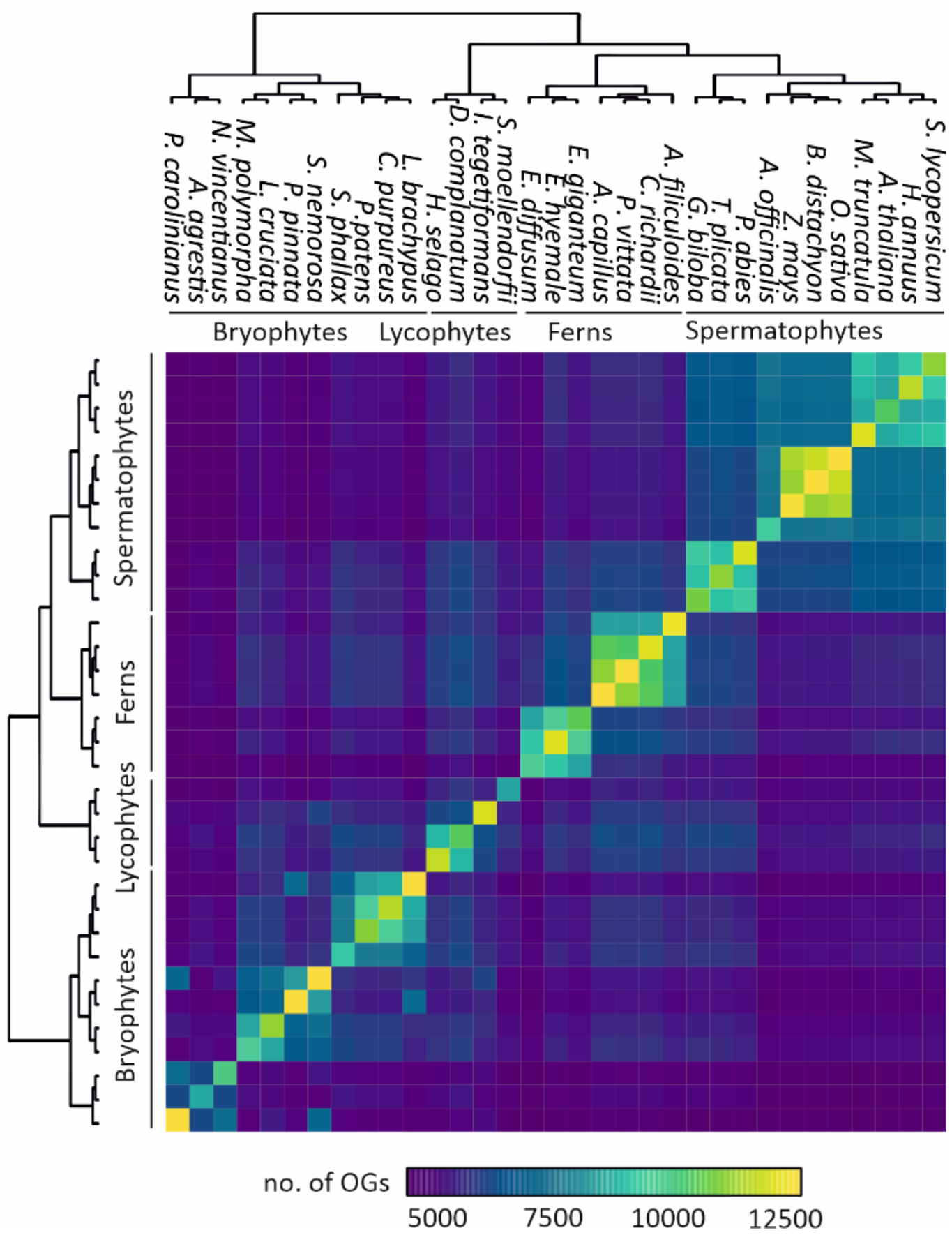

Supplemental Figure S2. **Classification of orthogroups across land plant evolution**. **A**) Bar plots depicting the number of conserved, gained, lost and lineage specific orthogroups in *M. polymorpha*, *P. patens*, *S. moellendorfii*, *E. hyemale*, *P. vittata*, *O. sativa* and *A. thaliana*. **B**) The amount of orthogroups shared between pairs of species.

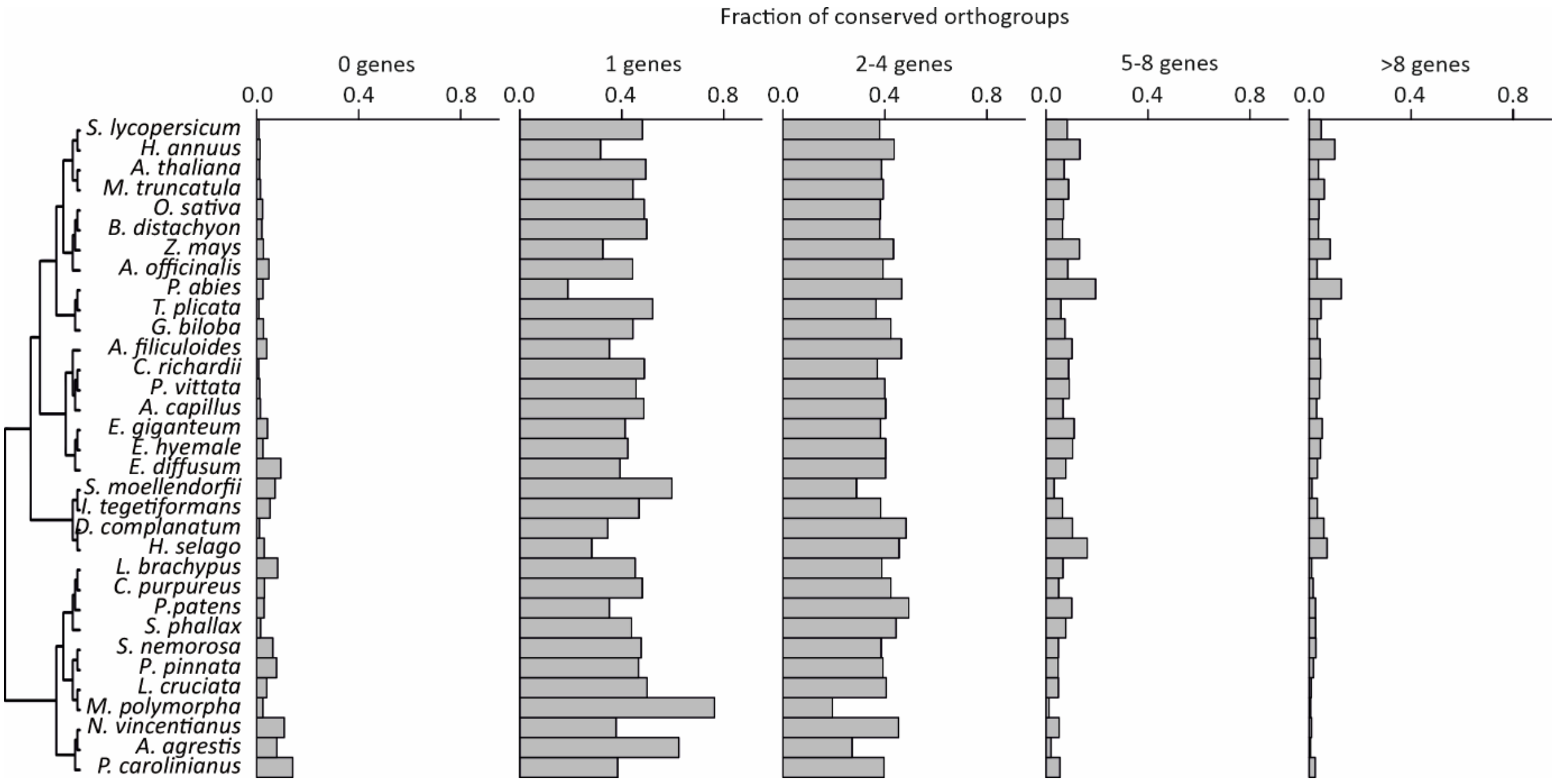

Supplemental Figure S3. Distribution of number of genes in conserved orthogroups. Percentage of number of genes, over the total number of genes, included in each of the fraction of conserved orthogroups identified for the 33 land plant species analysed in this study. Fractions of conserved orthogroups are classified according to the number of genes they contain (0, 1, 2-4, 5-8 and more than 8 genes).

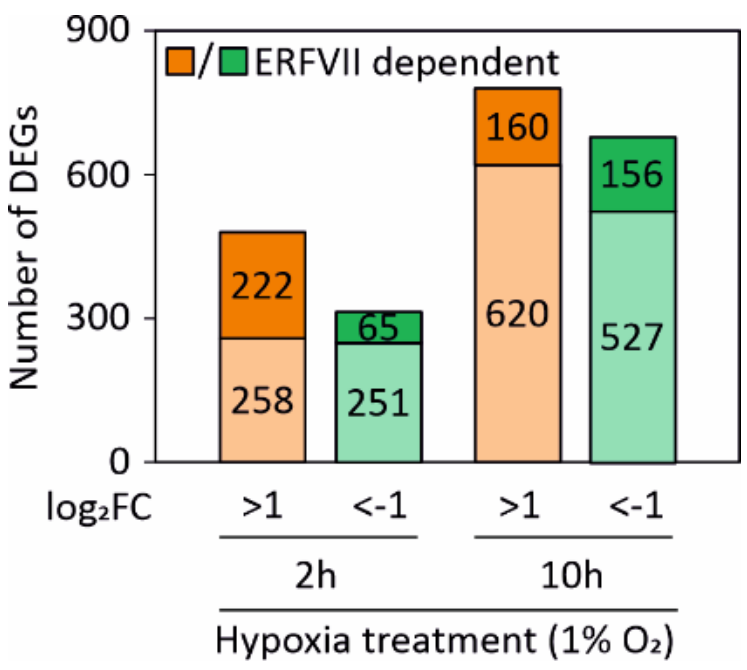

Supplemental Figure S4. Contribution of the ERFVIIs to the hypoxia response in *A. thaliana*. Bar plots representing the number of differentially expressed genes (DEGs) in *A. thaliana* following two or ten hours of dark hypoxia treatment (1% v/v O_2) in 7-day-old seedlings of WT (Col-0) plants compared to the *erfVII* mutant. Light orange and light green represent upregulated ($log_2FC>1$, $FDR<0.05$) or downregulated ($log_2FC<-1$, $FDR<0.05$) genes, respectively. Dark orange and dark green represent ERFVII-dependent upregulated ($log_2FC>1$, $FDR<0.05$) or downregulated ($log_2FC<-1$, $FDR<0.05$) genes, respectively. The exact number of genes for each category is highlighted inside each bar.

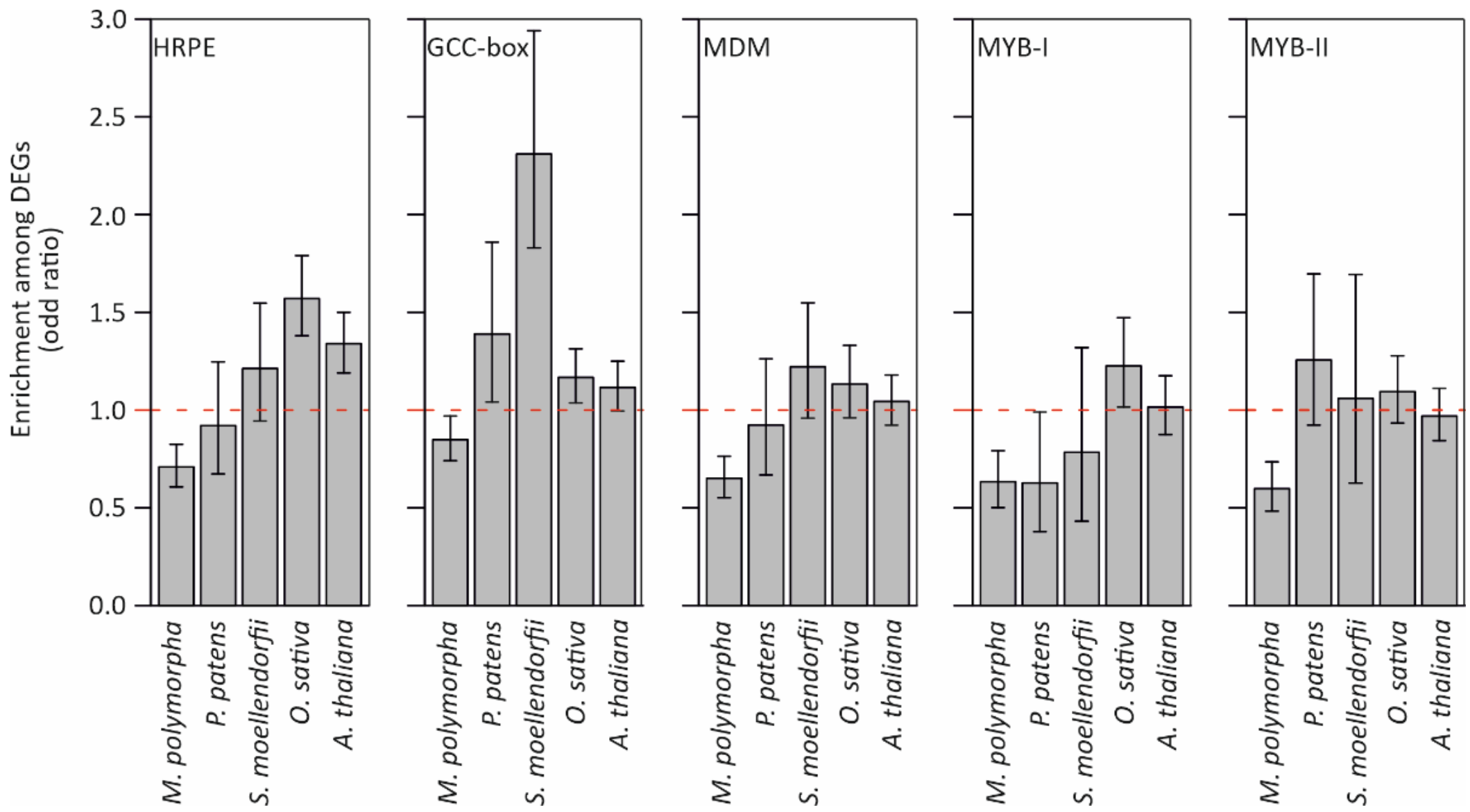

Supplemental Figure S5. **Motifs enrichment upon hypoxia in land plants**. Enrichment of *cis*-element motifs (HRPE, GCC-box, MDM, MYB-I and MYB-II) among differentially upregulated genes (P<0.05 and $|log_2FC|>1$). Odds ratio of 1 indicates no enrichment, whereas higher values are enriched. Error bars represent the 95% confidence intervals (Fisher's exact test).

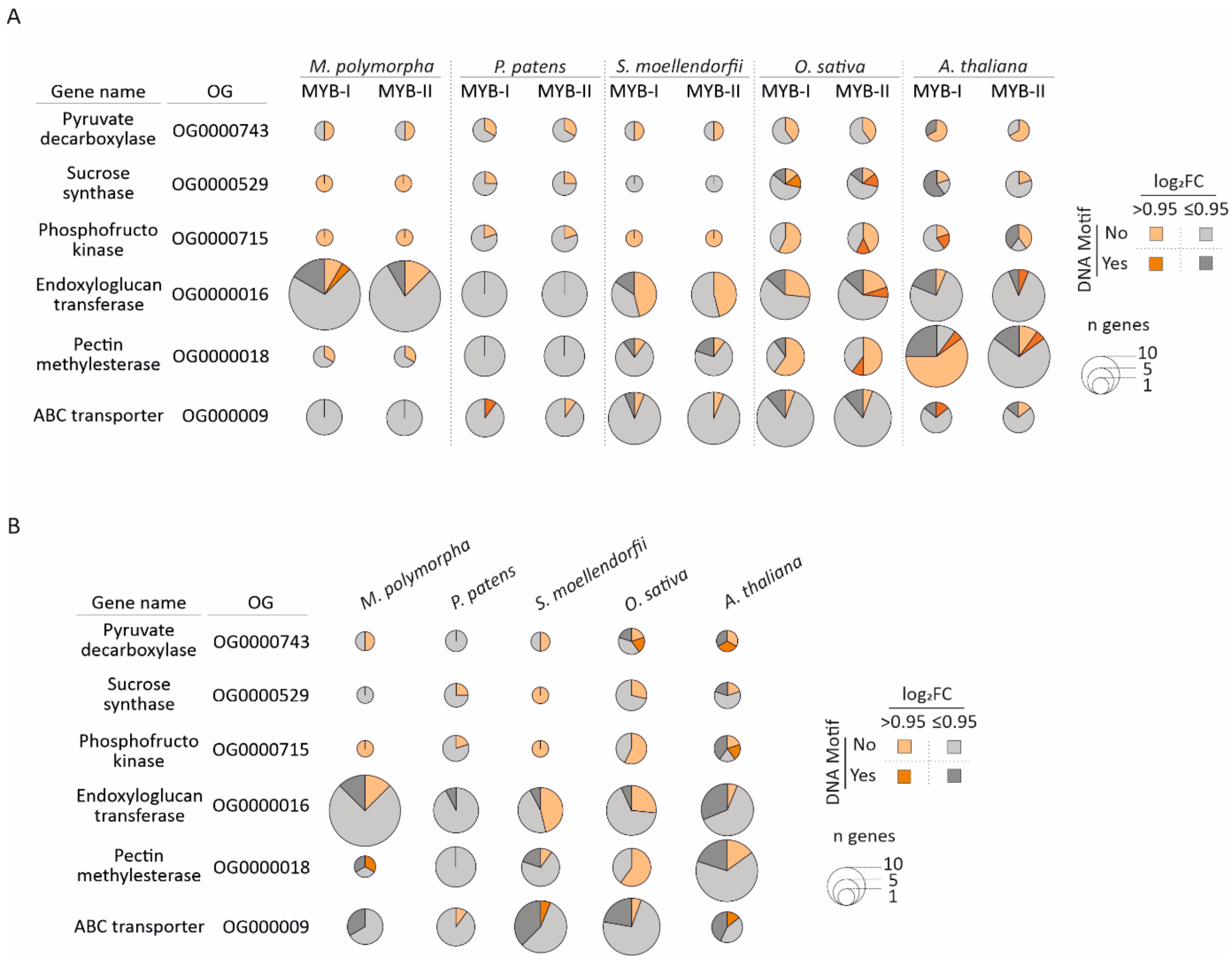

Supplemental Figure S6. **Frequency of MYB and NAC *cis*-element motifs in hypoxia-induced promoters across land plants**. **A**) Pie charts representing the occurrence of MYB-binding motifs (MYB-I and MYB-II) or **B**) NAC-binding motif (MDM) in promoters of upregulated (dark orange, $log_2FC>0.95$) and downregulated (dark grey, $log_2FC \leq 0.95$) genes belonging to the seven hypoxia-induced and conserved orthogroups in *M. polymorpha*, *P. patens*, *S. moellendorfii*, *E. hyemale*, *P. vittata*, *O. sativa* and *A. thaliana*. The size of each pie chart is proportional to the number of genes belonging to the orthogroups for each individual species, with total upregulated genes in light orange ($log_2FC>0.95$) and total downregulated genes in light grey ($log_2FC \leq 0.95$).

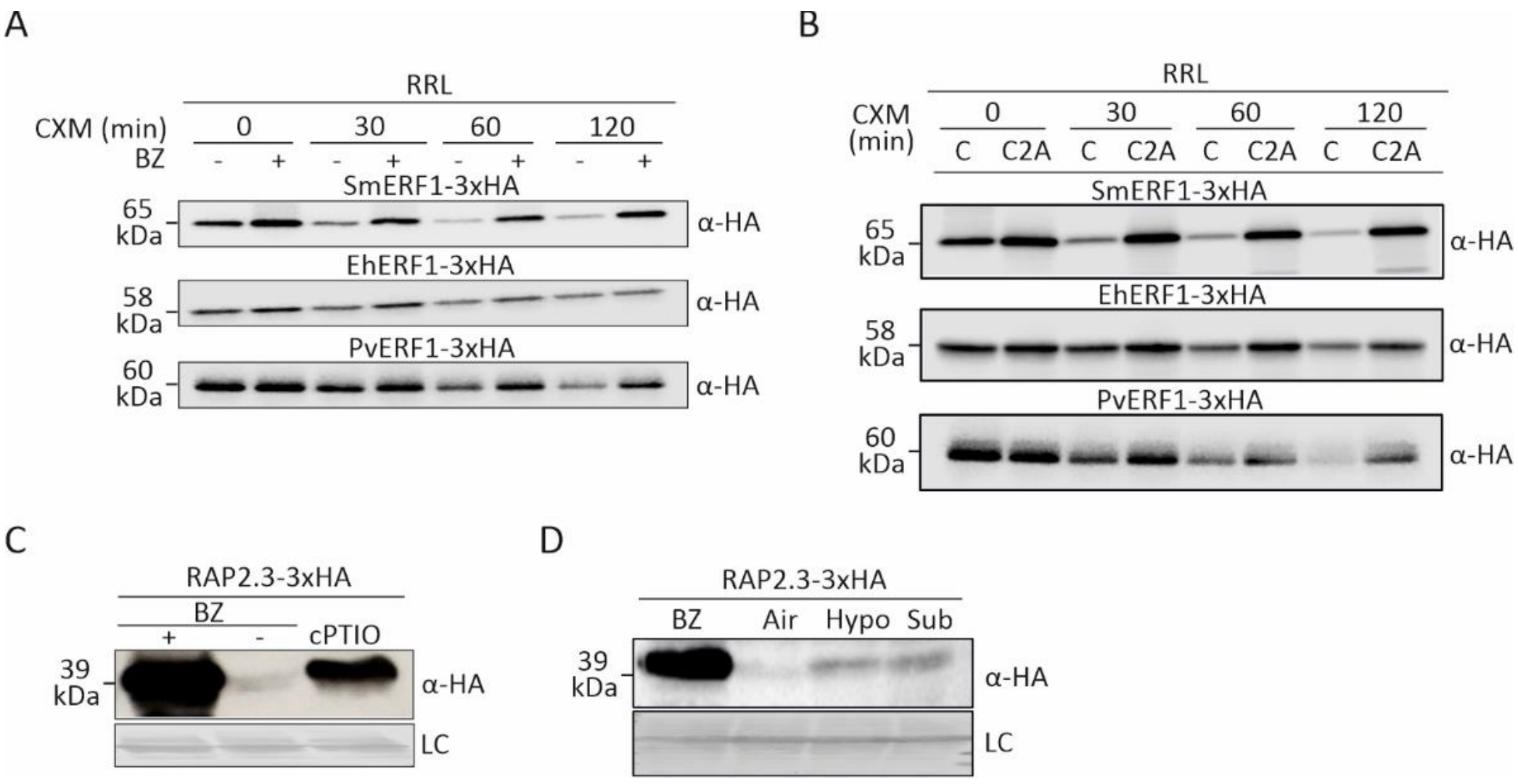

Supplemental Figure S7. **Tracheophyte ERFVIIs regulation through the PCO branch of the PRT6 N-degron pathway**. **A**) Western blot analysis of $SmERF1^{3xHA}$, $EhERF1^{3xHA}$ and $PvERF1^{3xHA}$ abundance in rabbit reticulocyte lysate (RRL) in control (DMSO) or bortezomib (BZ) conditions during a time course (0, 30, 60 and 120 min) after cycloheximide (CXM) addition. **B**) Western blot analysis of Cys2 compared to C2A $SmERF1^{3xHA}$, $EhERF1^{3xHA}$ and $PvERF1^{3xHA}$ abundance in RRL during a time course (0, 30, 60 and 120 min) after cycloheximide (CXM) treatment. **C**) Western blot analysis of $RAP2.3^{3xHA}$ abundance in control (DMSO), bortezomib (BZ) or NO-scavenger (cPTIO) treated *erfVII* mutant seedlings. **D**) Western blot analysis of $RAP2.3^{3xHA}$ abundance in bortezomib (BZ), air (21% v/v O_2), hypoxia (2h, 0.1% v/v O_2) or submergence (1h) treated *erfVII* mutant seedlings. Loading control (LC) corresponds to a re-fitted and compacted image of the membrane after Ponceau staining. The unedited images are shown in **Supplemental Fig. S16**.

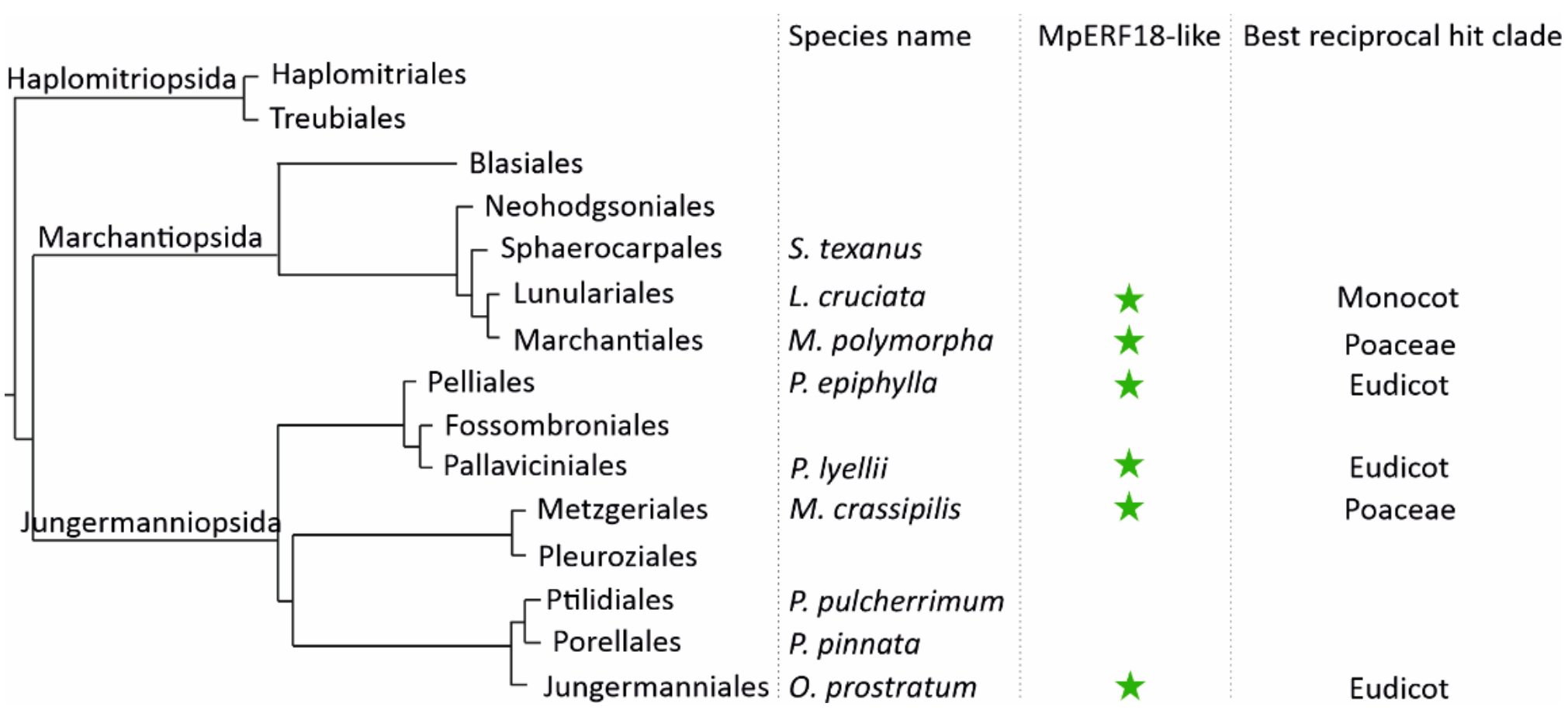

Supplemental Figure S8. **Occurrence of *M. polymorpha* MpERF18-like proteins in liverworts**. tblastn algorithm search of the 1KP database using *M. polymorpha* MpERF18 (Mapoly0092s0058) protein sequence as query. The presence of orthologues of MpERF18 are marked with a green star, together with the species name where they have been identified. For each MpERF18 orthologs found, their best reciprocal hit clade is as well reported.

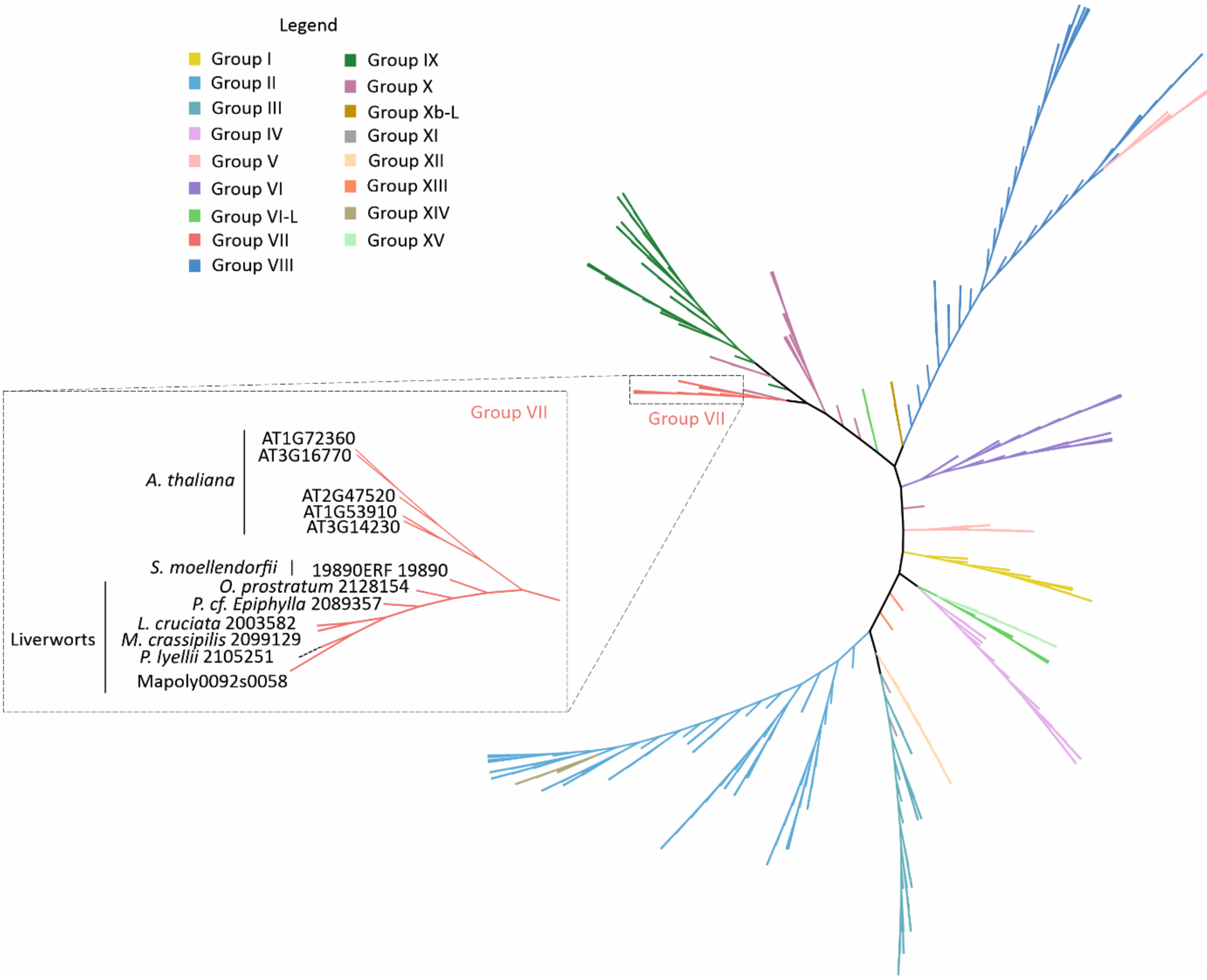

Supplemental Figure S9. **Phylogenetic relationship between ERF proteins in bryophytes, *S. moellendorfii* and *A. thaliana*.** Unrooted phylogenetic tree of ERF proteins from bryophytes (*A. punctatus*, *M. polymorpha* and *P. patens*), lycophyte (*S. moellendorfii*) and angiosperm (*A. thaliana*), including orthologous ERFVII-like proteins of MpERF18 retrieved from other groups of liverworts, which are shown in **Supplemental Fig. S8** and listed in **Supplemental Table 34**. ERFs classification is based on (Nakano et al., 2006). In the dashed box is a magnification of the Group VII branch, where the names of the sequences at each node and the species or clade to which they belong are highlighted.

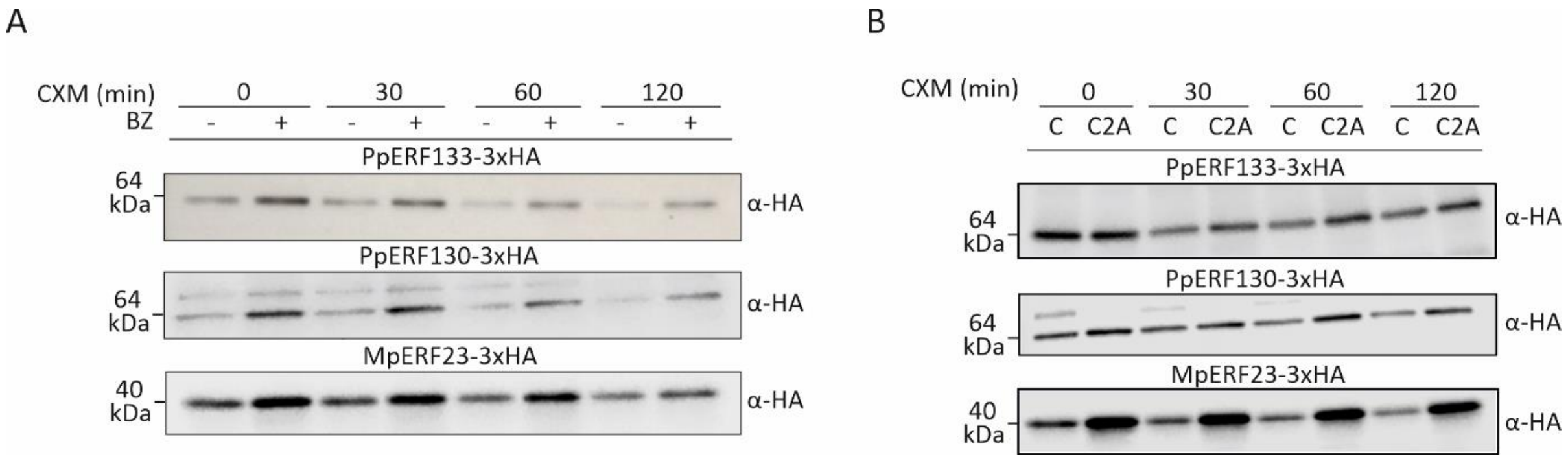

Supplemental Figure S10. **Bryophyte ERF regulation through the N-degron pathway. A**) Western blot analysis of ERF Cys2 proteins $PpERF133^{3xHA}$, $PpERF130^{3xHA}$ and $MpERF23^{3xHA}$ abundance in rabbit reticulocyte lysate (RRL) in control (DMSO) or bortezomib (BZ) conditions during a time course (0, 30, 60 and 120 min) after cycloheximide (CXM) treatment. **B**) Western blot analysis of Cys2 compared to C2A $PpERF133^{3xHA}$, $PpERF130^{3xHA}$ and $MpERF23^{3xHA}$ abundance in RRL during a time course (0, 30, 60 and 120 min) after cycloheximide (CXM) treatment.

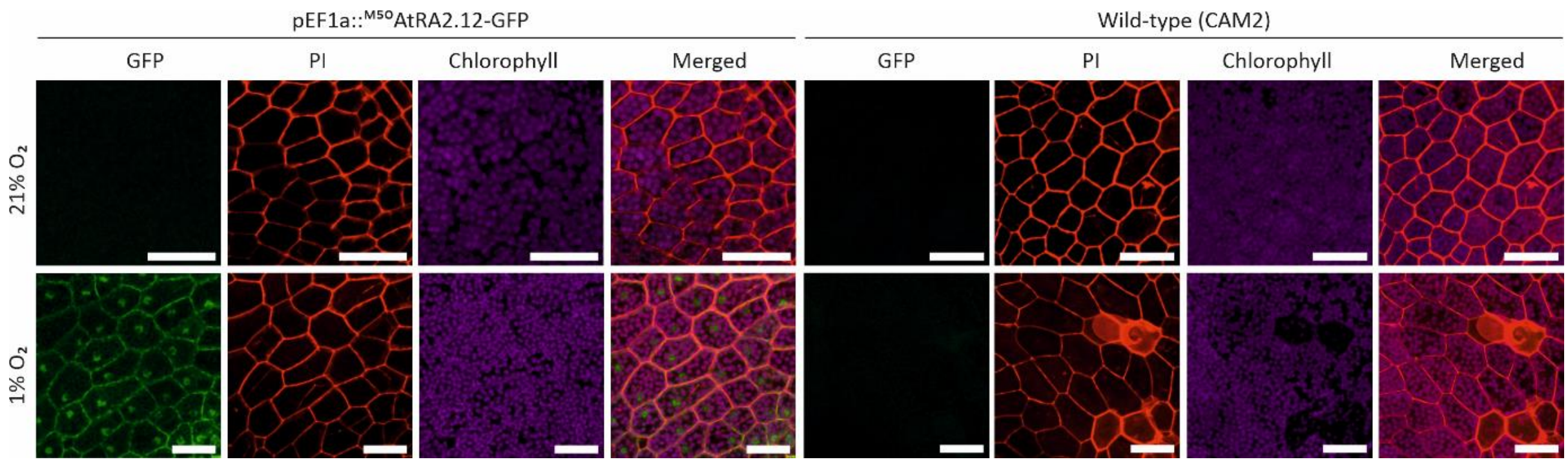

Supplemental Figure S11. ***A. thaliana* RAP2.12 stability depends on hypoxia in *M. polymorpha***. Confocal images of the subcellular localization of a reporter consisting of the first 50 aa of AtRAP2.12 fused to Citrine, overexpressed in *M. polymorpha* 13-day-old thalli in air (21% ambient O_2) and hypoxia (1% v/v O_2), compared to wild-type (CAM2) plants.

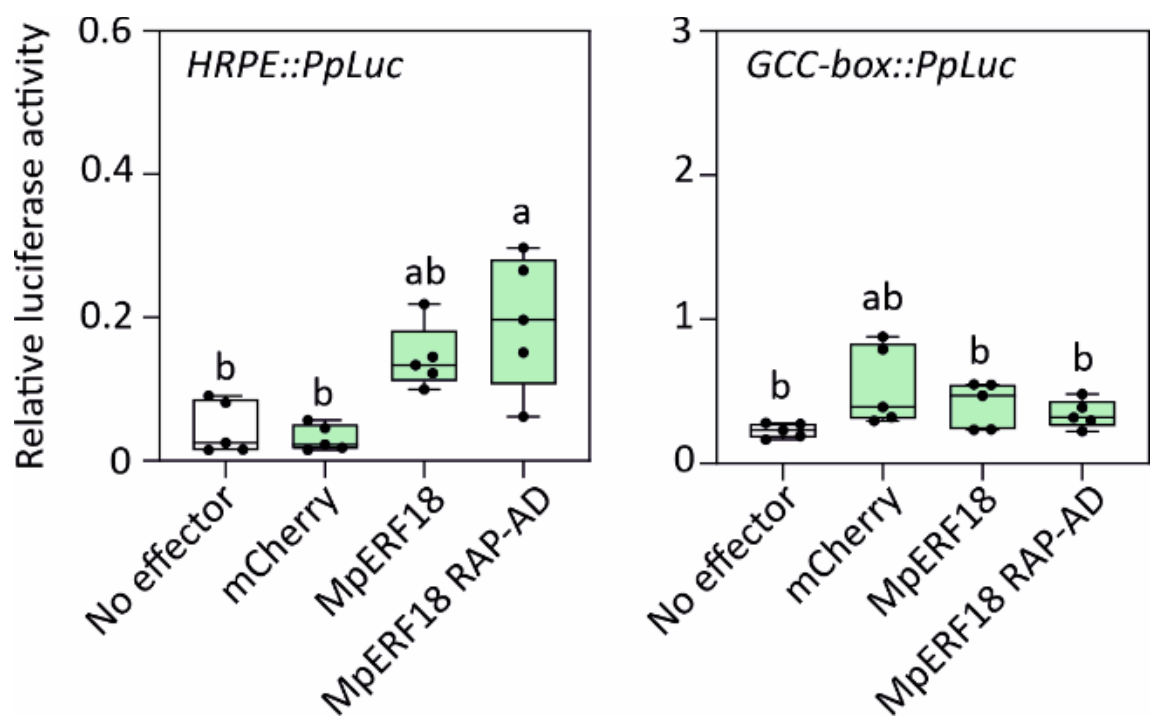

Supplemental Figure S12. **MpERF18 transactivation efficiency on hypoxia-related promoters**. Effect of *M. polymorpha* ERFVII-like (MpERF18) wild type or fused at the C-terminal to RAP2.12 activation domain (MpERF18 RAP-AD) on the synthetic promoters HRPE (left) and GCC-box (right), measured as relative firefly luciferase activity in *A. thaliana* wild-type mesophyll protoplasts. Data are presented as means ± SD (n=5). Different letters indicate statistically different averages as assessed by one-way ANOVA followed by the Tukey's posthoc test ($P < 0.05$).

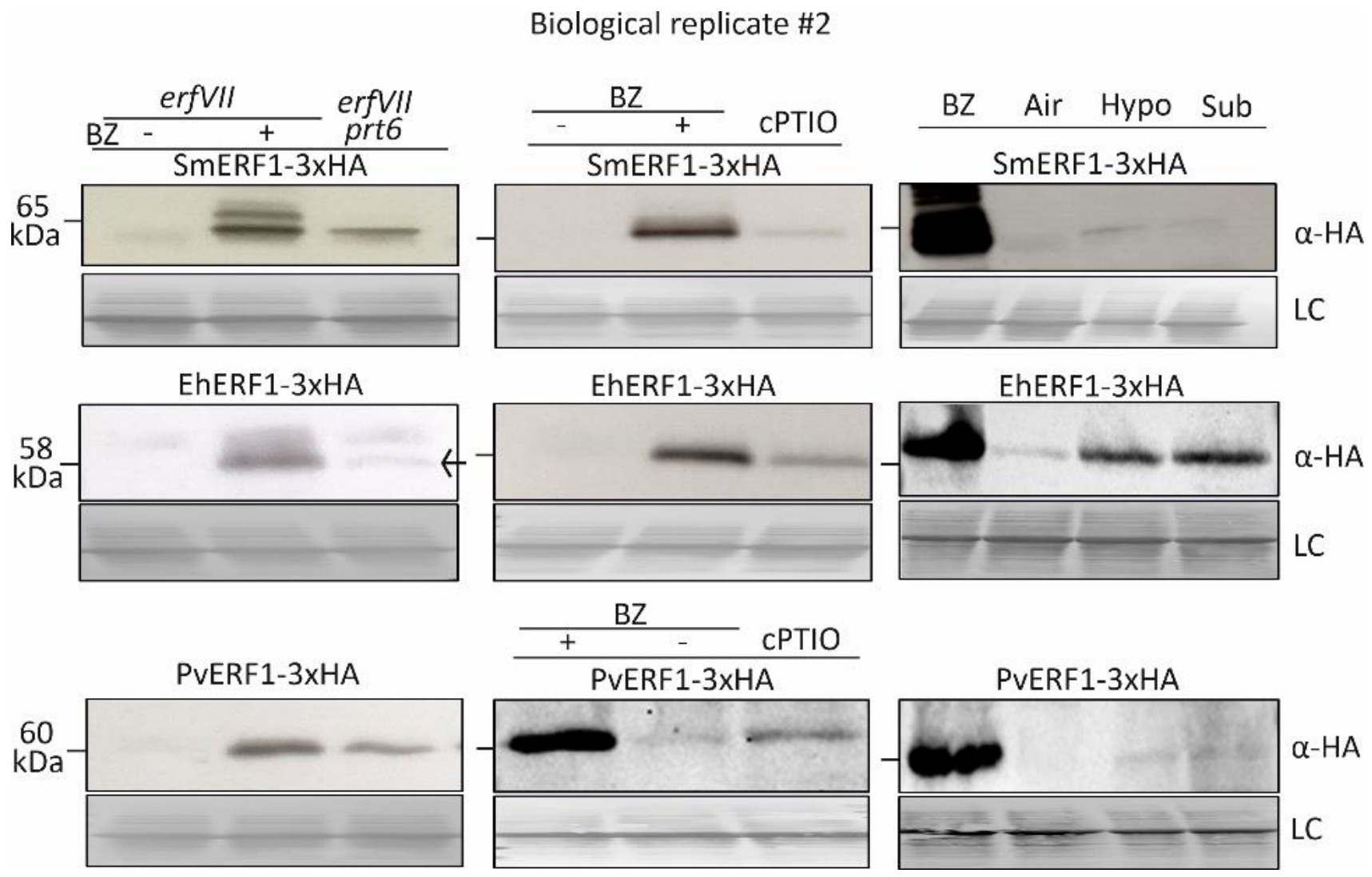

Supplemental Figure S13. Western blot analysis of SmERF1^{3xHA}, EhERF1^{3xHA} and PvERF1^{3xHA} abundance in control (DMSO), bortezomib (BZ), NO-scavenger (cPTIO), dark air (2h), dark hypoxia (2h, 0.1% v/v O_2) or dark submergence (1h) treated *erfVII* or *erfVII prt6* mutant 7-day-old whole seedlings (second biological replicate of what is shown in Fig. 3D. Loading control (LC) corresponds to a re-fitted and compacted image of the membrane after Ponceau staining. The unedited images are shown in **Supplemental Fig. S17-18**.

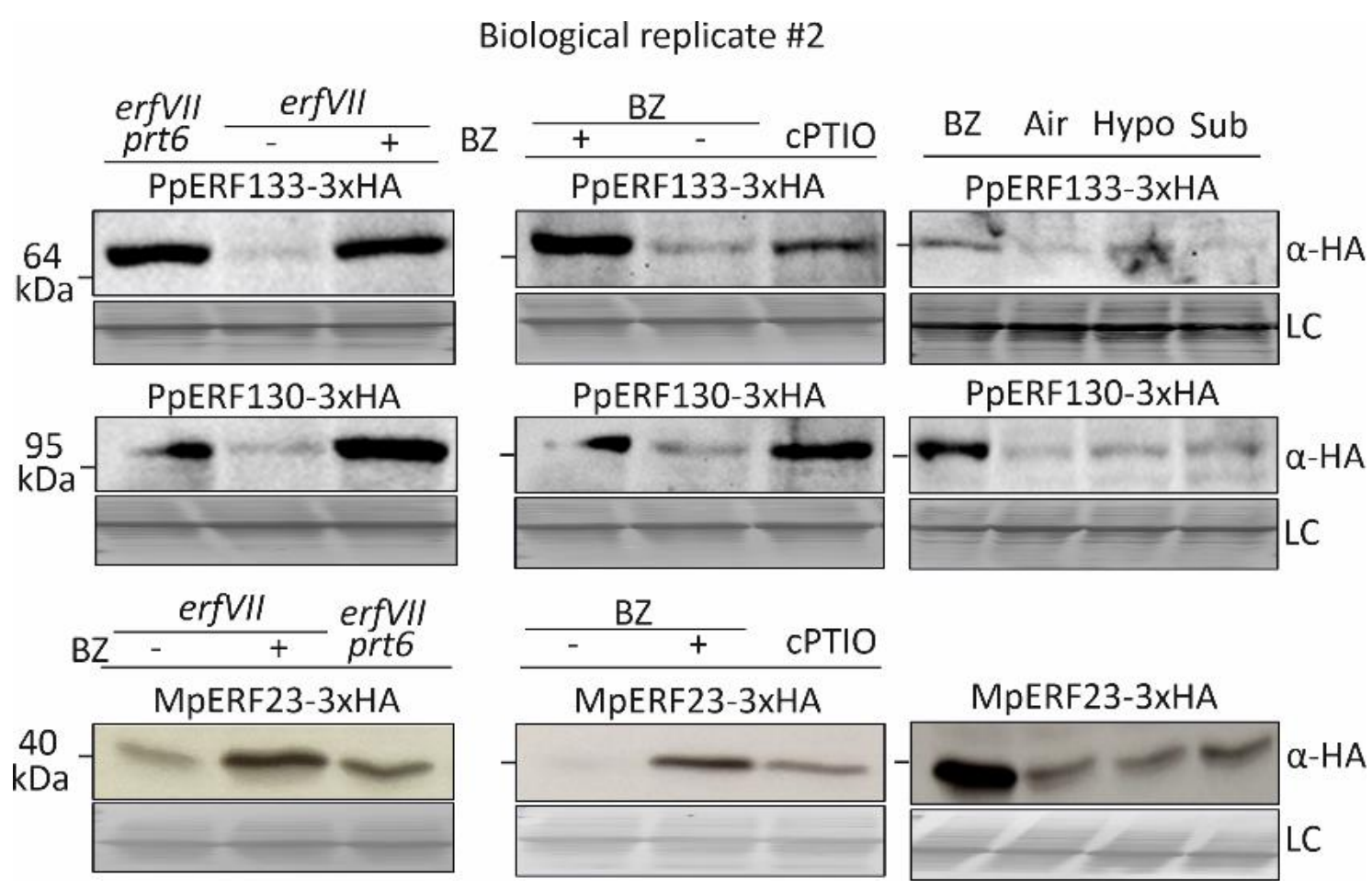

Supplemental Figure S14. Western blot analysis of PpERF133^{3xHA}, PpERF130^{3xHA} and MpERF23^{3xHA} abundance in control (DMSO), bortezomib (BZ), NO-scavenger (cPTIO), dark air (2h), dark hypoxia (2h, 0.1% v/v O_2) or dark submergence (1h) treated *erfVII* or *erfVII prt6* mutant 7-day-old whole seedlings (second biological replicate of what is shown in Fig. 4C). The black arrow indicates the expected protein size band. Loading control (LC) corresponds to a re-fitted and compacted image of the membrane after Ponceau staining. The unedited images are shown in **Supplemental Fig. S17-18**.

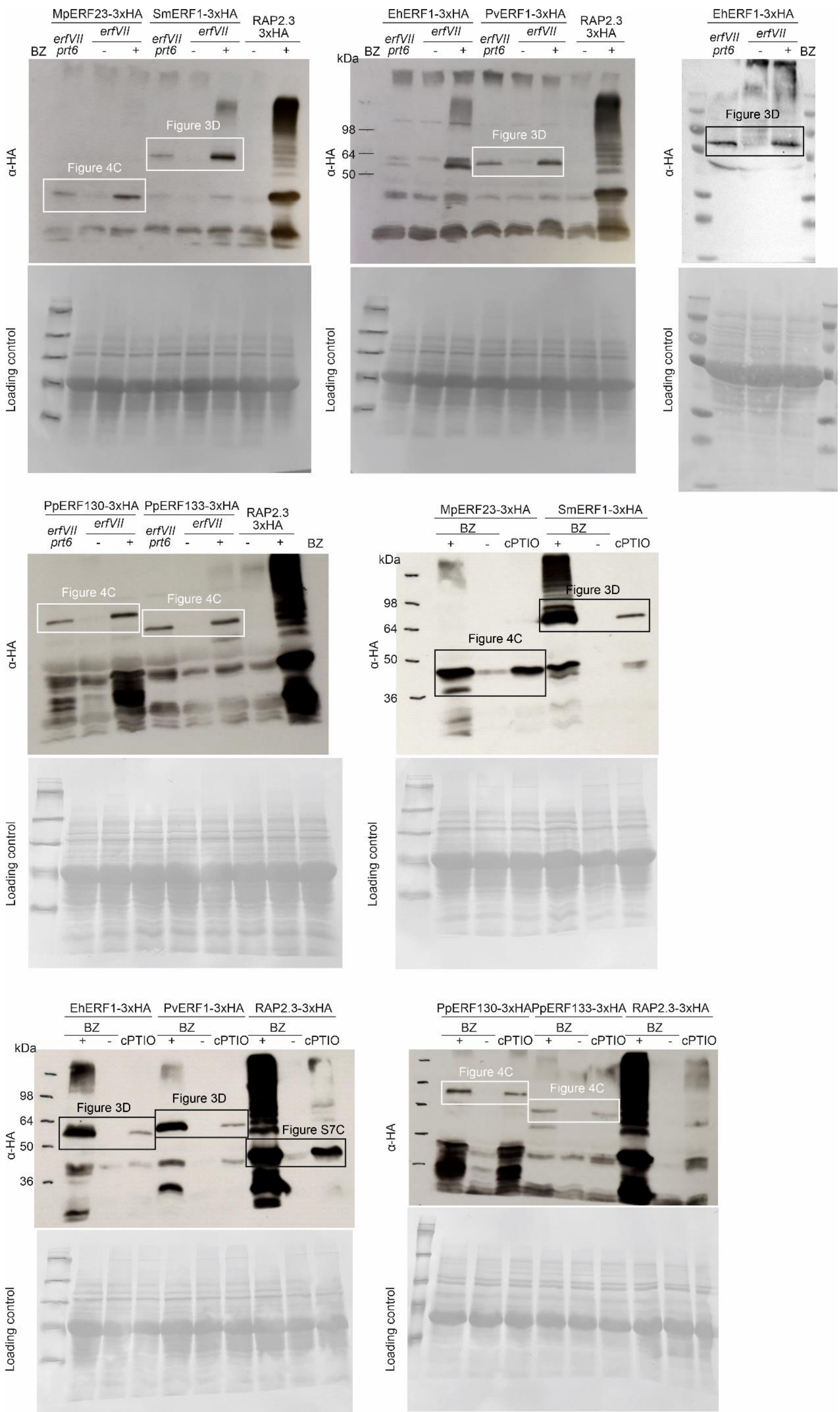

MpERF23-3xHA
SmERF1-3xHA
RAP2.3 3xHA
erfVII prt6
erfVII
BZ
Figure 3D
Figure 4C
α-HA
Loading control
EhERF1-3xHA
PvERF1-3xHA
kDa
98
64
50
36
PpERF130-3xHA
PpERF133-3xHA
cPTIO
RAP2.3-3xHA
Figure S7C

Supplemental Figure S15. Collection of original Western blots, and corresponding Ponceau staining, shown in Fig. 3D, 4C and S7C, related to cPTIO treatment and *erfVII prt6* background of various Cys2-ERFs.

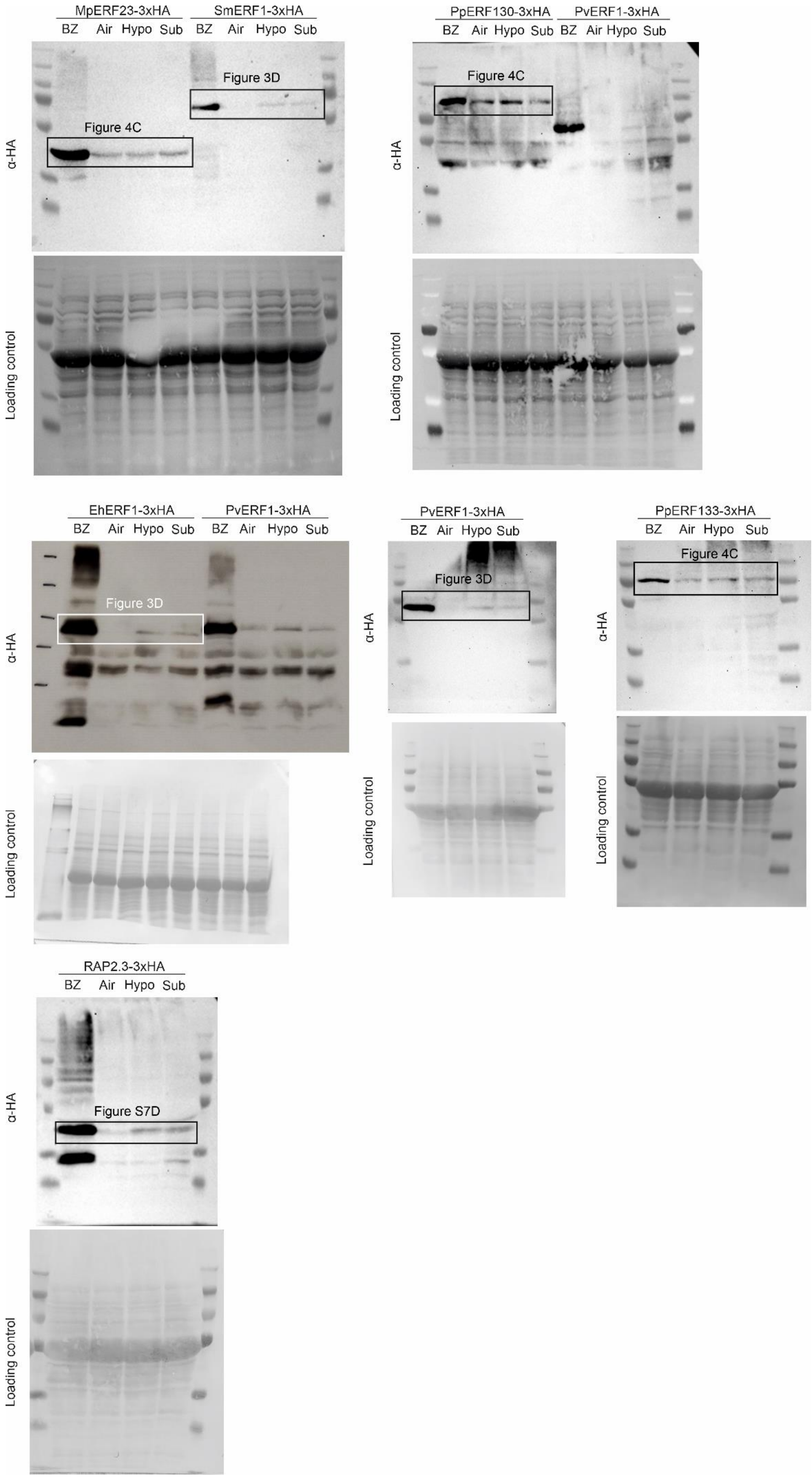
MpERF23-3xHA
SmERF1-3xHA
BZ Air Hypo Sub BZ Air Hypo Sub
Figure 3D
Figure 4C
α-HA
Loading control
PpERF130-3xHA
PvERF1-3xHA
BZ Air Hypo Sub BZ Air Hypo Sub
Figure 4C
α-HA
Loading control
EhERF1-3xHA
PvERF1-3xHA
BZ Air Hypo Sub BZ Air Hypo Sub
Figure 3D
α-HA
Loading control
PvERF1-3xHA
BZ Air Hypo Sub
Figure 3D
α-HA
Loading control
PpERF133-3xHA
BZ Air Hypo Sub
Figure 4C
α-HA
Loading control
RAP2.3-3xHA
BZ Air Hypo Sub
Figure S7D
α-HA
Loading control

Supplemental Figure S16. Collection of original Western blots, and corresponding Ponceau staining, shown in Fig. 3D, 4C and S7D, related to hypoxia and submergence treatments of various Cys2-ERFs.

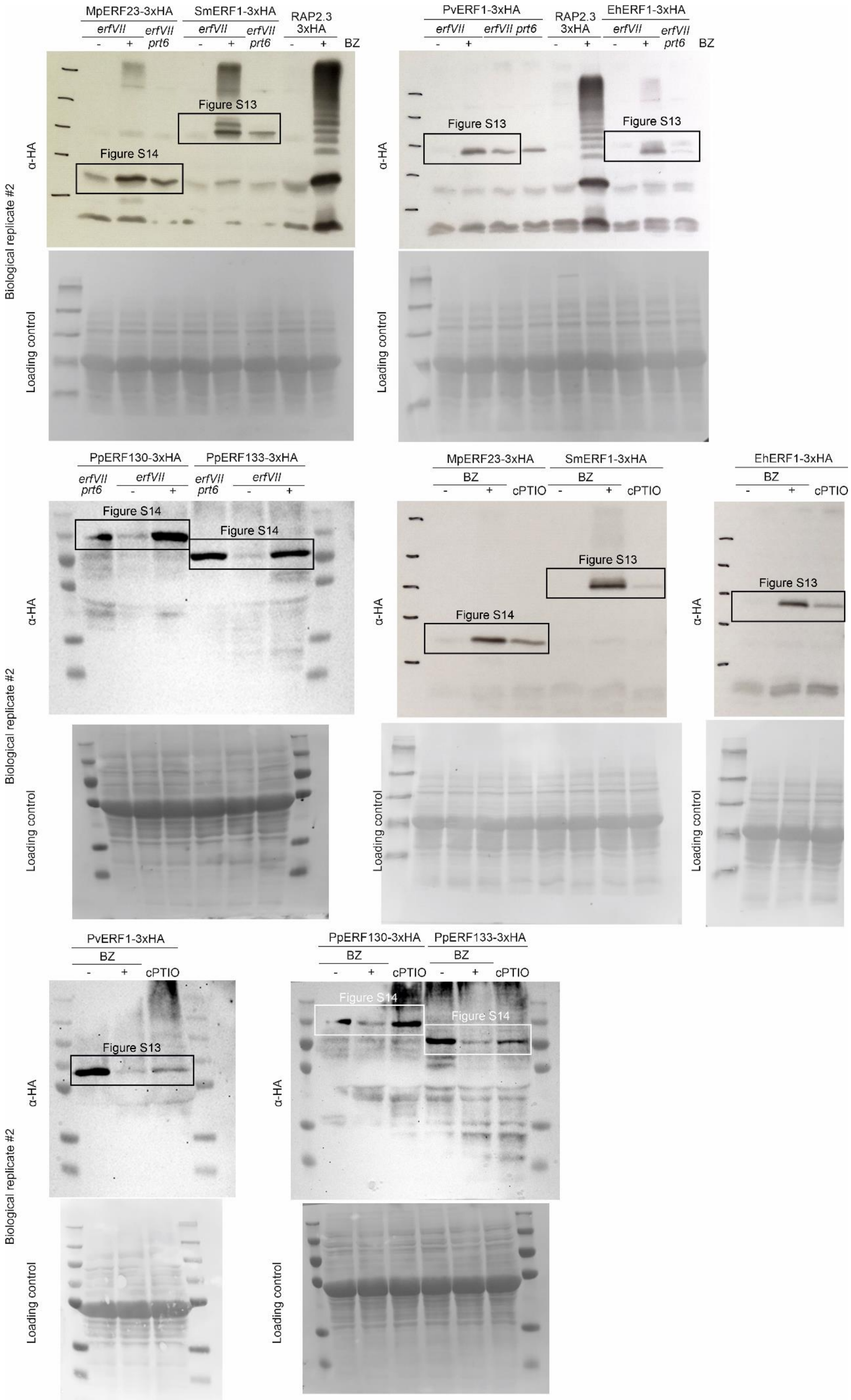

MpERF23-3xHA
SmERF1-3xHA
RAP2.3 3xHA
erfVII
erfVII prt6
BZ
- + - + - +
Figure S13
Figure S14
α-HA
Biological replicate #2
Loading control
PvERF1-3xHA
EhERF1-3xHA
erfVII prt6
PpERF130-3xHA
PpERF133-3xHA
cPTIO

Supplemental Figure S17. Collection of original Western blots, and corresponding Ponceau staining, shown in Supplemental Fig. S13 and S14, related to cPTIO treatment and *erfVII prt6* background of various Cys2-ERFs.

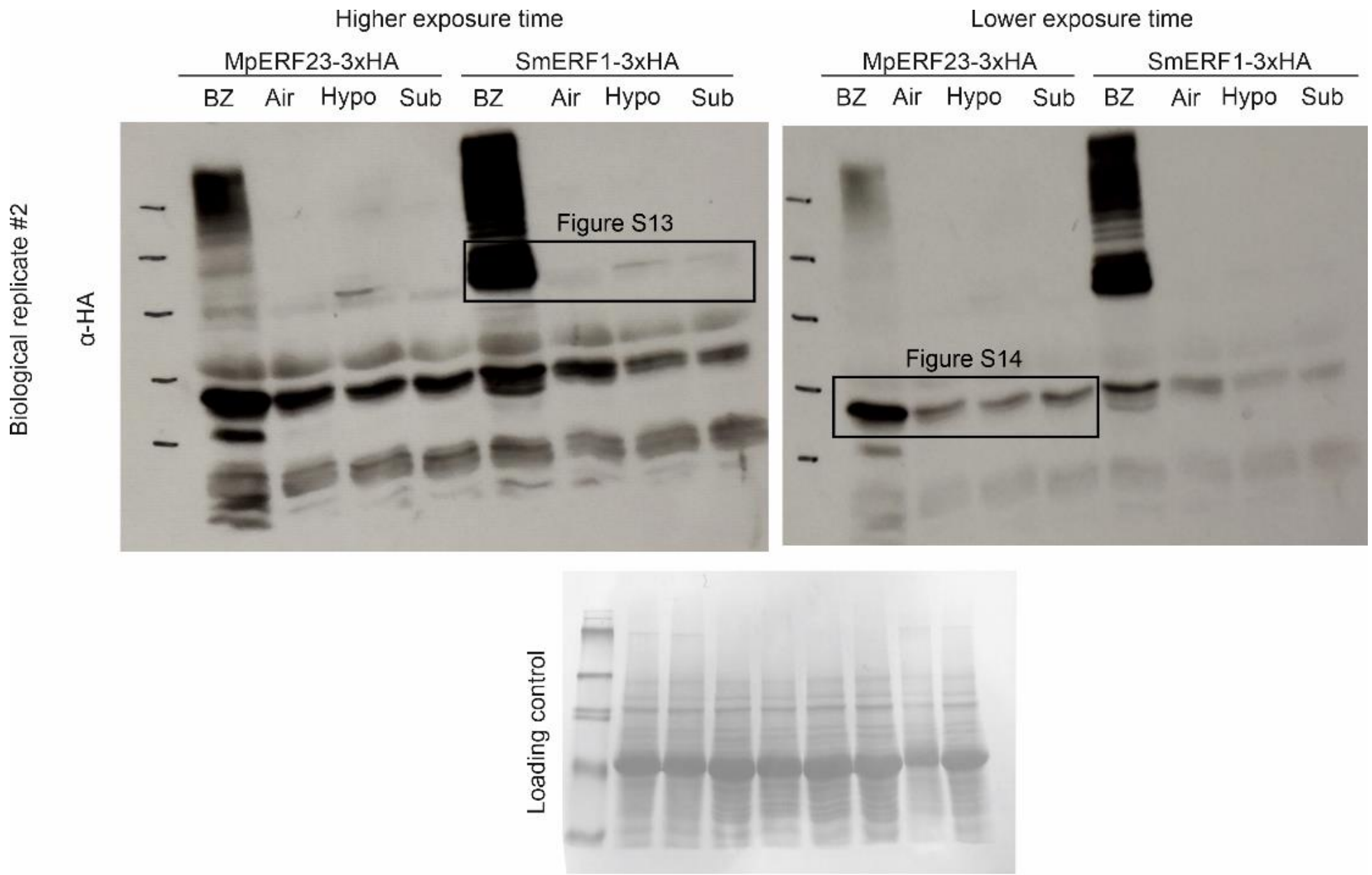
Higher exposure time
Lower exposure time
MpERF23-3xHA
SmERF1-3xHA
BZ Air Hypo Sub BZ Air Hypo Sub
MpERF23-3xHA
SmERF1-3xHA
BZ Air Hypo Sub BZ Air Hypo Sub
Biological replicate #2
α-HA
Figure S13
Figure S14
Loading control

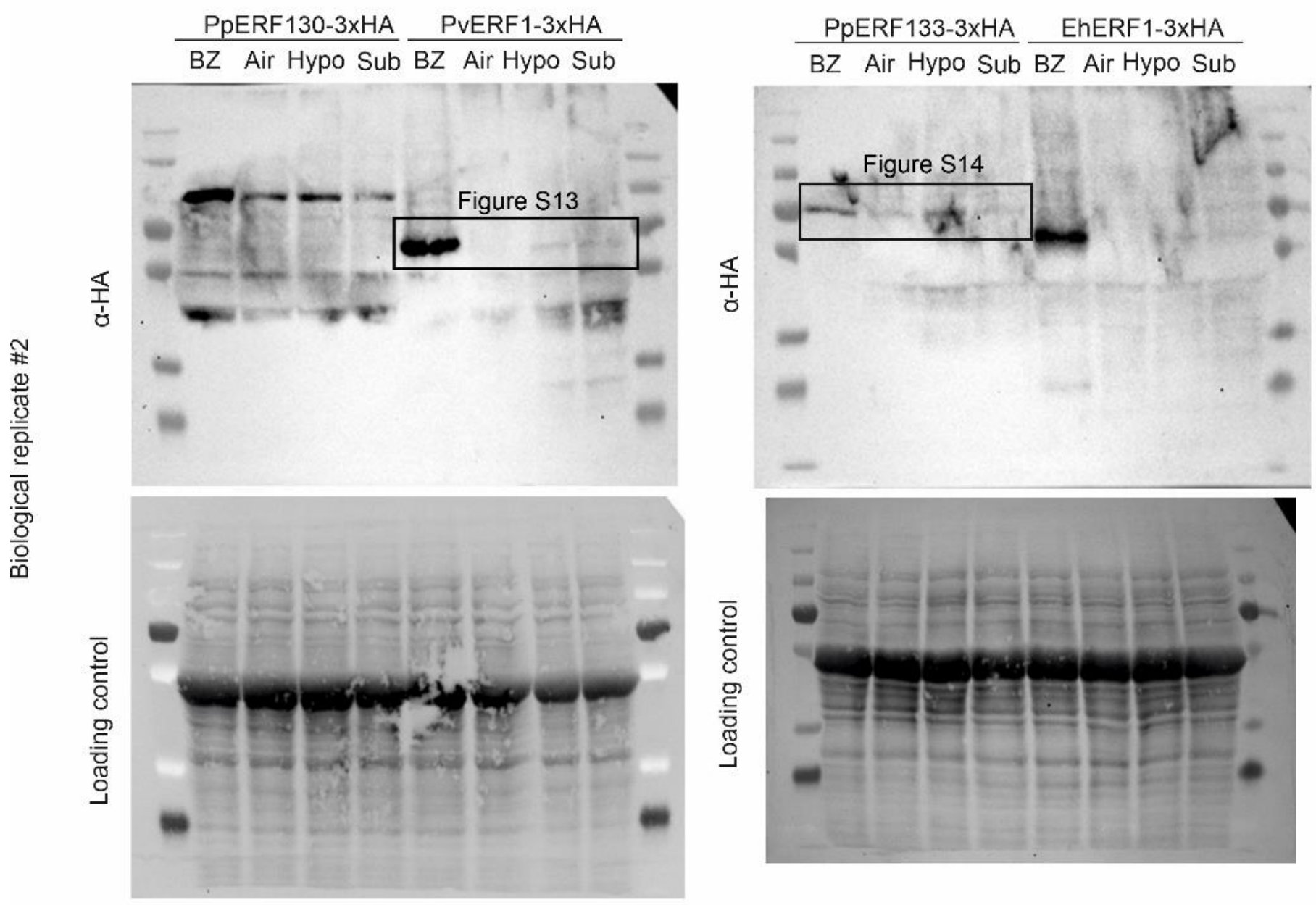
PpERF130-3xHA
PvERF1-3xHA
BZ Air Hypo Sub BZ Air Hypo Sub
PpERF133-3xHA
EhERF1-3xHA
BZ Air Hypo Sub BZ Air Hypo Sub
Biological replicate #2
α-HA
Figure S13
Figure S14
Loading control
Loading control

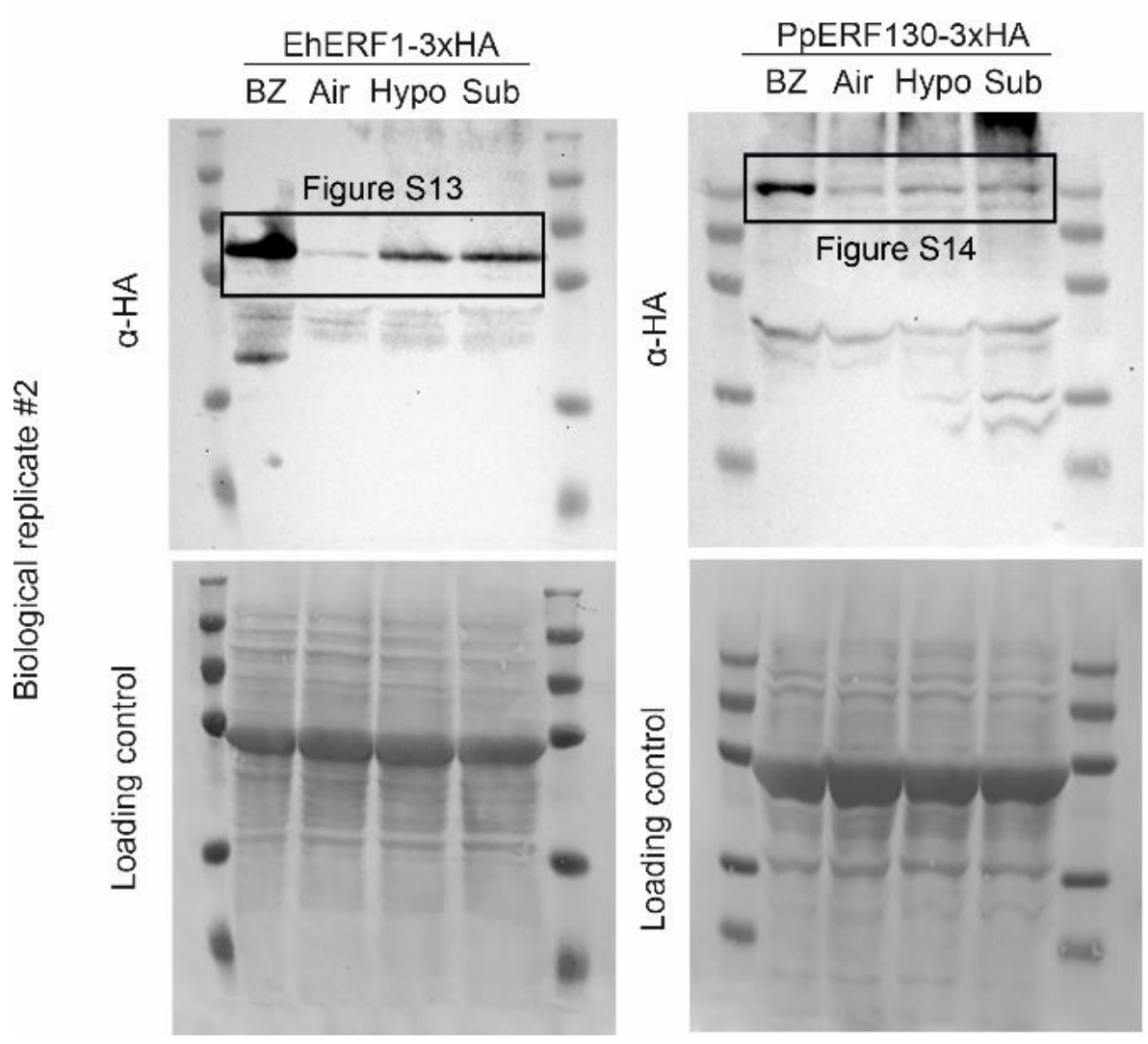
EhERF1-3xHA
BZ Air Hypo Sub
PpERF130-3xHA
BZ Air Hypo Sub
Biological replicate #2
α-HA
Figure S13
α-HA
Figure S14
Loading control
Loading control

Supplemental Figure S18. Collection of original Western blots, and corresponding Ponceau staining, shown in Supplemental Fig. S13 and S14, related to hypoxia and submergence treatments of various Cys2-ERFs.

Supplemental Tables

Supplemental Table 1. RNAseq analysis of *Marchantia polymorpha* in control (21% v/v O_2) and hypoxia (1% v/v O_2), for eight hours in the dark.
Supplemental Table 2. RNAseq analysis of *Physcomitrium patens* in control (21% v/v O_2) and hypoxia (1% v/v O_2), for eight hours in the dark.
Supplemental Table 3. RNAseq analysis of *Selaginella moellendorfii* in control (21% v/v O_2) and hypoxia (1% v/v O_2), for eight hours in the dark.
Supplemental Table 4. RNAseq analysis of *Equisetum hyemale* in control (21% v/v O_2) and hypoxia (1% v/v O_2), for eight hours in the dark.
Supplemental Table 5. RNAseq analysis of *Pteris vittata* in control (21% v/v O_2) and hypoxia (1% v/v O_2), for eight hours in the dark.
Supplemental Table 6. List of *M. polymorpha* genes associated to each orthogroup defined according the OrthoFinder method (Emms and Kelly 2015), alongside with the corresponding log_2FC and fold discovery rate (FDR), in hypoxia.
Supplemental Table 7. List of *P. patens* genes associated to each orthogroup defined according the OrthoFinder method (Emms and Kelly 2015), alongside with the corresponding log_2FC and fold discovery rate (FDR), in hypoxia.
Supplemental Table 8. List of *S. moellendorfii* genes associated to each orthogroup defined according the OrthoFinder method (Emms and Kelly 2015), alongside with the corresponding log_2FC and fold discovery rate (FDR), in hypoxia.
Supplemental Table 9. List of *E. hyemale* genes associated to each orthogroup defined according the OrthoFinder method (Emms and Kelly 2015), alongside with the corresponding log_2FC and fold discovery rate (FDR), in hypoxia.
Supplemental Table 10. List of *P. vittata* genes associated to each orthogroup defined according the OrthoFinder method (Emms and Kelly 2015), alongside with the corresponding log_2FC and fold discovery rate (FDR), in hypoxia.
Supplemental Table 11. List of *O. sativa* genes associated to each orthogroup defined according the OrthoFinder method (Emms and Kelly 2015), alongside with the corresponding log_2FC and fold discovery rate (FDR), in hypoxia.
Supplemental Table 12. List of *A. thaliana* genes associated to each orthogroup defined according the OrthoFinder method (Emms and Kelly 2015), and the corresponding log_2FC, along with the fold discovery rate (FDR), in hypoxia.
Supplemental Table 13. Number of orthogroups identified for each land plant group analysed in this study. Orthogroups are classified in 'conserved', 'gained', 'lost' and 'lineage specific'.
Supplemental Table 14. Number of genes that constitute the hierarchical clustering of conserved orthogroups from 33 species of land plants, including those studied in this work.
Supplemental Table 15. Percentage of number of genes, over the total number of genes, included in each of the fraction of conserved orthogroups identified for the 33 land plant species analysed in this study.

Supplemental Table 16. Number of orthogroups containing exclusively upregulated (log2FC>1) or downregulated (log2FC<-1) genes or simultaneously up- and down- regulated (-1<log2FC>1) genes under hypoxia. Gene cutoff 0 defines the proportion of genes in an orthogroup that need to be differentially expressed for the OG to be considered regulated.

Species name	$Log_2FC>1$	-1<Log2FC>1	$Log_2FC<-1$
M. polymorpha	263	7	283
P. patens	28	1	65
S. moellendorfii	11	0	148
E. hyemale	92	25	375
P. vittata	86	7	639
O. sativa	908	56	464
A. thaliana	541	43	492

Supplemental Table 17. $log_2FC<-1$ (Padj. <0.05) of conserved hypoxia-regulated orthogroups in the land plant species considered in this study. Orthogroups are classified according to the molecular function of the orthologous *A. thaliana* gene.

Supplemental Table 18. $log_2FC>1$ (Padj. <0.05) of conserved hypoxia-regulated orthogroups in the land plant species considered in this study. Orthogroups are classified according to the molecular function of the orthologous *A. thaliana* gene.

Supplemental Table 19. List of hypoxia-induced and repressed conserved orthogroups in which the corresponding A. thaliana protein belongs to the 'enzyme' class.

Supplemental Table 20. RNAseq analysis of *Arabidopsis thaliana* wild-tpe (Col-0) and *erfVII* pentuple mutant after hypoxia treatment time course (2 h and 10 h of 1% v/v O2 in the dark).

Supplemental Table 21. HRPE motif enrichment analysis in promoters of orthogroups, divided by species.

Supplemental Table 22. GCC-box motif enrichment analysis in promoters of orthogroups, divided by species.

Supplemental Table 23. MDM motif enrichment analysis in promoters of orthogroups, divided by species.

Supplemental Table 24. MYB-I motif enrichment analysis in promoters of orthogroups, divided by species.

Supplemental Table 25. MYB-II motif enrichment analysis in promoters of orthogroups, divided by species.

Supplemental Table 26. HRPE motif enrichment on promoter regions of conserved hypoxia-induced orthogroups.

Supplemental Table 27. GCC-box motif enrichment on promoter regions of conserved hypoxia-induced orthogroups.

Supplemental Table 28. MDM motif enrichment on promoter regions of conserved hypoxia-induced orthogroups.

Supplemental Table 29. MYB-I motif enrichment on promoter regions of conserved hypoxia-induced orthogroups.

Supplemental Table 30. MYB-II motif enrichment on promoter regions of conserved hypoxia-induced orthogroups.

Supplemental Table 31. List or protein sequences from different species of lycophytes, eusporangiate ferns, leptosporangiate ferns, gymnosperms and angiosperms, orthologous of *Arabidopsis thaliana* RAP2.12, used to build the phylogenetic tree of ERFVII transcription factors family in tracheophytes.

Supplemental Table 32. List of ERF transcription factors from *Marchantia polymorpha*, *Physcomitrium patens*, *Anthoceros agrestis*, *Selaginella moellendorfii* and *A. thaliana* used to build the phylogenetic tree shown in **Fig. 4**.

Supplemental Table 33. List of ERF transcription factors from *Chlamydomonas reinhardtii* and *Klebsormidium flaccidum*.

Supplemental Table 34. List of the best *Marchantia polymorpha* ERFVII (Mapoly0092s0058, MpERF18) orthologs retrieved in specific classes and orders of liverworts.

Supplemental Table 35. List of ERF transcription factor proteins starting with a cysteine in second position in different species of bryophytes.

Supplemental Table 36. List of forward and reverse primer sequences (5'-3') used for various cloning and RT-qPCR analysis in this study.

	Primer name	Gene name	Forward primer (5'-3')	Reverse primer (5'-3')
Cloning	PpERFVIII_SacII	*Pp3c7_1240V3.1*	gaaacccgcggtggttgttcacgagaagtagagaaa	ttttccgcggatattcatcggtaaagaaccc
	PpERFVIII_C2A		ccgcggtggtgcttcacgagaagta	tacttctcgtgaagcaccaccgcgg
	PpERFX_SacII	*Pp3c6_28370V3.1*	gaaacccgcggtggttgctttctgcaatcacttcca	ttttccgcggttcttgacacagagatgtgtc
	PpERFX_C2A		ccgcggtggtgcctttctgcaatca	tgattgcagaaaggcaccaccgcgg
	MpERFVII_SacII	*Mapoly0293s0001*	gaaacccgcggtggttgcagaatgaacaaaaggcta	ttttccgcggtcgaggtccatcgtatttgaa
	MpERFVI_C2A		cccgcggtggtgccagaatgaacaa	ttgttcattctggcaccaccgcggg
	SmERFVII_SacII	*73139*	gaaacccgcggtggttgccgtggtatcacctccgac	ttttccgcggccaagtaatctcttcgggatc
	SmERFVII_C2A		cccgcggtggtgcccgtggtatcac	gtgataccacgggcaccaccgcggg
	EhERF_SacII	*scaffold-JVSZ-2009360*	gaaacccgcggtggttgtggtggatccatcatttcc	ttttccgcgggtagtagtttgagaaatcttcaaag
	EhERF_C2A		cccgcggtggtgctggtggatccat	atggatccaccagcaccaccgcggg
	PvERF_SacII		gaaacccgcggtggttgcggcggatcgataatctcc	ttttccgcggctggccatcgtcaacagactt
	PvERF_C2A		cccgcggtggtgccggcggatcgat	atcgatccgccggcaccaccgcggg
	gbRAP2.12AD	*At1g53910*	gcgccgtctcgctcgttcggacaatggtgcaaaccctatg	gcgccgtctcgctcaaagctcagaagactcctccaatcat
RT-	sgUBQ	*At4g05320*	ggccttgtataatccctgatgaataag	aaagagataacaggaacggaaacatagt
	sgPDC1	*At4g33070*	cacagaatcttcaatgttcttacc	ccatgataaagcgtacatggaa

sgPCO2	*At5g39890*	cttcgagccgttttggatga	acgtcactaacggagatcgtcc
sgSmPDC	*157772*	aggtcgcctctcgtttggttg	agtgtcgttcttgcggagcttt
sgSmAHB	*80203*	gtctcacgcactcgtcgtct	acgacgtgcgtatgtcccat
sgSmADH	*145705*	acgcgattgagtgtgtcgga	ttgtgttgtcgtccaggcca
sgSm6PGD	*98399*	gctcatggatcccgagtttg	tccactcgctcgtaggtg
sgMpAct1	*Mapoly0016 s0137*	gagcgcggttactctttcac	gaccgtcaggaagctcgtag
sgMpPDC2	*Mapoly0160 s0004*	ggtcagcactccttacgctt	tagtcgttgaacacaggccc
sgMpETHE 1	*Mapoly0002 s0284*	tcatgccgggaggttctgga	cacggcaggcttgtcaggat
sgMpL2HG DH	*Mapoly0085 s0100*	ccacacgggaacgagagctg	ggtcggtgatctgcttcggg

Supplemental Table 37. Overview over Position-specific scoring matrices (PSSMs) for MYB-I and MYB-II *cis*-elements identified in this study. Matrices have been created using MEME version 5.5.7.

MYB I				MYB II			
a	c	g	t	a	c	g	t
0.5	0	0.17	0.33	0.25	0	0.75	0
0.33	0.17	0.17	0.33	0.125	0.125	0.5	0.25
0.33	0.5	0	0.17	0.1	0.1	0	0.8
0.67	0	0.17	0.17	0.6	0	0.2	0.2
0	0	1	0	0	0	1	0
0	0	0	1	0	0	0.2	0.8
0	0	0	1	0	0	0	1
0.5	0	0.5	0	0.5	0	0.5	0

Supplemental Files

Supplemental File S1. Multi alignment between protein sequences identified in *M. polymorpha*, *S. moellendorfii*, *E. hyemale*, *P. vittata*, *O. sativa* and *A. thaliana* belonging to the OG0000005 orthogroup.

Supplemental File S2. Multi alignment between protein sequences identified in *M. polymorpha*, *S. moellendorfii*, *E. hyemale*, *O. sativa* and *A. thaliana* belonging to the OG0000004 orthogroup.

Supplemental File S3. Multi alignment between protein sequences identified in *M. polymorpha*, *S. moellendorfii*, *E. hyemale*, *O. sativa* and *A. thaliana* belonging to the OG0007434 orthogroup.

Supplemental File S4. Multi alignment between protein sequences identified in *M. polymorpha*, *P. patens*, *S. moellendorfii*, *E. hyemale*, *O. sativa* and *A. thaliana* belonging to the OG0000715 orthogroup.

Supplemental File S5. Multi alignment between protein sequences identified in *M. polymorpha*, *P. patens*, *S. moellendorfii*, *E. hyemale*, *P. vittata*, *O. sativa* and *A. thaliana* belonging to the OG0000529 orthogroup.

Supplemental File S6. Multi alignment between protein sequences identified in *M. polymorpha*, *S. moellendorfii*, *E. hyemale*, *P. vittata*, *O. sativa* and *A. thaliana* belonging to the OG0000018 orthogroup.

Supplemental File S7. Multi alignment between protein sequences identified in *M. polymorpha*, *S. moellendorfii*, *E. hyemale*, *P. vittata* and *O. sativa* belonging to the OG0000016 orthogroup.

Supplemental File S8. Multi alignment between protein sequences identified in *P. patens*, *S. moellendorfii*, *E. hyemale*, *P. vittata*, *O. sativa* and *A. thaliana* belonging to the OG0000009 orthogroup.

Supplemental File S9. Multi alignment between *A. thaliana* five ERFVII protein sequences and the best ERF blast hits retrieved in bryophytes using AtRAP2.12 as query.

Supplemental File S10. Multi alignment between *A. thaliana* five ERFVII protein sequences and the best ERFVII orthologs identified in *Equisetum hyemale* and *Equisetum diffusum*, using AtRAP2.12 as query.

Supplemental Data S1. Unrooted phylogenetic tree of ERFVII orthologues in tracheophytes (lycophytes, eusporangiate ferns, leptosporangiate ferns, gymnosperms and angiosperms), with bootstrap values highlighted for each branch.

Supplemental Data S2. Unrooted phylogenetic tree of ERF proteins from bryophytes (*A. punctatus*, *M. polymorpha* and *P. patens*), lycophyte (*S. moellendorfii*) and angiosperm (*A. thaliana*), with bootstrap values highlighted for each branch.
